# Noise learning of instruments for high-contrast, high-resolution and fast hyperspectral microscopy and nanoscopy

**DOI:** 10.1038/s41467-024-44864-5

**Published:** 2024-01-25

**Authors:** Hao He, Maofeng Cao, Yun Gao, Peng Zheng, Sen Yan, Jin-Hui Zhong, Lei Wang, Dayong Jin, Bin Ren

**Affiliations:** 1 Pen-Tung Sah Institute of Micro-Nano Science and Technology, Xiamen University, Xiamen, 361005 China; 2 State Key Laboratory of Physical Chemistry of Solid Surfaces, Collaborative Innovation Center of Chemistry for Energy Materials (iChEM), The MOE Key Laboratory of Spectrochemical Analysis and Instrumentation, College of Chemistry and Chemical Engineering, Xiamen University, Xiamen, 361005 China; 3 Department of Biomedical Engineering, College of Engineering, Southern University of Science and Technology, Shenzhen, 518055 Guangdong China; 4 Department of Materials Science and Engineering, Southern University of Science and Technology, Shenzhen, 518055 China; 5 Institute for Biomedical Materials & Devices (IBMD), University of Technology Sydney, Sydney, NSW 2007 Australia; 6 Tan Kah Kee Innovation Laboratory, Xiamen, 361104 China

**Keywords:** Imaging studies, Computational nanotechnology

## Abstract

The low scattering efficiency of Raman scattering makes it challenging to simultaneously achieve good signal-to-noise ratio (SNR), high imaging speed, and adequate spatial and spectral resolutions. Here, we report a noise learning (NL) approach that estimates the intrinsic noise distribution of each instrument by statistically learning the noise in the pixel-spatial frequency domain. The estimated noise is then removed from the noisy spectra. This enhances the SNR by ca. 10 folds, and suppresses the mean-square error by almost 150 folds. NL allows us to improve the positioning accuracy and spatial resolution and largely eliminates the impact of thermal drift on tip-enhanced Raman spectroscopic nanoimaging. NL is also applicable to enhance SNR in fluorescence and photoluminescence imaging. Our method manages the ground truth spectra and the instrumental noise simultaneously within the training dataset, which bypasses the tedious labelling of huge dataset required in conventional deep learning, potentially shifting deep learning from sample-dependent to instrument-dependent.

## Abstract (web summary)

Improving signal to noise ratio of Raman spectra is vital for the application. Here, authors show a noise learning method that learns the noise feature of a spectrometer. This improves the signal to noise ratio and makes deep learning to be instrument dependent instead of sample dependent.

## Introduction

Raman spectroscopy is a label-free molecular fingerprint detection approach with high spectral resolution, allowing dynamics of multiple species during the biological or chemical processes to be simultaneously recorded^1,2^. These unique advantages make it possible to uncover the mechanisms of the biological or chemical events, and in some specially system even at single molecule level^3^, which leads to its increasingly important role in life sciences^4–7^, nanotechnology^8–11^, material sciences^12^, etc. However, the quality of the measured Raman spectra is generally hampered by the intrinsic low Raman scattering efficiency. Specifically, far less than 1 of 10^6∼10^ incident photons will experience Raman scattering for most molecules^13^. Even with plasmon enhancement, Raman signal remains vulnerable to the noise, especially in nanoscopic hyperspectral Raman imaging when the number of molecules contributing to the signal is small and drift becomes the determining factor of the spatial resolution with the decrease of the imaging area to several nanometers, as well as live-cell imaging where weak light illumination or fast data acquisition is required. Therefore, trade-offs must be made among the signal-to-noise ratio (SNR), data acquisition or imaging speed, and spectral and spatial resolution.

Computational methods provide a promising way to improve the data quality in microscopic imaging^14,15^. Typical examples include fast Raman imaging assisted by low-rank matrix approximation algorithms^16,17^, and fluorescence imaging assisted by deconvolution algorithms^18–20^. However, these methods heavily rely on the ideal presumption of the ground truth (GT) data. For instance, the signals are usually assumed to be low frequency in the conventional digital filters. Besides, parameter tuning introduces bias that impairs the robustness. Deep learning can statistically learn the task-specific knowledge from a huge dataset by training a multilayer neural network, and once the neural network is well-trained it does not need parameter tuning, which thus circumvents the above limitations of the traditional methods^21^. Therefore, deep learning has emerged as the mainstream method in a broad range of areas, such as classification^22,23^, subcellular segmentation^24–26^, and digital staining^27,28^. Deep learning has enhanced the spatial resolution of wide-field fluorescence images beyond the diffraction limit^29^, and improved the SNR of fluorescence^15,30–32^ and coherent Raman microscopy^14^ images at extreme low light level.

Nonetheless, the most widely used supervised deep learning requires a huge labelled dataset with high SNR, which cannot be easily obtained in most of the microscopy applications. This is particularly true for hyperspectral Raman microscopy and nanoscopy imaging where the signal is extremely weak. Simulated data must be used to mitigate this GT deficiency problem, but it is tedious and requires experienced professionals to produce the large labelled dataset. As a result, most of current deep learning models in Raman imaging applications are trained on a limited dataset^33,34^. Therefore, such *sample-dependent* deep learning is limited in enhancing Raman imaging towards high SNR, resolution, and speed.

**Fig. 1:**
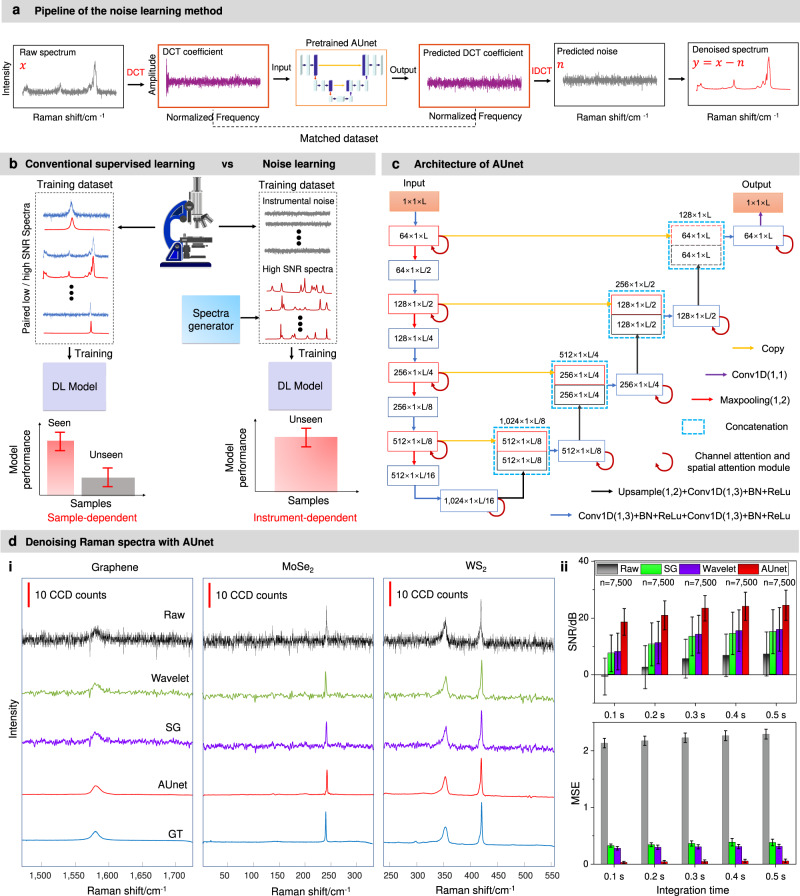
Principle of noise learning. **a** Pipeline of the denoising method proposed in this work. The raw spectrum is first processed by the discrete cosine transform (DCT) to produce coefficient as input to a pretrained 1-D attention U-net (AUnet) neural network to output the noise DCT coefficient. The predicted instrumental noise is obtained by the inverse discrete cosine transform (IDCT). The high SNR spectrum is finally obtained by subtracting the predicted instrumental noise from the noisy raw spectra. **b** Conventional supervised learning methods are sample-dependent, which require the matched low/high SNR experimental spectra to train the DL model to denoise the raw spectra, and work well for “seen” data but poorly for “unseen” data. Whereas, the NL method is instrument-dependent, which produces the low SNR spectra by combining the high SNR spectra through a physics-based spectra generator with the measured instrumental noise (Supplementary Fig. S1). It learns the instrumental noise distribution of a specific instrument and therefore it can perform well on the ‘unseen’ data. **c** The neural network architecture used throughout this work. 1-D U-net is used as the backbone, and the channel and spatial attention module are used to refine the features to improve the learning capability of the model. The color arrow indicates operation, and the number denotes the feature size. **d** (i) Representative denoising results of AUnet, SG and Wavelet methods on the spectra of three 2D materials acquired on a commercial Raman microscope. (ii) The quantitative metrics of the three methods. The data are presented as mean values ± standard deviation, and the sample size is *n* = 7500 for each group. More results can be found in the Supplementary Fig. S4.

In this article, we present a noise learning (NL) method that can largely expand the generalizability of deep learning, allowing a good estimation of the instrumental noise, and improve the sensitivity, spatial and temporal resolution of hyperspectral Raman microscopy and nanoscopy (Fig. 1a). Unlike the conventional supervised learning (CSL), we leverage a physics-based spectra generator to produce high SNR spectra using the prior knowledge of the line profile of Raman signal (Fig. 1b). Concurrently, we proposed a data generation method, which allows us to produce matched low SNR data with the estimated instrumental noise. In this way, NL is solely trained on the generated high and low SNR spectra dataset without the need of acquaring large amounts of spectra from real samples. Since the NL learns the intrumental noise, its performance is instrument-dependent. A modified 1-D deep convolutional neural network, called attention U-net (AUnet)^35^, is used to fit the mapping function from the noisy spectra to the instrumental noise. We demonstrate on commerial confocal Raman microscopy that NL can dramatically improve the SNR of the totally ‘unseen’ Raman spectra derived from real samples, such as 2D materials (graphene, MoS_2_) and live-cells. The SNR of the synthetic Raman spectra can be improved by up to 22.3 dB (approximately 10-fold), and the mean square error (MSE) error can be reduced by 149-fold with the NL method.

NL was further applied in hyperspectral tip-enhanced Raman spectroscopy (TERS, an important nanospectroscopy) imaging of a catalytic bimetallic Pd/Au(111) surface. While preserving the ability to reveal atomic structure dependent electronic properties of metal surfaces via the Raman peak shift of the probe molecule, NL improves the imaging speed that efficiently reduces the thermal drift problem of TERS imaging under the ambient and room temperature conditions. Together with simulation, we show that NL can improve the positioning accuracy, allowing a much accurate structure-spectra correlation. Last but not least, we show the versatility of NL on a commercial line-scan microscope from a different company with multi-modality imaging (Raman, fluorescence and photoluminescence). Thanks to the high SNR offered by the NL, the laser fluence for fluorescene imaging of live-cells can be significantly reduced by more than 43-fold, overcoming the detrimental photobleaching and phototoxity effects at high laser powers. This computation-aided ultra-low power fluorescence imaging allows stable, long-term monitoring of living biological systems. Therefore, the NL approach introduced here utilizes both the physical knowledge^36–38^ of the GT data and the property of microscopy instruments to overcome the limitations in conventional deep learning and microscopy imaging, thus shifting the field from being sample-dependent to instrument-dependent^39^.

## Results

### Noise learning with physics-based dataset

In a typical denoising scenario of CSL, the low- and high- SNR data pairs are acquired on interested samples. The performance of the deep learning model trained on such a dataset is, therefore, *sample-dependent*. As an alternative, NL introduces a physics-based spectra generator and an instrumental noise estimation method to produce the dataset. The instrumental noise (including photon shot noise, electronic readout noise, and dark current noise^40^) of the Raman instrument is measured using a Raman-inactive sample (flat Au film is used here), and then estimated from the measured spectra by a singular value decomposition (SVD) based method (See Supplementary Fig. S1 for more details). By doing so, the instrumental noise is approximately considered as additive. This is reasonable since the additive noise sources are the major contribution of the instrumental shot noise especially in the low SNR condition. Specifically, the shot noise arising from the detector and background of the instrument is the main source of the instrumental noise. The proposed method, thus, is dedicated to estimate this noise contribution and expected Raman (photoluminescence) signal from the measured spectra. More analysis of these noise sources can be found in the part of “Supplementary discussion-noise type analysis”. Although one expects noise to be random, we find that each Raman instrument exhibits a unique, instrument-specific, statistically stable noise pattern in the pixel-spatial frequency domain (Supplementary Fig. S1f, g, and Fig. S2). This forms the basis to use deep learning to fit the instrumental noise in the frequency domain.

In our NL approach, a pseudo Voigt function is first used for generating the GT Raman spectra as well as the baseline function (see Methods for more details). The corresponding low SNR spectra are simulated by adding the experimentally measured instrumental noise to the GT data. These high and low SNR datasets are now used for NL, in which we train the deep learning model to explicitly fit the pattern of the instrumental noise (Fig. 1b). For that we first perform discrete cosine transform (DCT) to transform the low SNR spectrum ($x$) to the pixel-spatial frequency domain, the DCT coefficient is then input to the pretrained AUnet model. Next, the predicted instrumental noise ($n$) is obtained by performing the inverse DCT (IDCT) with the predicted DCT coefficient. The high SNR spectrum can thus be obtained by subtracting the predicted instrumental noise from the low SNR data. In this way, the DCT coefficients of the low SNR spectra and its corresponding instrumental noise form the matched dataset to perform the supervised training.

The AUnet model is established based on a backbone of 1-D U-net, which has been intensively applied in a variety of applications because of its excellent performance^41^. On top of that, we further leverage the channel and spatial attention module, which have been proved efficient in improving the networks learning capability, to refine the model. In short, each feature is refined through the attention module before being input to the next layer (Fig. 1c).

### Restoration of simulated low SNR Raman spectra with noise learning

A well-trained model by NL approach is supposed to have the potential to fit the noise distribution of raw Raman spectra measured by a specific Raman instrument with different noise level. To verify this, we acquire the noise spectra of a commercial Raman microscope (LabRAM HR-Evolution, Horiba) with different integration time (0.1~0.5 s) on a flat Au film, while keeping other conditions unchanged. We train the AUnet model by NL approach with only 12,500 noise spectra (2500 spectra for each integration time), and the GT Raman spectra are randomly generated using the proposed physics-based method during each training step. To investigate the power of NL, we first calculate its performance on simulated Raman spectra, and compare the result with conventional noise reduction algorithms, such as Savitzky–Golay (SG) and wavelet transform (Methods). Since the deep learning model is trained using the randomly generated data, the quantified performance on the Raman spectra from real samples is unknown. Therefore, we further use a more realistic method (see Supplementary Fig. S3 for more detail) to generate the matched low- and high-SNR Raman spectra, using the raw data obtained from the real 2D material samples with the same Raman instrument. Representative results for thin layers of graphene, MoSe_2_, and WS_2_ are presented in Fig. 1d-i (first three columns). The SG and Wavelet methods provide spectra with improved SNR. In contrast, the AUnet leads to spectra with significantly improved SNR, which well restores the GT spectra, even if the SNR of the raw spectra was very low or negative (the signal power is smaller than that of the noise (Eq. 10)). To statistically quantify this observation, we calculate the MSE and SNR on a large testing dataset derived from the three 2D material samples (37,500 spectra in total, 12,500 spectra for each sample). As shown in Fig. 1d-ii (right panel), both MSE and SNR can be improved substantially with AUnet compared with the traditional denoising methods. Impressively, the averaged SNR can be improved up to 22.3 dB (about 10 folds) on the simulated graphene Raman spectra, and the mean MSE can be reduced by more than 149-fold compared with the raw data. Additional results of the restoration of raw spectra with different noise levels are shown in Supplementary Fig. S4. In all cases, AUnet-processed spectra reproduce well the raw spectra with much enhanced SNR. This indicates the good applicability of NL even though the same AUnet model, without retraining, is used for different samples. We note that such a strong noise reduction ability is difficult to be achieved using the conventional algorithms as indicated by the SNR and MSE bar chart in Fig. 1d-ii and Supplementary Fig. S4.

### Noise learning vs conventional supervised learning

**Fig. 2:**
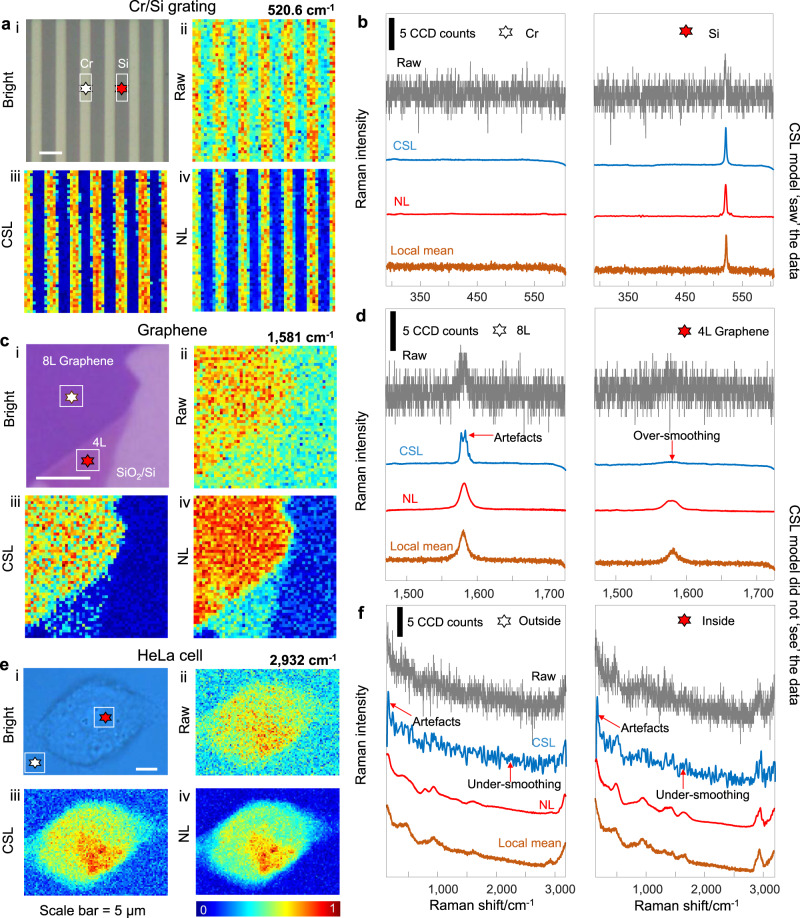
Comparison of conventional supervised learning (CSL) with noise learning (NL). The CSL model based on 1-D U-net is pretrained with a dataset that contains 12,500 Raman spectra acquired on Si sample patterned with Cr grating. The NL model used here is the same as that in Fig. 1d, which is trained solely with 12,500 instrumental noise spectra of the Raman instrument. **a**, **b** Raman imaging of Cr/Si grating sample. **a** (i) Bright field image. The white stripes are Cr and the grey substrate is Si; (ii) raw Raman image of the 520.6 cm^−1^ peak of Si; Raman image denoised by the CSL (iii); and NL (iv) methods. **b** Left and right panels are Raman spectra at the position marked by the white and red stars on the bright field image in (**a**-i), corresponding to the location of Cr and Si, respectively. Raw spectra, spectra processed by CSL and the NL methods, and the local mean spectra averaged over the white rectangles marked in (**a**) are shown for comparison. **c**, **d** Raman imaging of graphene sample with four- and eight-layer regions. **e**, **f** Raman imaging of HeLa cell sample. The results indicate that CSL only works well for the “seen” sample of Cr/Si grating, but fails when applied to “unseen” samples of graphene and HeLa cell, whereas the performance of NL works well for all samples measured with the same instrument. The spectra were acquired on LabRAM HR-Evolution.

Having shown the power of NL on the restoration of simulated Raman spectra and its advantages over the traditional denoising algorithms, we further ask whether it works well for the spectra acquired on real samples, and if it can be a superior alternative to the CSL approach. For that we train a 1-D U-net by the CSL method (Fig. 1b, left) with a dataset produced by the same confocal Raman microscope. The training dataset contains 12,500 low- and high- SNR spectra pairs obtained by the method described in Supplementary Fig. S3 with a silicon sample covered by Cr grating (Fig. 2a). The well-trained CSL model is then used to restore the raw spectra derived from different samples, both for ‘seen’ and ‘unseen’ ones, while the NL model used here is the same as that used in Fig. 1d without further retraining.

Expectedly, the CSL model performs well on the data it ‘saw’ during the training stage as shown in Fig. 2a, b. Three Raman images of the characteristic peak of Si at 520.6 cm^−1^ is shown in Fig. 2a. Compared with the raw spectra image (Fig. 2a-ii), the restored images after pixel-wise denoising either from the CSL (Fig. 2a-iii) or NL (Fig. 2a-iv) methods show much improved contrast, and match well with the bright field image (Fig. 2a-i). Figure 2b shows representative raw, CSL, and NL restored spectra for the location of Cr and Si points (marked by stars in Fig. 2a), indicating that both CSL and NL work well for this specific sample. This is verified by comparing the restored spectra with the local mean spectra averaged over the small area marked by white rectangle in Fig. 2a.

We further apply the CSL model trained on the Cr/Si grating sample to the ‘unseen’ samples such as graphene (placed on a Si/SiO_2_ substrate) and HeLa cell, and find that its performance deteriorates seriously. Specifically, the Raman image of the G-band (1581 cm^−1^) processed by the CSL (Fig. 2c-iii) can hardly distinguish the 4-layer (4 L) graphene region from the substrate (the layer number is confirmed by the height profile acquired with atomic force microscopy, AFM). Impressively, this slight difference can be easily resolved in the NL processed Raman image (Fig. 2c-iv). Moreover, the Raman spectra in Fig. 2d show that NL can faithfully restore the intrinsic peak feature by comparing to the local mean spectra, for both 8 L and 4 L graphene regions. In contrast, the CSL-spectra show obvious artefacts and over-smoothening when being applied to this “unseen” sample. Similar experiment is then carried out on a HeLa cell sample. In this case, the NL processed Raman images (Fig. 2e-iv) shows superior SNR and contrast over the CSL one (Fig. 2e-iii). Particularly, CSL-spectra present more artefacts and under-smoothening problems in dealing with the Raman spectra acquired both outside (Fig. 2f, left) and inside (Fig. 2f, right) the cell. Instead, the NL-spectra agree well with the local mean spectra (averaged over 11×11 pixels marked by the white rectangle in Fig. 2e), as clearly seen for the CH abundant band around 2800-3000 cm^−^^1^. These results convincedly demonstrate that the NL approach allows a single deep learning model to be applied in the restoration of a variety of Raman spectra acquired by the same Raman microscope, which cannot be accomplished via the CSL method.

The high SNR provided by NL endow it with the potential to perform low-laser-power measurements that can largely avoid laser damage effects. To test this hypothesis, we design a series of imaging experiments using the Cr/Si grating sample. The laser power is sequentially decreased from 0.2 mW to 0.02 mW with the pixel-dwell time fixed as the instrument-limit minimal value of 0.1 s/pixel. The same AUnet model trained by the NL approach used above, without any retraining, is then used to restore the imaging. The results shown in Supplementary Fig. S5 suggest that the AUnet substantially reduces the laser power limit down to 0.05 mW, at which the Cr and Si regions are distinguishable. The image contrast is even better than the raw image obtained at a power of 0.2 mW, demonstrating that the power of AUnet in Raman microscopy.

### AUnet assisted fast nanometer-resolution hyperspectral Raman imaging with TERS

Encouraged by the powerful noise suppression ability of NL, we next investigate whether this technique can be applied in different modality of Raman spectroscopy. TERS combines Raman spectroscopy and scanning probe microscopy to simultaneously acquire the topographic and chemical information of a sample (see Supplementary Fig. S6 for the TERS setup). The ultra-high sub-nanometer spatial resolution of TERS allows single molecular chemical imaging with fingerprint information under ultrahigh vacuum and ultralow temperature conditions^8,42^. The imaging quality of TERS is, unfortunately, limited by the SNR as the number of molecules contributing to the signal is extremely low, and affected by system stability as the imaging area is small. Despite being enhanced by the localized surface plasmon resonance and the lightning-rod effect^43^, the TERS signal is still too weak to allow fast imaging. While increasing the integration time can improve the SNR, it will significantly increase the accumulated sample-tip drift particularly under ambient and room temperature conditions, where many important applications, such as nanoscale catalysis study, are relevant. The drift can result in serious distortion of the Raman image, hampering the extraction of meaningful information associated with nanoscale structural features. There has to be a trade-off between imaging quality and image distortion. Here we conduct the hyperspectral TERS imaging with the same Raman instrument used above, but now integrated with scanning tunneling microscope (STM) for TERS. This allows us to use the same AUnet model to improve the SNR of the TERS spectra and shorten the imaging time, mitigating the distortion problem.

**Fig. 3:**
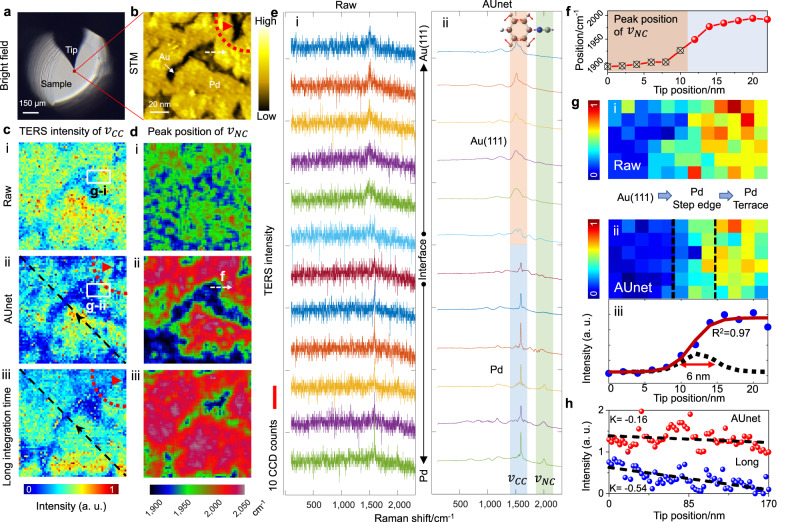
AUnet assisted fast nanometer-resolution hyperspectral TERS imaging of a Pd/Au(111) catalyst adsorbed with PIC molecule. **a** Bright field image of the sample and the gold tip. The region for TERS imaging (120 nm × 120 nm) is localized underneath the nanoscale-region below the tip. **b** STM topographic image of sub-monolayer Pd deposited on a Au(111) surface obtained by the gold tip. Scanning direction: from bottom to top. **c**, **d** TERS imaging of ${v}_{{CC}}$ band intensity (**c**) and ${v}_{{NC}}$ peak position (**d**). (i): raw data of fast imaging with an integration time of 100 ms/pixel. (ii): the denoised image by AUnet. (iii): the long-integration-time imaging with 1 s/pixel. **e** TERS spectra along the white dashed line in (**b**). (i): raw data. (ii): the denoised data by AUnet. Inset: the molecular structure of PIC and the vibrational mode of ${v}_{{CC}}$ and ${v}_{{NC}}$. **f** The profile of ${v}_{{NC}}$ frequency along the white dashed line in (**d**-ii). **g** panels i, ii: Zoom-in images of the white rectangles in (**c**). The spatial resolution of the TERS measurement is estimated to be 6 nm from the AUnet-denoised image (panel iii, see details in supplementary Fig. S12). **h** Red and blue balls are the pixel intensities along the black dotted lines in (**c**-ii) and (**c**-iii), respectively. The dash lines are the fits by a first-order polynomials $(y={Kx}+b)$, in which K denotes the slope of the fitted line.

We characterize the surface properties of bimetallic catalysts that have attracted tremendous attention in recent years^44,45^. Identifying the atomic scale catalytically active sites is central to catalysis but remains challenging for conventional micro-spectroscopy with diffraction-limited spatial resolution. The catalysts are produced by electrochemical underpotential deposition of a submonolayer Pd on the surface of Au(111) single crystal. The nanoscale region (120 nm × 120 nm) below the tip (Fig. 3a) is imaged using the STM to obtain a surface height topography of the Pd/Au(111) surface. The STM image in Fig. 3b shows that the deposited Pd and the Au substrate can be clearly resolved^46^. To reveal the catalytic activity at different surface sites, we use phenyl isocyanide (PIC) as the Raman probe molecule. We have shown that PIC can be oxidized at the Au surface but remains unreacted at the Pd terrace sites^46^. Thus, distinct Raman spectra can be observed at different surface sites, allowing a nanoscale imaging of the catalytic activity on the surface. To obtain high-fidelity TERS imaging result, the pixel-dwell time must be as short as possible to reduce the drift effect. Here we set it as 100 ms/pixel (24 min in total for imaging the 120 nm × 120 nm region with 2 nm/pixel), which is the lower readout limit of the EMCCD in low readout noise mode. As expected, such a short integration time leads to an low SNR of the raw spectra shown in Fig. 3e, even though slight difference can be seen for the spectra acquired on Au and Pd surfaces. As a result, the TERS imaging of the C = C stretching band (*ν*_CC_, 1587 cm^−1^) appears very blurry (Fig. 3c-i). Strikingly, the AUnet processed image (Fig. 3c-ii) presents sharp contrast between the Au and Pd regions that well correlates with the topographic image (Fig. 3b). Moreover, the difference of the TERS spectra at the Pd and Au surface sites along the white dashed line marked on the STM image can be easily distinguished after restoration by AUnet (Fig. 3e-ii), but not with the raw data (Fig. 3e-i).

Another important spectral feature of PIC is that the N ≡ C stretching frequency (*ν*_NC_, ∼1995 cm^−1^) is sensitive to the electronic structure of the catalyst surface^46,47^. Specifically, the peak position of the *ν*_NC_ band will red-shift at the interface of Pd and Au(111). Due to the weak signal of this band, the peak position is difficult to analyze in the raw spectra (Fig. 3e-i). Remarkably, it can be well resolved after the restoration of AUnet (Fig. 3e-ii). Such a subtle peak shift at different surface sites can be more clearly observed in the AUnet processed image of the frequency of *ν*_NC_ band (Fig. 3d-ii). Here, the green color indicates lower ν_NC_ frequency at Pd step edge sites compared with the red color (higher *ν*_NC_ frequency) at Pd terrace sites, nicely mapping the interface of Pd and Au(111), revealing the distinct electronic properties of the step edge Pd atoms at the interface. Such meaningful information is hardly analyzed with the raw data (see image in Fig. 3d-i, and spectra in Fig. 3e-i). The *ν*_NC_ frequency along the white dashed line marked on Fig. 3d-ii is presented in Fig. 3f. A red shift of about 70 cm^−1^ is seen when the tip moves from Pd terrace to step edge, consistent with previous report^46^.

The spatial resolution and positioning accuracy of a TERS image is crucial for obtaining a clear local structure-property correlation. The zoom-in TERS intensity images of the *ν*_CC_ band (marked by the white rectangles in Fig. 3c) are shown in Fig. 3g. In this zoom-in region, the left part is the Au(111) surface and right part is Au(111) covered by monoatomic layer Pd with an edge structure in-between as seen in the STM image (Fig. 3b). The TERS intensity in the raw data (Fig. 3g-i) spreads rather randomly, which does not match well with the STM image. In contrast, the transition from Au(111) to Pd regions (left to right) can be clearly resolved in the AUnet-denoised image (Fig. 3g-ii), implying that the positioning accuracy has been improved by denoising. We have simulated noisy and AUnet-processed Raman images containing an edge structure (supplementary Fig. S10), which reproduces the experimental result well. Further analysis reveals that AUnet denoising substantially improves the positioning accuracy of the TERS imaging result (supplementary Fig. S11 and supplementary discussion). The spatial resolution has been improved from 7.4 to 6 nm (Fig. 3g-iii) for the data presented in Fig. 3g (see “Evaluation of spatial resolution of TERS” of the Methods part and supplementary Fig. S12 for details). Analysis of more regions indicates that the spatial resolution is improved by 1.2–2 times after denoising (Supplementary Fig. 13).

The spatial resolution enhancement may benefit from an increase of spectral resolution since our denoising method reduces the noise for the spectra in each pixel of the TERS image. We have analyzed the spectral resolution before and after denoising by simulation, and the statistic result suggests that denoised spectra can give better spectral resolution than the noisy counterpart, if a large number of spectra are analyzed (Supplementary discussion and Figs. S16 and 17). This may potentially increase the spatial resolution. A more detailed study is, however, needed to address the interplay between spectral and spatial resolution enhancement.

To fully illustrate the advantage of AUnet assisted fast TERS imaging, we also imaged the same area with a long integration time of 1 s/pixel (Fig. 3c-iii). Although the SNR is improved, the overall data acquisition time is also greatly extended to 80 min, magnifying the instrument drift, and thus leading to two detrimental effects. First, the structure in the image is distorted due to the drift that shifts the imaging area even out of the set region. For instance, the Au hole at the upper-right corner of the STM image marked by the red curve (Fig. 3b) is totally outside of the image obtained with long integration time (Fig. 3c-iii), while it is faithfully recorded on the AUnet processed image (Fig. 3c-ii). Similar distortion can be seen in the center Au hole region. The second effect is the signal weakening due to the drift of the plasmon nanocavity (formed between the tip and the sample) with respect to the focus of the objective lens, thus reducing the signal collection efficiency^48^. This is reflected by the gradual weakening of the peak intensity of the *ν*_CC_ band along the black dashed line (Fig. 3c-iii, the arrow indicates the scanning sequence). Such a signal weakening effect is considerably reduced in the fast imaging assisted by AUnet. To quantify this observation, we plot the TERS intensity profiles along the black dashed lines in Fig. 3c-ii and Fig. 3c-iii as red and blue filled circles in Fig. 3h, respectively. The data are then fitted using a first order polynomial (Fig. 3h, black dotted line). The slope is −0.16 for the AUnet-processed image and −0.54 for the long integration one. This confirms the larger signal degradation at a longer total acquisition time (24 min vs 80 min). Conclusively, the AUnet-denoising is highly beneficial to allow fast imaging with significantly improved SNR and enhanced positioning accuracy, making it ideal for nanoscale imaging such as in TERS, where the signal is extremely weak and a clear local structure-property correlation is desired.

### AUnet assisted multi-modality line-scan imaging

To demonstrate the generalizability of the NL on different instruments, we train a second AUnet model on another commercial line-scan Raman microscope (Raman-11, Nanophoton Inc). The AUnet model is trained by the NL approach with 400,000 instrumental noise spectra (see Methods for more detail about the dataset) of the target microscope by the method presented in Supplementary Fig. S1. The well-trained AUnet model is then used for restoring the line-scan fluorescence and Raman imaging of HeLa cells and photoluminescence imaging of 2D materials acquired with the same instrument.

**Fig. 4:**
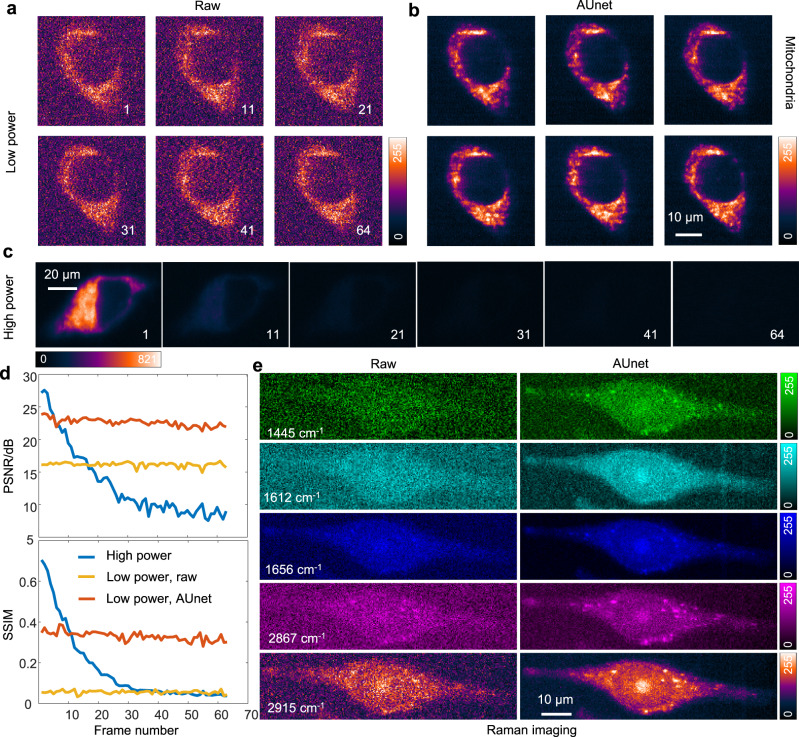
AUnet assisted fast live-cell fluorescence and Raman imaging using a different line-scan microscope. A same AUnet model is used for both fluorescence and Raman spectra denoising without retraining. **a** Raw fluorescence images obtained by continuously multi-frames line-scan imaging of HeLa cell with mitochondria label and a low laser power of 8.8 µW. **b** Corresponding high SNR images processed by AUnet. **c** Fluorescence images obtained with a high laser power of 381 µW, and the other experimental conditions are the same as (**a**). The SNR dramatically decreases after 20 frames continuous imaging, indicating the photobleaching of the fluorophores under high-power laser illumination. The frame numbers during imaging are indicated in (**a**) and (**c**). **d** The PSNR and SSIM curves of images in (**a**–**c**). For each image sequence, the first image frame is used as the ground truth to compute the PSNR and SSIM of the following image frames. **e** The line-scan Raman images of a HeLa cell using five characteristic Raman peaks of lipids and proteins.

A key issue in fluorescence imaging of live-cell is the possible photobleaching of the fluorophore during the long-term imaging due to limited photon budget. Concurrently, the laser illumination will introduce phototoxicity to the fragile biological samples. Therefore, low laser power is preferred, but with a compromise of decreased SNR. Here we demonstrated that such limitations can be substantially eliminated by the noise reduction technique based on AUnet. We continuously imaged the HeLa cells that were labelled with mitochondria dye using low (8.8 µW, Fig. 4a, b) and high (381 µW, Fig. 4c) laser power. The photobleaching effect is negligible in the low power excitation condition, and the raw images (Fig. 4a) with weak SNR are successfully restored after pixel-wise denoise with AUnet (Fig. 4b). For the high laser power case, serious photobleaching occurred after 20 frames, and the fluorescence signal becomes unobservable at the 64^th^ frame (Fig. 4c). This can be more clearly seen in Supplementary Fig. S7 and Supplementary Movies 1-3 for the image of a continuous sequence of the frames of low and high laser power cases. For quantifying these observations, we use the first frame as the GT data for each image sequence, and calculate the peak signal-to-noise ratio (PSNR) and structural similarity (SSIM). As shown in Fig. 4d, despite being stable, the two indicators of the raw images acquired with low laser power are too poor to be analyzed (yellow curves). The AUnet substantially improves both indicators, facilitating high-quality continuous imaging of the live-cells and downstream analysis (red curves). In contrast, the two indicators dramatically decrease in the high-power case (blue curves) and become even lower than that of the low power case after the 10^th^ frame in terms of the PSNR performance. To demonstrate the robustness of this method, the AUnet-assisted fast continuous fluorescence imaging of HeLa cells that are labelled with membrane and lysosome dyes are also provided (Supplementary Fig. S8). Moreover, additional results of the successful application of the AUnet on restoration of the photoluminescence imaging of four different 2D materials are also given in Supplementary Fig. S9. Importantly, AUnet shows superior noise reduction capability for a wide range of wavelength, as shown in Supplementary Movie 4 for a comparison of the photoluminescence imaging of WS_2_ from 600 to 630 nm.

We emphasize that our AUnet model trained by the NL approach can be directly applied for different kinds of spectroscopy acquired by the same instrument. To confirm this, we have applied the same model to denoise the label-free Raman imaging of HeLa cells (Fig. 4e, the experimental parameters can be found in the figure caption). Raman images of two bands at the fingerprint region (1445 and 1612 cm^−^^1^: CH_2_ and CH_3_ deformation of lipids and proteins, 1656 cm^−1^: C = C stretching of lipids), and two bands at the CH abundant region (2867 cm^−1^: CH_2_ symmetrical stretching of lipids, 2915 cm^−^^1^: CH band of lipids and proteins)^49,50^ are presented. The raw images (Fig. 4e, left) of these bands are noisy and unanalyzable, albeit high laser power (c.a. 50 mW/line) and long integration time (5 s/line) is used during the data acquisition process. After pixel-wise noise removal with AUnet, the contrasts of these Raman images are much improved and the droplet-like distribution of intracellular lipids and proteins can be clearly observed. This indicates the potential application of NL approach for the downstream biological analysis, even possibly for real time analysis. The successful denoise of both fluorescence and Raman images obtained on the same instrument with the same AUnet model indicates that NL is indeed *instrument-dependent* rather than *sample-dependent*.

## Discussion

We report a NL approach to learn the characteristic noise distribution of Raman instruments from different companies, so that the deep learning model is trained using only the estimated instrumental noise together with a generated GT data without the need of laborious manual labelling. This allows a single AUnet model to be applicable to a variety of Raman spectra of different samples acquired by the instruments with different experimental conditions. This bypass the limitations of the conventional supervised machine learning techniques, whose performance heavily relies on the existing annotated dataset and only work well for “seen” samples. We have shown on multiple examples, from bulk silicon to 2D materials, and live-cells, that the AUnet trained by the NL approach can substantially improve the SNR of Raman spectra. Specifically, the SNR can be improved up to 10 folds and the MSE error can be reduced by 149 folds on dataset containing 12,500 Raman spectra of graphene.

Thanks to the enhanced SNR, the imaging speed of the Raman micro/nanoscopy has been significantly increased, and the required laser power has been decreased to ameliorate the potential photodamage to the sample. For instance, this technique allows the pixel-dwell time of TERS imaging to be reduced by at least 10 folds compared with the conventional setting^46^. All these improvements are achieved while the TERS signal still presents high SNR that faithfully reveal the atomic-scale electronic and catalytic properties of a bimetallic catalyst. Our method can also be extended to line-scan microscopy, for both the fluorescence and Raman imaging of live-cells using a single AUnet model trained by NL. This reduces the laser power by 43 folds for the fluorescence imaging, thereby facilitating long-term monitoring of the fragile biological samples with negligible photoinduced damage. Additionally, this method is also capable of denoising the photoluminescence imaging of 2D materials.

To conclude, NL has enhanced SNR in a wide range of spectroscopic scenarios, including nanoscopic imaging by TERS, and microscopic imaging by confocal Raman, fluorescence, and photoluminescence. Our proposed NL approach shifts the performance of deep learning from being *sample-dependent* to *instrument-dependent*. This holds potential for improving the performance of all the optical microscopy/spectroscopy modalities without requiring the hardware modifications via efficient computational approaches.

## Methods

### Optical measurements

Two optical microscope instruments were used in this work. LabRAM HR-Evolution (Horiba) were used for Raman measurements in Figs. 1–3 and Supplementary Figs. S4-5. Raman imaging of Cr/Si grating and graphene samples were acquired with air dry objective (100×, NA = 0.9) under 633 nm excitation, with laser power of 0.2 mW and 6 mW, respectively. An oil-immersion objective (60×, NA = 1.49) was used for the Raman mapping of HeLa cell, with 532 nm laser of 1.7 mW. An integration time of 100 ms/pixel was used for all those imaging experiments. TERS imaging were obtained with integrated SPM system under 633 nm excitation with a power of 0.25 mW and air-dry objective (100×, NA = 0.7). The pixel size is 2 nm in TERS imaging. A second instrument, Raman-11 (Nanophoton) line-scan microscope, were used to obtain the results in Fig. 4 and Supplementary Fig. S7-8. Fluorescence imaging of live-cells were obtained with 488 nm laser excitation and an integration time of 0.05 s/line (41 s/frame, air dry objective 50×, NA = 0.5). Raman images were acquired under 532 nm excitation with a laser power of 50 mW and integration time of 5 s/line (oil-immersion objective 60×, NA = 1.49). In our experiment of Raman measurement, the sampling density is 1.2 cm^−1^/pixel for LabRAM HR-Evolution (Horiba) and 4 cm^−1^/pixel for Raman-11 (Nanophoton) instruments.

### Physics-based GT Raman spectra

Due to interaction of molecules with its neighboring molecules and the various factors leading to the broadening of the peak, it is common knowledge that the line profile of Raman peak cannot be simply fitted with pure Gaussian or Lorentzian function. Instead, it is more appropriate to fit using a Voigt function, which is the convolution of Gaussian and Lorentz function. For simplicity, we used a pseudo version of the Voigt function to generate the Raman peaks:

1 $${{{{{\boldsymbol{V}}}}}}\left(x,\, \mu,\, w\right)=\rho \times {{{{{\boldsymbol{G}}}}}}\left(x,\, \mu,\, w\right)+(1-\rho )\times {{{{{\boldsymbol{L}}}}}}(x,\, \mu \,,w)$$

2 $${{{{{\boldsymbol{G}}}}}}\left(x,\, \mu,\, w\right)=\frac{1}{\sigma \sqrt{2\pi }}{e}^{-\frac{{(x-\mu )}^{2}}{2{\sigma }^{2}}},\, \sigma=\frac{w}{2\sqrt{2{{{{\mathrm{ln}}}}}2}}$$

3 $${{{{{\boldsymbol{L}}}}}}\left(x,\, \mu,\, w\right)=\frac{1}{\pi }\cdot \frac{w/2}{{(x-\mu )}^{2}+{(w/2)}^{2}}$$

where $\mu$ is the peak position, $w$ is the full width at half maxima (FWHM), ${{{{{\boldsymbol{G}}}}}}$ is the Gaussian part, ${{{{{\boldsymbol{L}}}}}}$ is the Lorentz part, $\rho$ represents the contribution of Gaussian function to the total Voigt function with $\rho=0.6785$ in this work. A Raman spectrum with multi-peak thus can be generated as follow:

4 $$R=\mathop{\sum }\limits_{i=1}^{N}S(i)=\mathop{\sum }\limits_{i=1}^{N}A(i)\times {{{{{\boldsymbol{V}}}}}}(x,U\left(i\right),W(i))$$

where $A$ denotes the amplitude of the signal, $U$ denotes the peak position, $W$ denotes the FWHM, $N$ denotes the total number of Raman peaks. Likewise, the baseline is also generated using the same method. These parameters are randomly extracted in a given range, which should be optimized on a specific Raman instrument. For example, the number of Raman peaks was set in 1-10 range, the FWHMs was set from 5 to 200 cm^−1^, and the peak position was set in the range of 1-1600 cm^−1^ when training the AUnet model for the Horiba instrument.

### Instrumental noise measurement

The instrumental noise in Raman microscopy is indirectly estimated from the spectra obtained from a smooth Au film, which does not exhibit observable Raman peaks (Fig. S1). Note, the Au film can still emit photoluminescence (PL) signals and reflect light, which need to be eliminated from the Au spectra. To this end, we propose to utilize singular value decomposition (SVD) to estimate these contributions from the measured Au spectra (Fig. S1). This allows us to extract the amplitude of the noise (i.e., fluctuation) from the original spectra, which is used for noise learning. The spectra are arranged as a two-dimensional matrix, which are then decomposed by the singular value decomposition (SVD) by:

5 $${S}_{m\times n}={U}_{m\times n}{\Sigma }_{m\times n}{V}_{m\times n}^{T}=	 \left[\begin{array}{ccc}{u}_{1} & \cdots & {u}_{m}\end{array}\right]\cdot \left[\begin{array}{cc}{\Sigma }_{k} & 0\\ 0 & 0\end{array}\right]\cdot \left[\begin{array}{c}{v}_{1}^{T}\\ \vdots \\ {v}_{n}^{T}\end{array}\right],\, \\ {\Sigma }_{k}=	 \left[\begin{array}{ccc}{\sigma }_{1} & \cdots & 0\\ \vdots & \ddots & \vdots \\ 0 & \cdots & {\sigma }_{k}\end{array}\right]$$

where $m$ denotes number of data point in each Raman spectrum determined by the effective readout pixels of the CCD detector, $n$ denotes the total number of the spectra in the matrix. After that, the instrumental noise can be estimated by removing the background components in the above equation. This can be done by manual inspection of each column vector of the left singular matrix ($$\begin{array}{ccc}{u}_{1} & \cdots & {u}_{m}\end{array}$$), or simply remove the first component (${\sigma }_{1}\bullet {u}_{1}{\bullet v}_{1}^{T}$) because it contributes the most majority of the background. By denoting $J$ as the set of background components index, the instrumental noise can be estimated as follows:

6 $${{{Noise}}_{m\times n}=S}_{m\times n}-\mathop{\sum}\limits_{J}{\sigma }_{j}\cdot {u}_{j} \, {\cdot \, v}_{j}^{T}$$

In this work, we measured 12,500 instrumental noise spectra on LabRAM HR-Evolution instrument. The measuring condition is: Laser wavelength, 633 nm; power, 0.1 mW; Integration time, 0.1~0.5 s/pixel (2500 spectra for each condition); Grating, 1800 gr/mm; 100× objective, NA = 0.7; Pinhole size: 100 μm. We measured 400,000 instrumental noise spectra in a line-scan manner (400 spectra/line) on Raman-11 instrument. The measuring condition is: Laser: 532 nm, 50 mW/line; Integration time: 0.05, 0.1, 0.5, 1 s/line (100,000 spectra for each condition); Grating: 600 gr/mm; Objective lens: 50×, NA = 0.45; Slit size: 50 μm.

### Noise learning, conventional supervised learning and network training

In the NL, the network is trained using the discrete cosine transform (DCT) coefficients data pairs of the noise spectrum and its corresponding instrumental noise. The output coefficient of the network is then processed by an inverse DCT (IDCT) operation to reconstruct the predicted instrumental noise. The DCT and IDCT can be described as follows:

7 $$Y\left(k\right)=\sqrt{\frac{2}{N}} * \mathop{\sum }\limits_{m=1}^{N}X\left(m\right)\cos \left(\frac{2m+1}{2N}k\pi \right)$$

8 $$X\left(m\right)=\sqrt{\frac{2}{N}} * \mathop{\sum }\limits_{k=1}^{N}Y\left(k\right)\cos \left(\frac{2k+1}{2N}m\pi \right)$$

The network is optimized with an MSE loss function, which can be described as:

9 $${loss}=\frac{1}{m}\mathop{\sum }\limits_{i=1}^{m}{\left|y\left(i\right)-x\left(i\right)\right|}^{2}$$

For each Raman instrument, 80% of the full dataset was used for training and 20% for validating. The stochastic gradient descent (SGD) was used as the optimizer, a cosine annealing learning rate strategy was used to tune the model. The model was trained 2000 epochs for LabRAM HR-Evolution, and 200 epochs for Raman-11. As for the conventional supervised learning model, the model architecture was the same. It was trained using the low- and high- SNR Raman spectra dataset. This dataset is generated using the method described in Supplementary Fig. S3. The original Raman spectra were acquired on a Cr/Si grating sample with 12,500 spectra in total. Experimental conditions are as follows: 633 nm laser, 0.1 mW; integration time was 0.1 s~0.5 s/pixel (2500 spectra for each condition). The model was trained 100 epochs for testing. The networks were implemented using PyTorch, and was trained on a graphic processing unit (GPU, Tesla V100, Nvidia).

### Traditional denoising algorithms

The Wavelet and SG filter used in this work are classical denoising methods, and we directly used the MATLAB version to implement the denoising. The function name for Wavelet in MATLAB is ‘wdenoise’, the denoising rule was ‘BlockJS’, and the wavelet decomposition level was 3. The function name of SG in MATLAB is ‘sgolayfilt’, the polynomial order was 2, the window was 15. The parameters for the two methods were manually selected after careful comparisons.

### Evaluation metrics

The MSE and SNR are used to evaluate the restoration of the spectra. The MSE is shown in Eq. (9), the SNR can be calculated as follows:

10 $${SNR}=10 * {\log }_{10}\frac{{x}^{2}}{{(y-x)}^{2}}$$

where $x$ denotes the GT spectrum, $y$ denotes the noisy spectrum. The PSNR and SSIM are used to evaluate the restoration of the images, and defined as follows:

11 $${PSNR}=10 * {\log }_{10}\frac{1}{{(Y-X)}^{2}}$$

12 $${SSIM}=\frac{\left(2{\mu }_{x}{\mu }_{y}+{c}_{1}\right)\left(2{\sigma }_{{xy}}+{c}_{2}\right)}{\left({\mu }_{x}^{2}+{\mu }_{y}^{2}+{c}_{1}\right)\left({\sigma }_{x}^{2}+{\sigma }_{y}^{2}+{c}_{2}\right)}$$

where $Y$ denotes the noisy image, $X$ denotes the reference image, ${\mu }_{x}$, ${\mu }_{y}$, ${\sigma }_{x}^{2}$, ${\sigma }_{y}^{2}$ and ${\sigma }_{{xy}}$ are the local averages, variances, and covariance for $X$ and $Y$, ${c}_{1}$ and ${c}_{2}$ are small constants that are used to stabilize the division with small denominator.

### Evaluation of spatial resolution of TERS

A common way to evaluate the spatial resolution of TERS is to track the Raman intensity change over a step^13^. In general, we use a sigmoidal function to fit the intensity curve by:

13 $$y={I}_{2}+\frac{{I}_{1}-{I}_{2}}{1+{e}^{-a(x-b)}}$$

Where ${I}_{1}$ and ${I}_{2}$ are the lower and upper limits, *a* is time constant and *b* is location of inflection point. Then, by taking the first derivative of the obtained sigmoid curve, we can obtain the bell-shaped curve, whose FWHM is a good evaluation of the TERS spatial resolution. To get statistically reliable result, we average the value of spatial resolution estimated from several parallel lines (Supplementary Fig. S12 and S13).

### Sample preparation

The ultra-flat Au films were fabricated by Hegner’s method^51^. In brief, 200 nm Au was first coated on Si(100) by vapor evaporation. Then, a glass slide was adhered to the Au film by high-temperature resistant epoxy glue. After lifting the glass slide, an atomically smooth Au film can be obtained. The Cr/Si grating sample was a magnification standard (684-1E, PelcotecTM CDMS-XY-1T) purchased from Ted Pella, Inc. (Sweden). It was made by depositing grating shaped 50 nm high Cr on an ultra-smooth silicon substrate.

Two-dimensional few layers of graphene and four kinds of transition metal dichalcogenides (TMDCs) were prepared by mechanical exfoliation method^52^. First, the Nitto tape was used to obtain fresh thick 2D flakes from bulk TMDC crystals. Then, the polydimethylsiloxane stamp (Gel-park, WF-20-X4 for TMDCs and WF-20-X8 for graphene) tore down monolayer or few layers TMDC by sticking to the thick flakes. Finally, the few layers graphene was transferred onto a SiO_2_/Si substrate and the few layers TMDCs were transferred onto an Au film. The thickness of graphene was determined by AFM and the thickness of TMDCs were determined by optical contrast and photoluminescence spectra. For obtaining large area samples suitable for focused ion beam (FIB) machining, we obtained millimeter-sized MoSe_2_ by Au film-assisted exfoliation method. The ultra-smooth Au film should be quickly attached to freshly cleaved MoSe_2_ flakes after being lifted from the Si(100) and heated at 100 °C for 10 min, to avoid the effect of surface impurities. When the MoSe_2_ flakes was uncovered, a large area of 2 L MoSe_2_ can be obtained. We next prepared the chessboard-like sample of MoSe_2_/Au by impacting MoSe_2_ with Ga^+^ ion beam. The width of the square lattice was set to 8 µm.

The HeLa cells were seeded onto 35 mm dishes containing coverslips and cultured in DMEM (Yuanpei, Shanghai, China) supplemented with 10% fetal bovine serum, 1% non-essential amino acids, penicillin, and streptomycin. The cells were incubated in a humidified 5% CO_2_ incubator at 37 °C for 24 h. For labeling, the adherent cells on the coverslip were stained with LysoTracker Green DND-26, MitoTracker Green FM, and BODIPY FL PI(5)P. The staining procedure followed the protocols provided by the manufacturer (Thermo Fisher Scientific, USA) and lasted for 30 min. After staining, the cells were washed twice with DMEM (without phenol red). For fluorescence imaging experiments, the stained cells were cultured in an in-situ pool (Tokai Hit) to maintain a humidified atmosphere (5% CO_2_, 95% air, 37 °C). For Raman imaging experiments, the cells were fixed in 75% ethanol for 10 min and then washed three times with sterile PBS. Subsequently, the cells were sealed on slides for Raman imaging.

The Pd/Au (111) sample was prepared by traditional electrochemical underpotential deposition (UPD) method. A well-defined Au(111) single crystal was made by Clavilier’s method and it was electrochemically polished and annealing with hydrogen flame before UPD. Sub-monolayer Pd was electrochemically deposited on Au(111) surface by one-drop solution method with the solution containing 1 mM H_2_PdCl_4_ and 0.1 M H_2_SO_4_. After that, the Pd/Au(111) was immersed in 20 µM phenyl isocyanide (PIC) ethanolic solution for 10 minutes to form self-assembled monolayer but avoiding surface etching. Au tips for TERS experiments were fabricated by reported electrochemical etching method^53^.

### Statistics and reproducibility

The code and datasets used for training and testing the deep-learning models are made publicly available for reproducibility. No statistical method was used to predetermine the sample size. No data were excluded from the analyses. In both simulated datasets, the training and testing datasets were randomly allocated.

### Reporting summary

Further information on research design is available in the Nature Portfolio Reporting Summary linked to this article.

### Supplementary information

Supplementary Information

Peer Review File

Description of Additional Supplementary Files

Supplementary Movie 1

Supplementary Movie 2

Supplementary Movie 3

Supplementary Movie 4

Reporting Summary

## Data Availability

All the data related to the work is available upon request to the corresponding author. Example Raman imaging datasets^54^ for neural network training and testing in this study have been deposited in the figshare under accession code 10.6084/m9.figshare.24823353.v1.

## Code availability

The code of AUnet can be download publicly^55^ at https://github.com/XMUSpecLab/Noise-learning. Other information is available upon request to the corresponding author.
