# Supplementary material for: Noise learning of instruments for high-contrast, high-resolution and fast hyperspectral microscopy and nanoscopy
Source: Nat Commun. 2024 Jan 25;15:754. doi: 10.1038/s41467-024-44864-5 (PMC10810791; doi:10.1038/s41467-024-44864-5)
Supplement: Supplementary file 1 — Supplementary Information [file 41467_2024_44864_MOESM1_ESM.pdf]

Supplementary Information for

Noise Learning of Instruments for High-contrast, High-resolution and Fast Hyperspectral Microscopy and Nanoscopy

Hao He[1,2,3], Maofeng Cao[2], Yun Gao[1], Peng Zheng[1], Sen Yan[2], Jin-Hui Zhong[4,†], Lei Wang[1,†], Dayong Jin[3,5], Bin Ren[2,6†]

[1] Pen-Tung Sah Institute of Micro-Nano Science and Technology, Xiamen, 361005, China.

[2]State Key Laboratory of Physical Chemistry of Solid Surfaces, Collaborative Innovation Center of Chemistry for Energy Materials (iChEM), The MOE Key Laboratory of Spectrochemical Analysis and Instrumentation, College of Chemistry and Chemical Engineering, Xiamen University, Xiamen 361005, China.

[3]Department of Biomedical Engineering, College of Engineering, Southern University of Science and Technology, Shenzhen 518055, Guangdong, China.

[4]Department of Materials Science and Engineering, Southern University of Science and Technology, Shenzhen 518055, China.

[5]University of Technology Sydney, Sydney, NSW 2007, Australia.

[6] Tan Kah Kee Innovation Laboratory, Xiamen 361104, China

[†] Correspondence: J.-H.Z. (zhongjh@sustech.edu.cn); L.W. (wanglei33@xmu.edu.cn); B.R. (bren@xmu.edu.cn).

Table of Contents

Supplementary Figures 3

Fig. S1 | Scheme of measuring and analyzing the instrumental noise of Raman microscopes. 3

Fig. S2 | The practical meaning of Fourier transform (FT) of the spectrum 4

Fig. S3 | Principle of the simulation method to obtain paired spectra of low and high signal to noise ratio 5

Fig. S4 | Other results demonstrating the effectiveness of different methods to remove the instrumental noise from Raman spectra of 2D materials 6

Fig. S5 | Performance of NL method at the low laser power limit 7

Fig. S6 | The scheme of Horiba LabRAM HR-Evolution system that integrates confocal Raman with tip-enhanced Raman spectroscopy working in a back-scattering configuration 8

Fig. S7 | Photobleaching of the fluorophores excited with high laser power 9

Fig. S8 | Additional results on living-cell fluorescence imaging 10

Fig. S9 | AUnet assisted fast line-scan photoluminescence imaging of 2D materials on gold substrate 11

Fig. S10 | Simulated Raman imaging data with a horizontal edge structure. 12

Fig. S11 | Calculation of spatial resolution of the simulated line profiles. 13

Fig. S12 | Spatial resolution analysis of the noisy and AUnet-processed TERS image. 14

Fig. S13 | Another two step edge regions for the analysis of the spatial resolution 15

Supplementary Discussion 16

1. Noise type analysis 16

Fig. S14 | Noise analysis of Si and Au 17

Fig. S15 | Standard deviation analysis of Au with different laser power 19

2. Positioning accuracy of noisy and AUnet-denoised image 19

3. Spectral resolution of noisy and AUnet-denoised spectra 20

Fig. S16 | Classical Rayleigh criterion for resolving two peaks in a Raman spectrum. 21

Fig. S17 | Comparison of the probability to resolve two close peaks 22

Supplementary references 23

Supplementary Figures

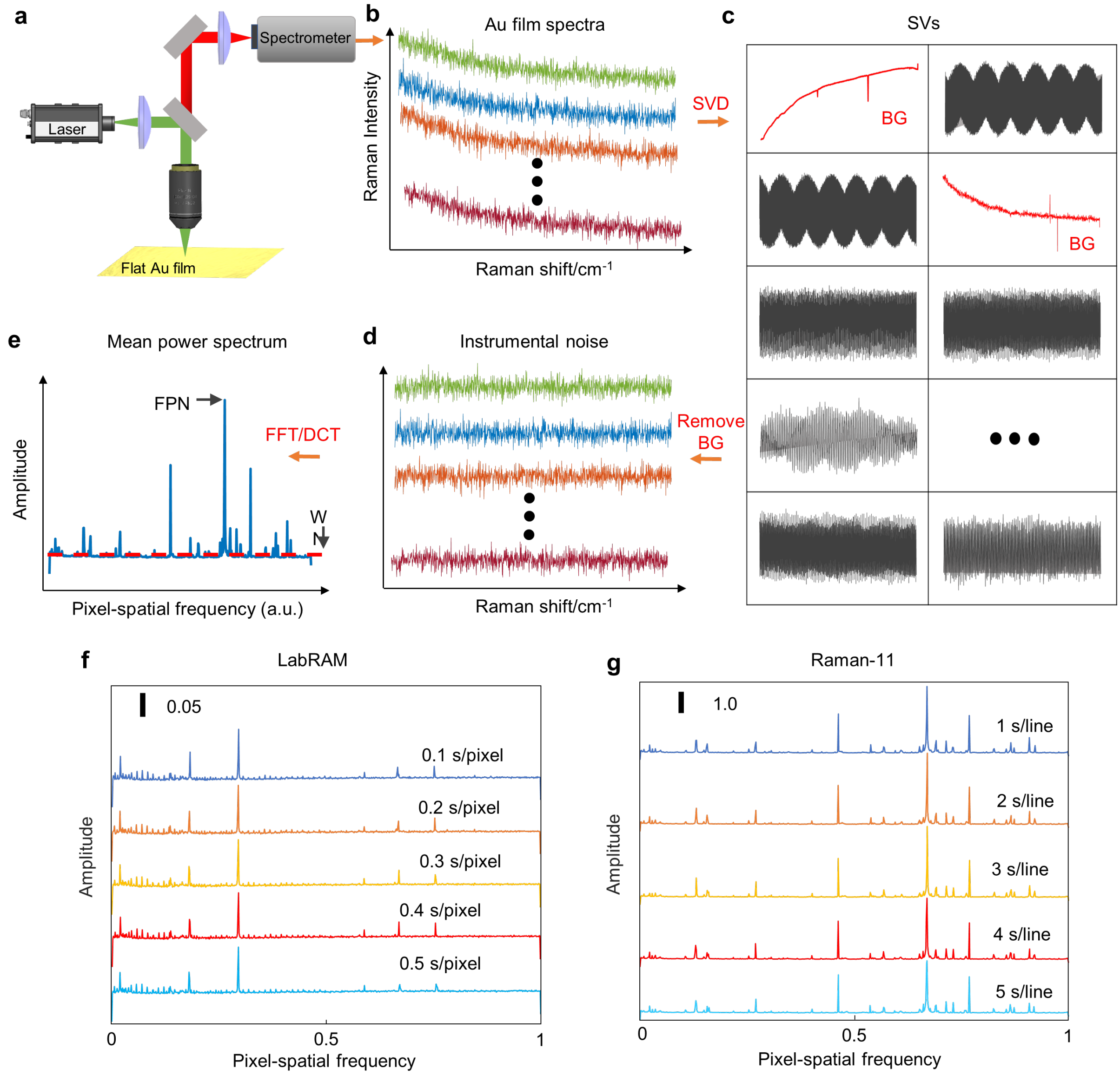

Fig. S1 | Scheme of measuring and analyzing the instrumental noise of Raman microscopes. (a) A scheme of the set-up for measuring instrumental noise from a smooth Au film. **(b)** The noise spectra from Au film. **(c)** The singular values (SVs) of two-dimensional (2D) matrix of noise spectra, which is obtained by singular value decomposition (SVD, see 'Methods - Instrumental noise measurement' section in the main text for more details). The SVs related to background (BG, red) are removed, and the remaining SVs (black) are summed to obtain the instrumental noise spectra. **(d)** The reconstructed instrumental noise spectra. **(e)** Transform each instrumental noise spectrum either with fast Fourier transform (FFT) or discrete cosine transform (DCT) to obtain the mean power spectrum of each instrument in the frequency domain. The power spectrum shows a clear pattern, which can be further separated into white noise (WN, i.e., the baseline of the power spectrum) and fixed pattern noise (FPN, i.e., the noise source at a specific frequency). **(f)** The power spectra of the instrumental noise of LabRAM HR-Evolution at different acquisition time (0.1 s-0.5 s). For each acquisition time, 2,500 instrumental noise spectra were acquired. Other experimental conditions: 633 nm laser (0.1 mW on sample), air dry objective (NA=0.9, 100×); **(g)** The power spectra of the

instrumental noise of the Raman-11 at different acquisition time. For each acquisition time, 10,000 instrumental noise spectra were acquired in a line-scan manner (400 pixels/line). Other experimental conditions: 532 nm laser (50 mW on sample); long-working distance air dry objective (NA=0.45, 50×). The results of two commercial Raman instruments clearly demonstrate that each instrument has a stable, instrumental-specific noise pattern in the frequency domain, which could be statistically modelled using deep learning method.

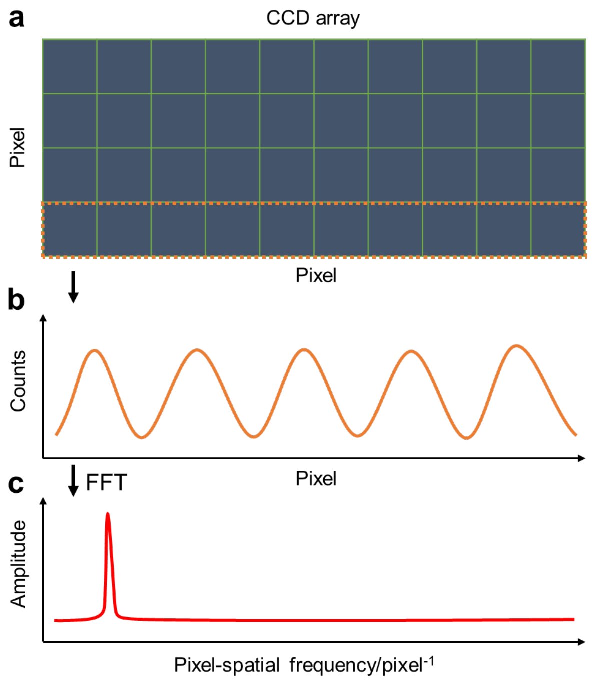

Fig. S2 | The practical meaning of Fourier transform (FT) of the spectrum. (a) The scheme of CCD array that is used as the detector to record the signals in the Raman instruments. (b) The spectrum recorded by the pixel row of the CCD array marked by the dotted rectangle in (a). (c) The fast FT (FFT) transform of the spectrum. Here the horizontal axis corresponds to the pixel-spatial frequency.

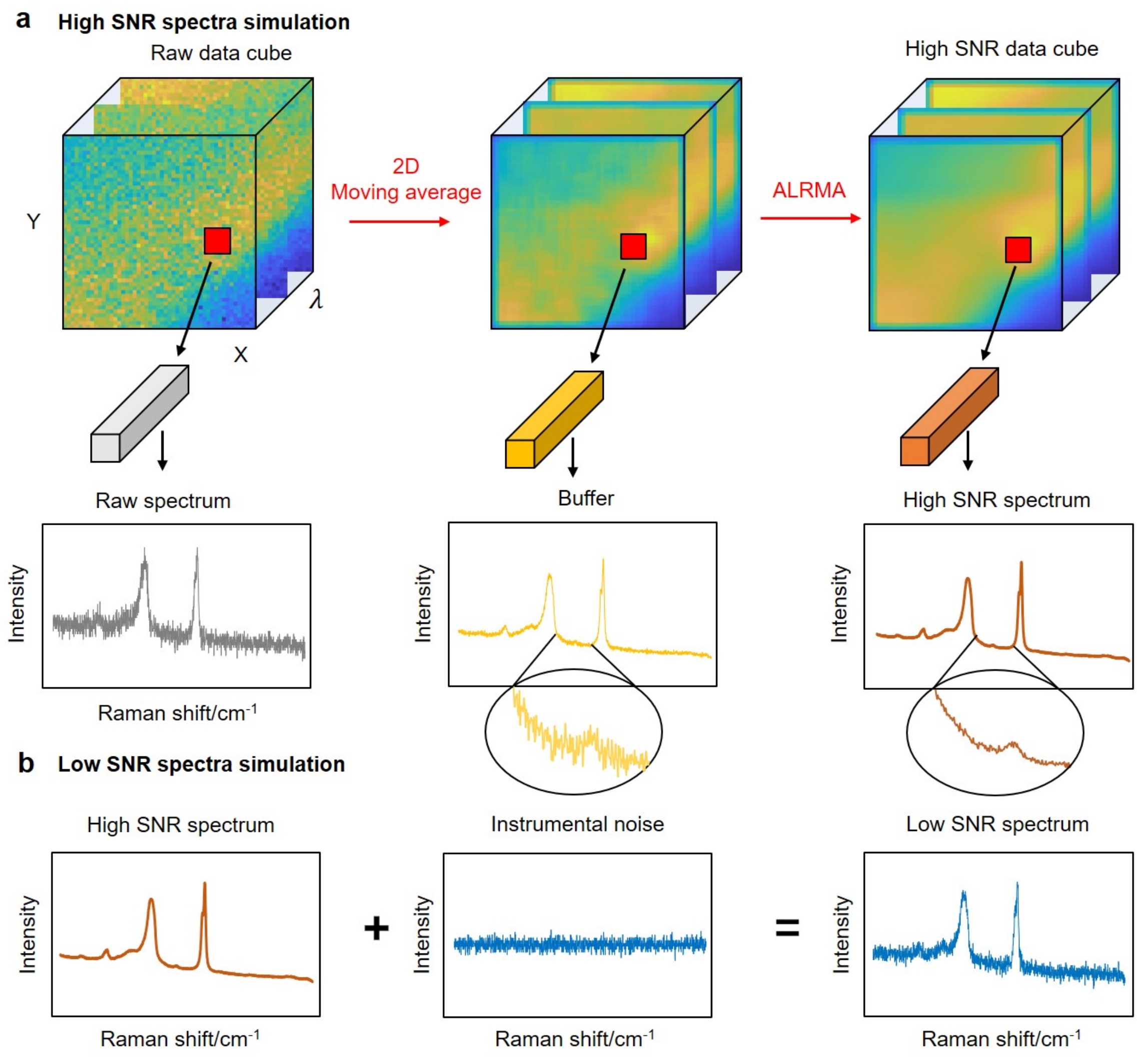

Fig. S3 | Principle of the simulation method to obtain paired spectra of low and high signal to noise ratio (SNR). (a) Two steps to simulate the high-SNR Raman spectra using the raw hyperspectral Raman imaging data acquired from real sample: (1) The raw imaging data are stacked as a three-dimensional (3D) data cube, which are filtered with 2D moving average (with 11×11 window) for each image slice. This step suppresses part of the noise while retains the fidelity at the spectral dimension, which can be observed from the raw (gray, left) and buffer (yellow, middle) Raman spectra; (2) An adaptive low-rank matrix approximation (ALRMA) [1,2] method is used to further suppress the noise and produce the ground truth (GT) data with high SNR. The inset shows the zoom-in spectra of the buffer spectra (yellow) and ALRMA-processed high-SNR spectra (brown) for comparison. **(b)** Simulated low SNR spectrum obtained by directly adding the instrumental noise to the high SNR one. The instrumental noise is extracted using the method described in Supplementary Fig. S1.

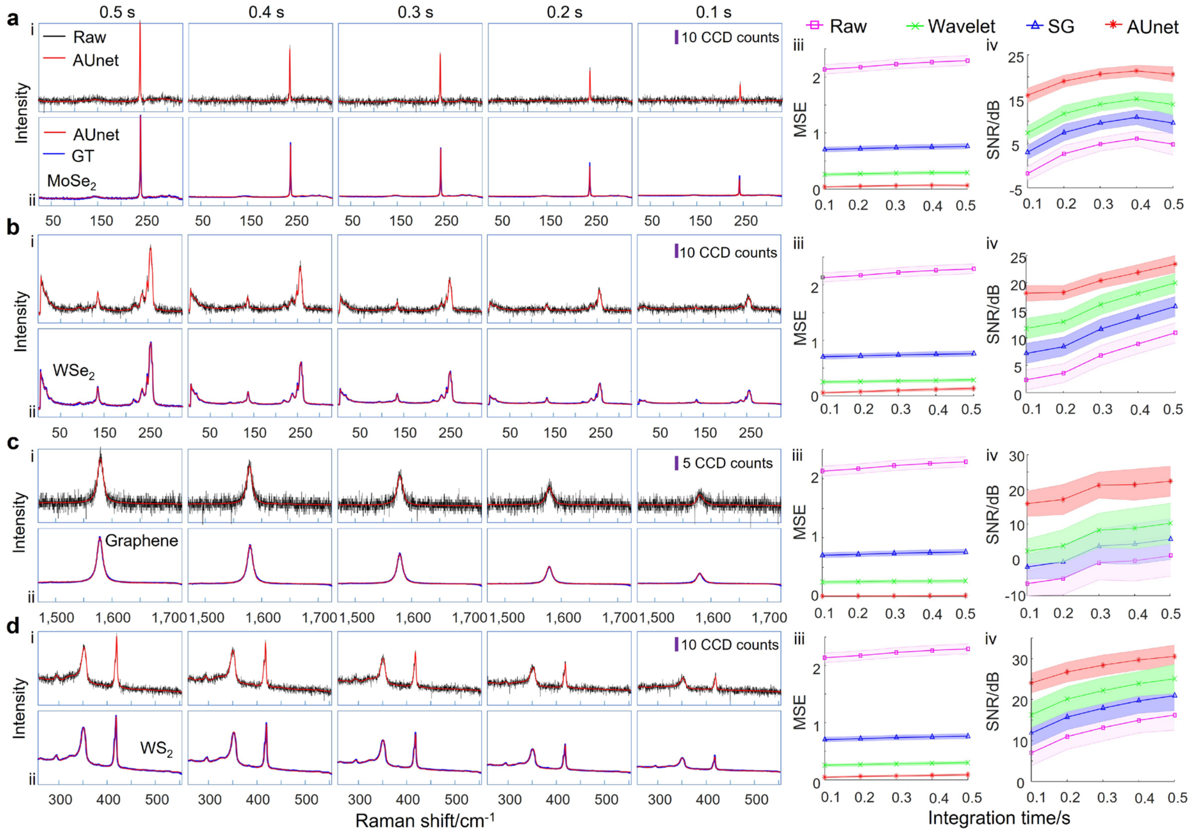

Fig. S4 | Other results demonstrating the effectiveness of different methods to remove the instrumental noise from Raman spectra of 2D materials. Results of (a) $MoSe_2$, (b) WSe_2, (c) graphene and (d) WS_2 obtained at different acquisition time of 0.1 s to 0.5 s. For each sample, panel i shows the comparison of the raw (black) and AUnet (red) processed Raman spectra; panel ii compares the ground true spectra (GT, blue) with the AUnet processed spectra (red); panels iii and iv present, respectively, the mean square error (MSE) and the SNR of the raw data and the spectra processed with AUnet, Wavelet and SG (see Methods in the main text). For each acquisition time, the MSE and SNR values were the average of 2,500 spectra, and the shaded areas indicate the deviation. The AUnet model used here is the same as Fig. 1e and Fig. 2 in the main text. These results demonstrate the generality of the AUnet model trained using the noise learning (NL) method and its state-of-the-art denoising performance. Measuring conditions: Instrument, LabRAM HR Evolution; laser wavelength, 633 nm; laser power, 6 mW for Graphene, 0.25 mW for WS_2, and 1.25 mW for $MoSe_2$ and WSe_2; Objective, 100× air dry (NA=0.9), grating: 1800 l/mm.

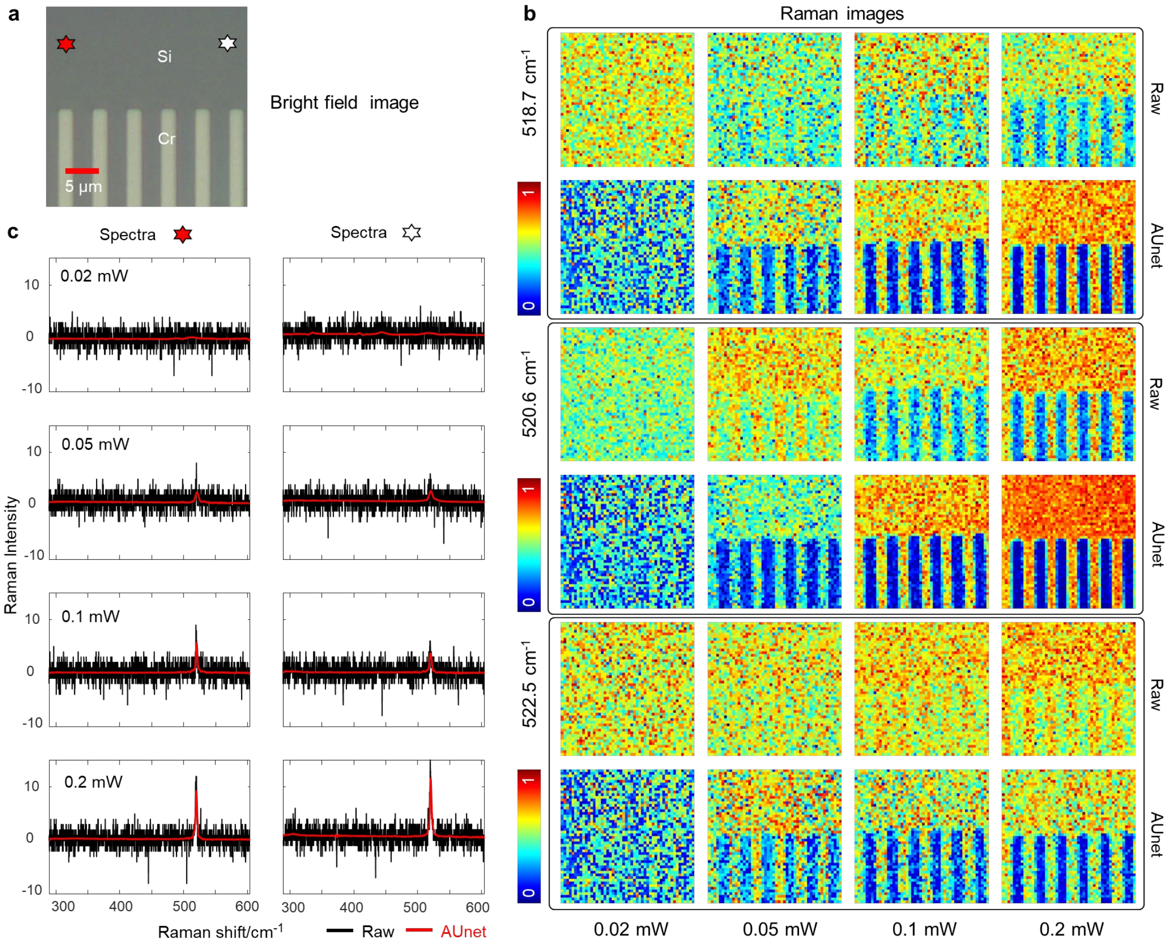

Fig. S5 | Performance of NL method at the low laser power limit. (a) Bright field image of a Cr/Si grating sample. **(b)** Raman images at three typical wavenumbers (518.7, 520.6, 522.5 cm^{-1}) of the sample acquired with different laser power (0.02, 0.05, 0.1 and 0.2 mW). For each panel, the first row illustrates the raw images, and the second row illustrates the AUnet restored ones. **(c)** The raw and AUnet restored Raman spectra extracted at the position marked by the red and white stars on the bright field image. Instrument: LabRAM HR-Evolution (Horiba). The AUnet model used here is the same as Fig. 1e and Fig. 2 in the main text without retraining. Objective: air dry, 100×, NA = 0.9. Laser source: 633 nm. Integration time: 100 ms/point. Grating: 1800 l/mm. The Raman signal of Si gradually decreases with decreasing laser power, and it almost disappears at 0.05 mW in the raw data. Such a weak signal can still be differentiated using AUnet. This can be confirmed from the Raman images in (b). The image structures are totally buried in the noise in the raw data at 0.05 mW laser power, especially the 522.5 cm^{-1} peak, while AUnet clearly uncovers such structures that agree well with the bright field image. This result demonstrates that the NL can push the limits of Raman microscopy.

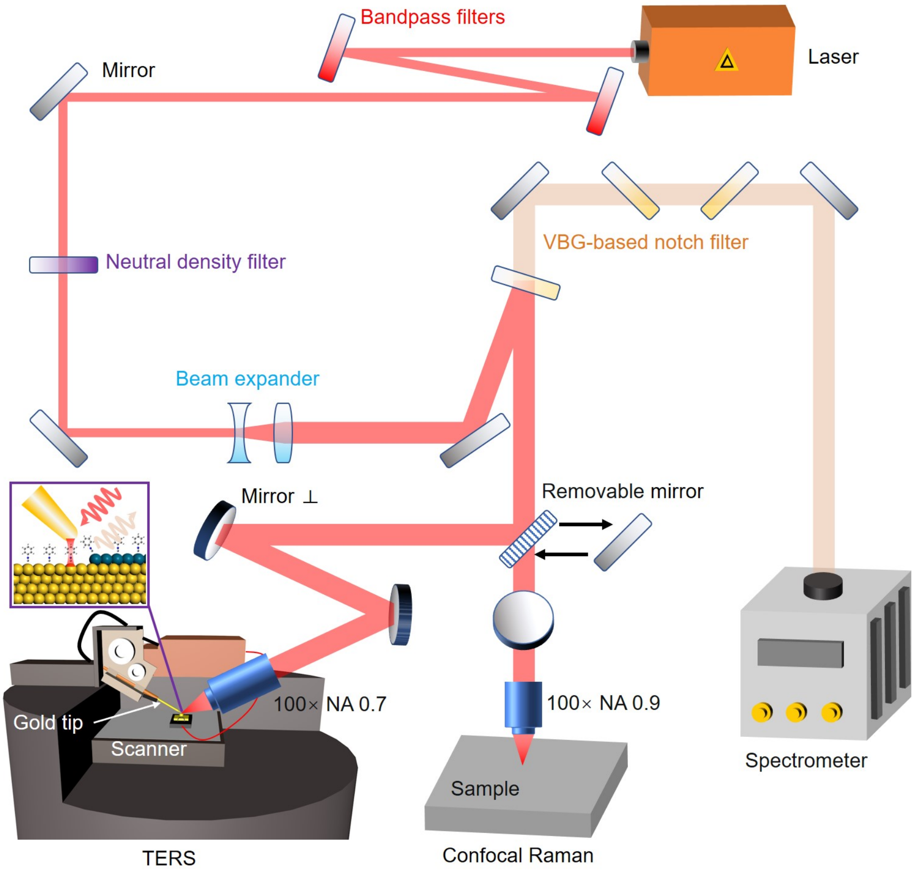

Fig. S6 | The scheme of Horiba LabRAM HR-Evolution system that integrates confocal Raman with tip-enhanced Raman spectroscopy working in a back-scattering configuration. For STM-TERS, the laser is irradiated at the plasmonic tip-sample junction at 25° relative to the horizontal plane (diagram in the purple box is the structure of sample). The switch of the two modalities is achieved via the removable mirror. To get ultra-low frequency Raman signal (< 50 cm^{-1}), several volume Bragg grating (VBG) based notch filters were used to weaken Rayleigh line and additional bandpass filters were used to remove plasma lines from laser source.

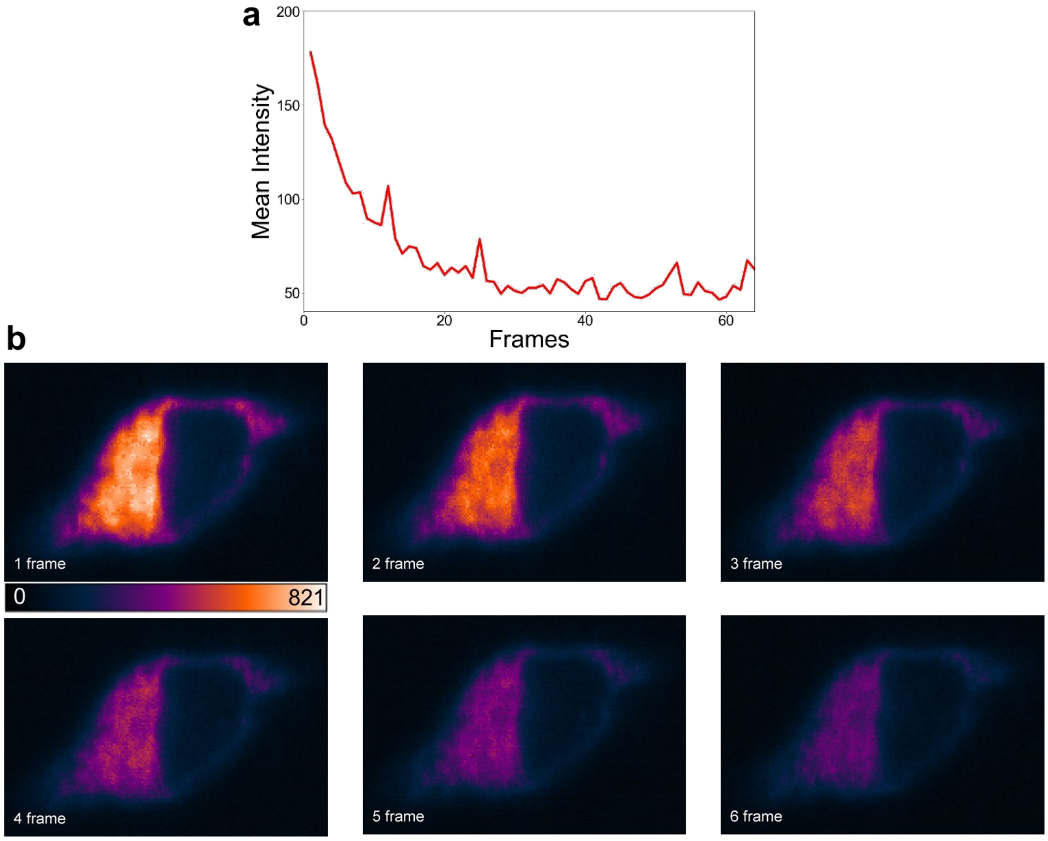

Fig. S7 | Photobleaching of the fluorophores excited with high laser power. (a) The mean fluorescence intensity curve of the HeLa cell. (b) The first 6 frames of fluorescence imaging of HeLa cell with high laser power. The intensity scale for each frame is the same to allow a better comparison of the photobleaching behavior.

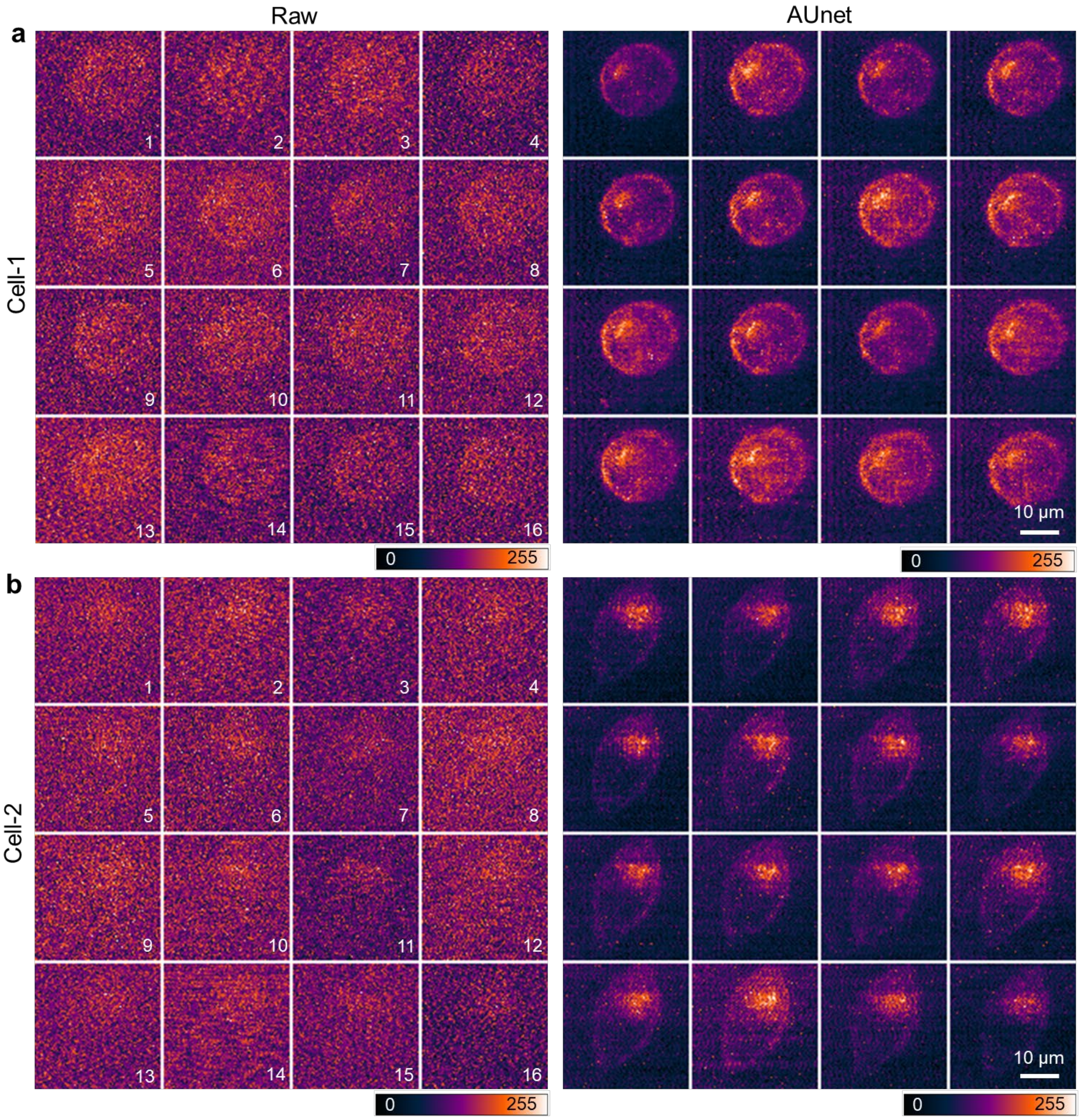

Fig. S8 | Additional results on living-cell fluorescence imaging. Continuous fluorescence imaging of **(a)** a round-shape HeLa cell and **(b)** spinel-shape Hela cell using the fluorescence signal at 501 nm from BODIPY FL PI(5)P targeting lysosome. Left: raw images with the number of frames indicated; Right: images after AUnet treatment. Instrument: Raman-11; Objective: air dry 50×, NA = 0.45; Laser wavelength: 488 nm; laser power: 30 μW; integration time: 50 ms/line and 35 s/frame; grating: 600 l/mm.

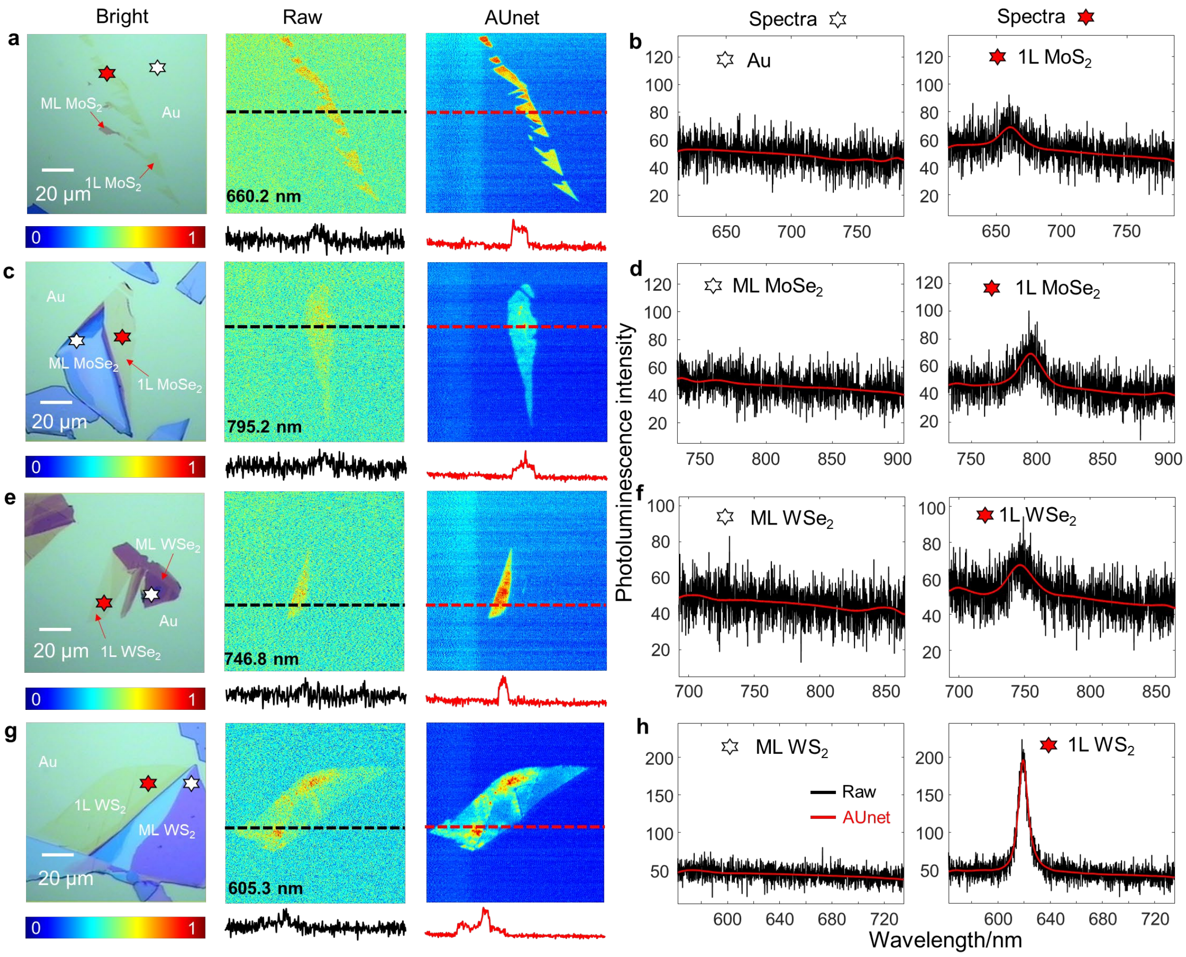

Fig. S9 | AUnet assisted fast line-scan photoluminescence imaging of 2D materials on gold substrate. (a) MoS_2, (c) $MoSe_2$, (e) WSe_2, (g) WS_2. Left: bright field image. Middle: raw fluorescence image at the indicated wavelength. The black curve at the bottom is the intensity profile along the black dashed line on the image. Right: fluorescence image after pixel-wise denoising by the pretrained AUnet, the red curve at the bottom is the intensity profile along the red dashed line. **(b, d, f, h)** Corresponding spectra of the four samples. Left: fluorescence spectra at the position marked by the white and red stars on the bright field image. Black curves indicate the noisy raw spectra and red curves are the high SNR spectra processed by AUnet. The AUnet model used here is the same as Fig. 4 in the main text without retraining. Measurement conditions: Instrument: Raman-11, Nanophoton; Laser wavelength, 532 nm; Laser power, 0.08 mW; Objective: 50× air dry (NA=0.45); Integration time: 100 ms/line; Grating: 600 l/mm.

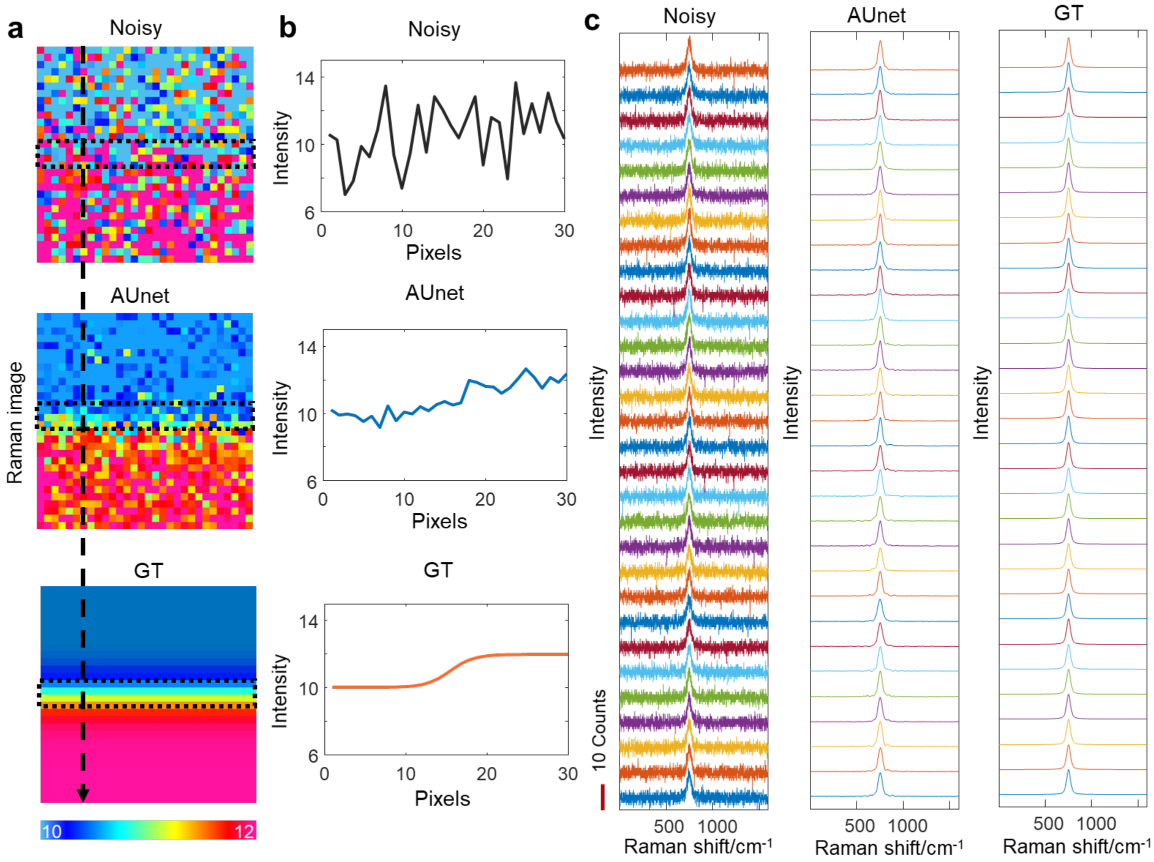

Fig. S10 | Simulated Raman imaging data with a horizontal edge structure. (a) Simulated Raman image (using the peak intensity at 750 cm^{-1}) with a horizontal edge structure marked by the dashed rectangles. From top to bottom panel are Noisy, AUnet-processed and GT Raman image with 30×30 pixels. In this typical example, the intensity at the bottom of the edge is 10, and at the top is 12. (b) The Raman peak intensity line profile along the black dashed vertical line marked on the Raman images in (a). From top to bottom panel are Noisy, AUnet-processed and GT line profiles with 30 pixels. (c) The Raman spectra along the black dashed line marked on the Raman images in (a). From left to right are Noisy, AUnet-processed and GT Raman spectra.

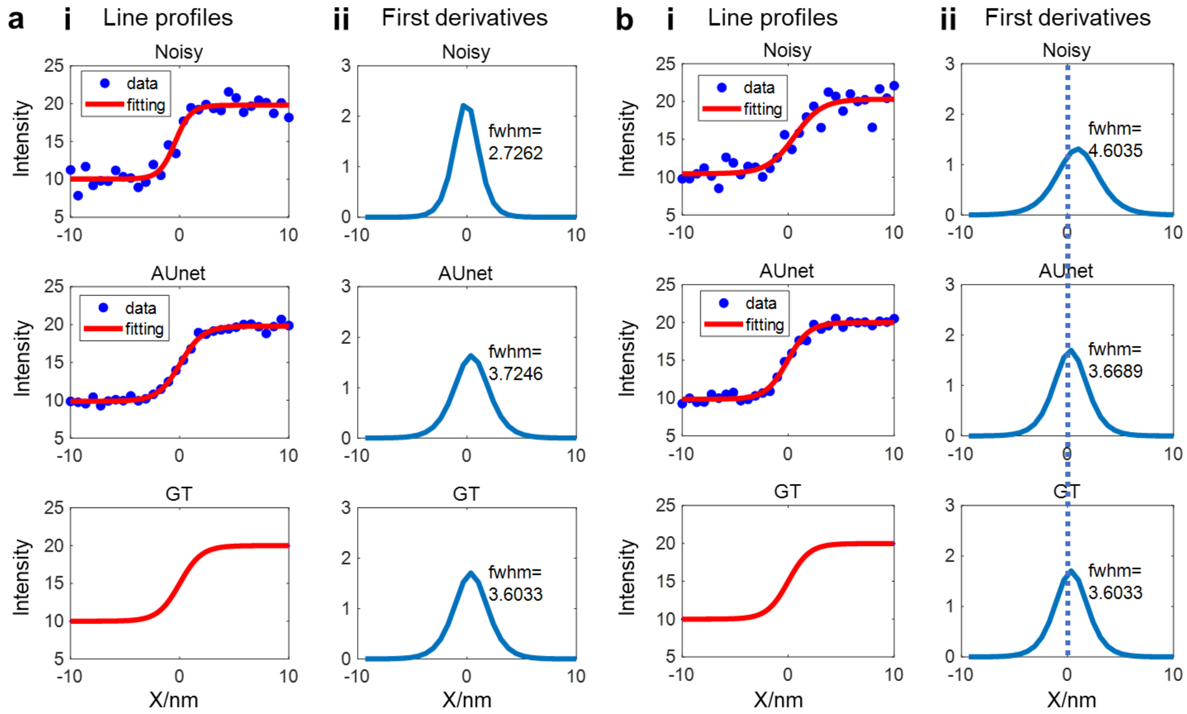

Fig. S11 | Calculation of spatial resolution of the simulated line profiles. (a-i) Simulated line profiles of peak intensity of the Raman spectra (using the method described in Fig. S10), and fitting results using Eq. (S1). From the top to bottom panel are Noisy, AUnet-processed and GT results, respectively. In this typical example, the bottom intensity of the edge is 10, and the top is 20. (a-ii) The first derivative curves corresponding to the red curves of (a-i), and the calculated FWHMs. (b) Another case similar to (a). The result shows that AUnet-denoising leads to a spatial resolution much closer to the value of GT and a better positioning accuracy compared to the result of the noisy data.

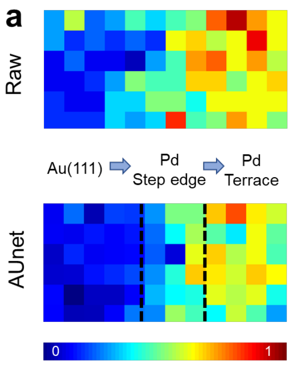

b

	Raw		AUnet	
Row number	R-squared	FWHM (nm)	R-squared	FWHM (nm)
1	0.90	6.8	0.93	6.9
2	0.99	2.1	0.96	3.8
3	0.96	3.9	0.94	8.5
4	0.95	10.1	0.99	2.5
5	0.96	10.3	0.94	8.8
6	0.95	11.1	0.97	5.4
Mean ± SD		7.4±3.7		6±2.5

Fig. S12 | Spatial resolution analysis of the noisy and AUnet-processed TERS image. (a) Zoom-in images of the white arrow region in Fig. 3c in the main text. The AUnet-processed image shows much clearer distinction between the left (Au) and right (Pd covered Au) regions, whereas the intensity distribution in the noisy (raw) image is much more spread. (b) Fitted results using the derivative of Eq. (S1) for the 13 horizontal lines in the image shown in (a). The results indicate that the AUnet denoising can improve the spatial resolution (6 vs 7.4 nm) and reduce the standard deviation (2.5 nm vs 3.7 nm) of the estimated spatial resolution.

a

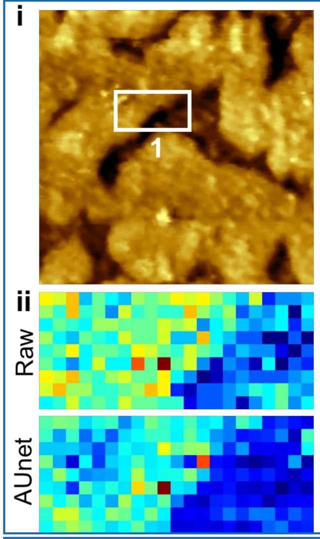

iii

	Raw		AUnet	
Row number	R-squared	FWHM (nm)	R-squared	FWHM (nm)
1	0.61	3.7	0.88	3.3
2	0.65	5.4	0.71	4.6
3	0.58	N/A	0.72	4.3
4	0.67	7.6	0.88	4.8
5	0.92	9.3	0.89	2.7
6	0.02	N/A	0.84	3.4
7	0.87	4.8	0.94	4.0
8	0.68	2.4	0.87	4.3
9	0.05	N/A	0.90	2.9
Mean± SD		5.5 ± 2.5		3.8 ± 0.76

b

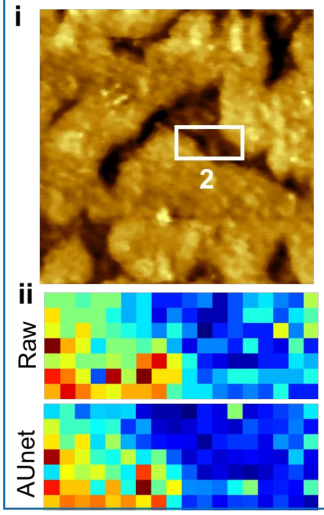

iii

	Raw		AUnet	
Row number	R-squared	FWHM (nm)	R-squared	FWHM (nm)
1	0.96	7.4	0.98	4.6
2	0.82	2.2	0.81	N/A
3	0.66	12.7	0.93	3.5
4	0.96	N/A	0.93	N/A
5	0.02	N/A	0.89	5.5
6	0	N/A	0.96	4.8
7	0.70	14.7	N/A	N/A
Mean± SD		9.3±5.6		4.6±0.8

Fig. S13 | Another two step edge regions for the analysis of the spatial resolution. (a) Spatial resolution analysis within the region 1. (b) Spatial resolution analysis within the region 2. In each panel, (i) shows the STM image with marked region, (ii) plots the raw (top) and AUnet processed (bottom) TERS image, and (iii) is the spatial resolution result for each row and their average value.

Supplementary Discussion

1. Noise type analysis

The noise of the measured Raman spectra includes signal-dependent shot noise and instrument-dependent noise. While the instrumental noise remains relatively stable, the signal shot noise is proportional to the square root of the photon number. Therefore, with the reduced signal photon number, the weight of the signal shot noise will decrease and the weight of the instrumental shot noise will increase. Predictably, the instrumental noise is the dominant noise source when the signal photon number is sufficiently small. To validate this, we measured a batch of Si and Au spectra, and analyzed their signal shot noise. The average spectra of Au and Si are shown in **Fig. S14(a)**. The measured signal noise variance of Au and Si spectra are composed of several parts and can be written as Eq. (S1) and Eq. (S2), respectively.

$$N^2(\text{Au}) = SN^2{}_{pl} + SN^2{}_{bg} + N^2{}_{ccd} \tag{S1}$$

$$N^2(\text{Si}) = SN^2{}_{signal} + SN^2{}_{bg} + N^2{}_{ccd} \tag{S2}$$

Here *SN* means shot noise (Poisson distribution) and *N* means other noise (Gaussian, Tukey-Lambda and uniform distribution)[3,4]. SN_{bg} denotes the shot noise arising from the stray light caused by the laser, N_{ccd} is the noise of the CCD detector, SN_{signal} denotes the shot noise of the Raman signal, SN_{pl} denotes the shot noise of the photoluminescence signal. Among them, SN_{bg}, SN_{pl} and SN_{signal} also contain the contribution from laser fluctuation. The instrumental shot noise includes SN_{bg} and N_{ccd}.

The detail steps for analyzing the shot noise are as follow:

1. We first measured the spectra of Au and Si substrate on the same position for 100 times in the line scan mode of Raman-11 instrument. Each laser line generates 400 spectra. The total number of spectra is thus 40,000.
2. The averaged spectrum of all these measured spectra is then taken as the low noise background.
3. The overall shot noise can be obtained by subtracting the background from the measured spectra.
4. The shot noise is taken as the standard deviation (σ) of the noise spectra using Eq. (S3).

$$\sigma = \sqrt{\frac{1}{N-1}\sum_{i=1}^{N}\sum_{j=1}^{M}(x_{i,j} - \mu_j)^2} \;;\; \mu_j = \frac{1}{N}\sum_{i=1}^{N} x_{i,j} \tag{S3}$$

where i is the measurement number, N is total number of measurements, j is the wavenumber index, M is total number of wavenumbers, μ_j denotes the mean intensity at j-th wavenumber of all the measurements.

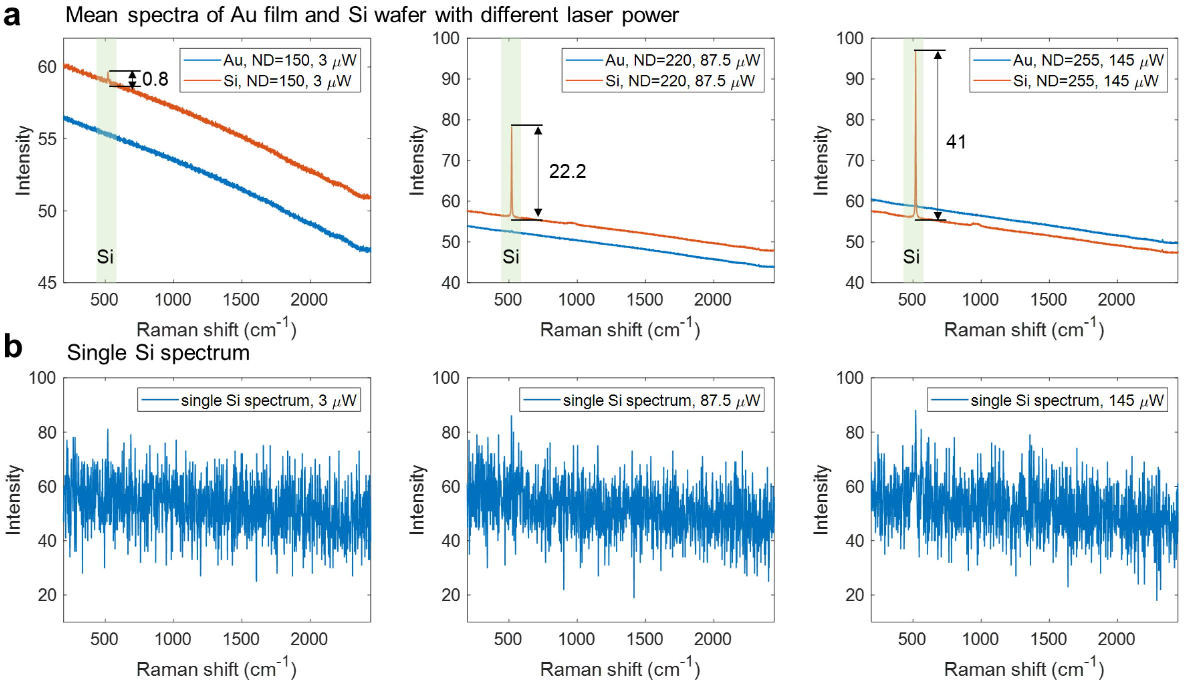

c Standard deviation analysis

	3 µW/pixel		87.5 µW/pixel		145 µW/pixel	
	Au	Si	Au	Si	Au	Si
Si band	8.81	8.93	8.87	9.05	8.91	9.13
Overall Exclude Si band	8.82	8.92	8.86	8.90	8.87	8.90

Fig. S14 | Noise analysis of Si and Au. (a) Mean spectra of the Au and Si measured with different laser power by Raman-11 using line-scan mode at the same positoin for 100 times repetive measurements, each laser line consists of 400 spectra. Laser = 532 nm, 50× air dry objective (NA=0.45), integration time: 0.5 s/line.These spectra are taken as the background and subtracted from the raw spectra to estimate the noise. (b) Single Si spectrum acquired with different laser power in line-scan mode. (c) The standard deviation calculated for the spectra region within the Si Raman band highlighted by light green in (a), and the overall spectral range excluding the Si band.

We first calculated the noise of Si and Au spectra within the Si Raman band region highlighted by the light green background as shown in **Fig. S14(a)**. The standard deviation of Si and Au spectra are quite similar (8.8~8.9 CCD counts) within the Si Raman signal band under weak laser power (3 µW/pixel in line-scan mode) condition. Interestingly, the standard deviation in the overall spectral range is also the same as that of the spectral region of the Si Raman band (**Fig. S14 (b)**). We further increased the laser power (87.5 µW/pixel and 145 µW/pixel in line-scan mode) and used the same method to calculate the shot noise to see whether the increased Raman signal brings significant change or not. The results show that the standard deviation of Si increased slightly by ~0.2 CCD counts. Although the maxima Raman signal intensity of the Si obviously increased from 0.8 to 41 (**Fig. S14 (a)**), the standard deviation of the Si band remains stable compared with that of the full spectral range (8.9 vs 9.13, **Fig. S14 (b)**). In view of these observations, we confirm that the shot noise originating from the Raman signal is low compared to the instrumental shot noise and can be neglected in weak signal conditions of our applications.

To further quantify the instrument noise, we also measured the Au film with different laser power (**Fig. S15**), and calculated the corresponding standard deviation with the above method. Interestingly, the standard deviation is 8.84 in the absence of laser irradiation (0 µW, $SN_{pl} = 0$), meaning the CCD and background serve as the primary noise source in this scenario (**Fig. S15a**). It only slightly increased to 9.02 even with a laser power of 145 µW. Also, the noise fluctuation range seems stable with different laser power as shown in **Fig. S15(b)**. We can try to estimate the relative contribution of different noise sources.

When the laser power is 0, the slit of the spectrometer is closed, ($SN^2{}_{pl} = 0, SN^2{}_{bg} = 0, \sigma = 8.84$, **Fig. S15a**), we have:

$$N^2{}_{ccd} = 8.84^2 = 78.15 \tag{S4}$$

When the laser power is 145 µW ($\sigma = 9.02$, **Fig. S15a**) and suppose N_{ccd} remains stable, we have:

$$SN^2{}_{pl} + SN^2{}_{bg} + N^2{}_{ccd} = 9.02^2 = 81.36 \tag{S5}$$

Combining Eq. (S4) and (S5) we have:

$$SN^2{}_{pl} + SN^2{}_{bg} = 3.21 \tag{S6}$$

Since $SN_{pl} \geq 0$, $SN_{bg} \geq 0$, we can estimate that:

$$SN^2{}_{bg} < 3.21,\ \ SN^2{}_{pl} < 3.21 \tag{S7}$$

Therefore, we can deduce that in the measured Au spectra with 145 µW laser power, the CCD contributes to the majority (~96%) of the noise variance, and the noise variance coming from the background and PL signal make minor contribution (~4%).

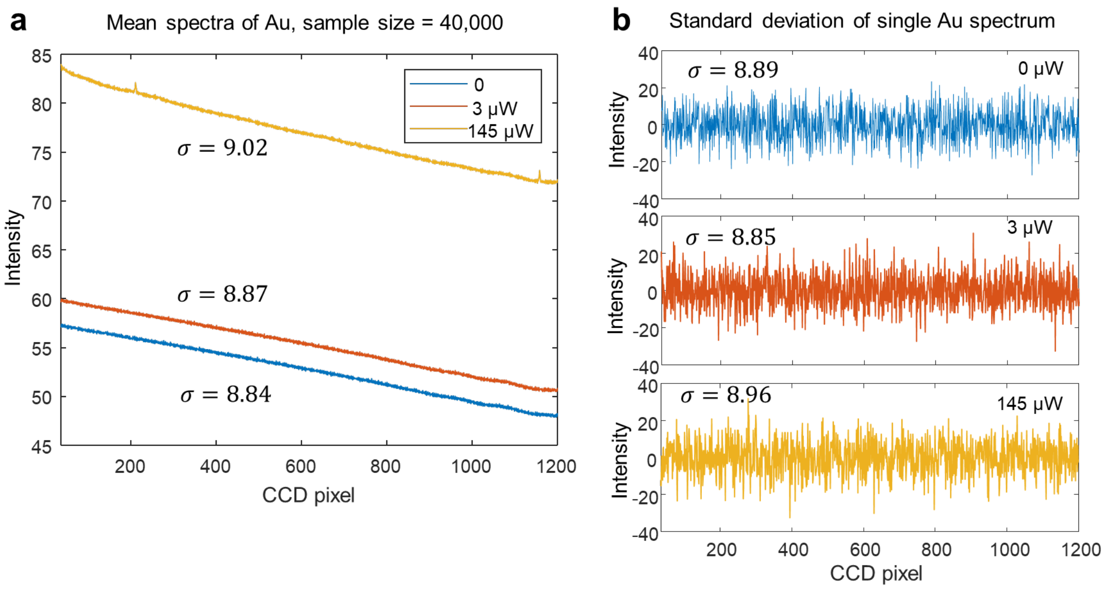

Fig. S15 | Standard deviation analysis of Au with different laser power. (a) Mean Au spectra and standard deviation measured by Raman-11 using line-scan mode at the same positoin for 100 times repetive measurement with different laser power, each laser line consists of 400 spectra. Laser = 532 nm, 50× air dry objective (NA=0.45), integration time: 0.5 s/line. (b) Representative shot noise spectra of Au measured with different laser power. Note that the increase in the baseline intensity is not from the photoluminescence of Au but due to the change/fluctuation of the offset bias of CCD, as confirmed by more repetive measurements and comparing to the data in Fig. S14 for the same laser power.

2. Positioning accuracy of noisy and AUnet-denoised image

The enhanced SNR from the denoising process can substantially improve the positioning accuracy of Raman imaging. This can be observed in **Fig. S11-c, d**. As indicated by the vertical dotted line (**Fig. S11-d**), the peak position of the noisy spectrum deviates from the GT data, whereas the AUnet-processed data aligns perfectly with the GT. More importantly, from the two cases shown in **Fig. S11**, we see that the FWHM of the AUnet-processed data is much closer to that of the GT data, whereas the noisy spectra can either show increased or decreased FWHM compared to that of GT. To statistically quantify this observation, we generated a series of edge structure with different parameters (I_1, I_2, a, b) (Eq. (S8)). Here for simplicity, we set $a = 1$, $b = 0$, and $I_2 = 10$, which indicates the intensity at the bottom of the edge. Here the I_1 value directly determines the intensity at the top side of the edge and thus reflects the SNR of spectra. We then use least square fitting to obtain the fitting coefficients of Eq. (S8), and present the results in **Table S1**, which are averaged from 30 independent simulations. The fitting coefficients error is calculated by Eq. (S9):

$$y = I_2 + \frac{I_1 - I_2}{1 + e^{-a(x-b)}} \tag{S8}$$

$$E = \sqrt{(a - a')^2 + (b - b')^2 + (I_1 - I_1')^2 + (I_2 - I_2')^2} \tag{S9}$$

where a', b', I_1', I_2' are the fitting coefficients of the Eq. (S1).

Table. S1 The coefficients error of the fitting results of the edge profiles with different intensities.

Data	Metrics	Edge parameters: $I_2 = 10, a = 1, b = 0.$				
		I_1=12	I_1=14	I_1=16	I_1=18	I_1=20
Noisy	SNR (dB)	17.36	18.16	19.12	20.12	21.06
	Coefficients error	437.23	42.41	2.62	1.14	0.90
	FWHM error (nm)	N/A	N/A	-0.04±2.0	-0.16±1.3	0.4±1.3
AUnet	SNR (dB)	28.60	29.47	29.88	30.79	31.32
	Coefficients error	1.27	0.98	0.34	0.29	0.31
	FWHM error (nm)	N/A	-0.09±0.7	0.06±0.5	0.02±0.4	0.07±0.3

Therefore, a smaller coefficients error E means a higher accuracy of the fitting of the spatial resolution. Remarkably, the coefficients error of the noisy data exhibits an exponential increase as the SNR decreases. For example, when SNR = 19.12 dB ($I_1 = 16$), the corresponding coefficients error is only 2.62, while when the SNR decreases to 17.36 dB ($I_1 = 12$), the coefficients error dramatically increased to 437.23. In contrast, the results obtained from AUnet demonstrate significantly smaller coefficients error and FWHM errors compared to those obtained from the noisy data for all conditions. Note that the value of FWHM is presented relative to the value of GT (i.e., 3.6 nm as seen in supplementary **Fig. S11**). These findings suggest that AUnet-based denoising can greatly enhance the accuracy of the spatial resolution analysis, yielding results that are much more reliable than the noisy data. Importantly, compared to the noisy image, the positioning accuracy of the AUnet-processed image would allow us to gain a correct structure-spectrum (and thus property) correlation, especially for very tiny atomic structures, for instance in catalysis study.

3. Spectral resolution of noisy and AUnet-denoised spectra

Since it is not straightforward to determine the spectral resolution from the experimental result, we performed simulations to compare the spectral resolution of noisy and AUnet-denoised spectra. For that we created a two-peak Raman spectra dataset (125,000 paired noisy and GT Raman spectra) and trained a new AUnet model. In this dataset, the distance of two peaks ranges from 1 to 100 cm^{-1}, the peak position ranges from 200 to 1400 cm^{-1}, the FWHM of the peaks range from 5 to 200 cm^{-1}, and the intensity of the two peaks range from 5 to 20 counts.

We use approximated Rayleigh criterion to quantify the spectral resolution (**Supplementary Fig. S16**), i.e., for a two-peak spectrum, the relative intensity of the valley and peak should satisfy $I_{valley}/I_{peak} \leq 0.8$ (*D. W. Ball, Field Guide to Spectroscopy, SPIE Press, Bellingham, WA (2006).*). A typical pair of noisy and GT spectra are shown in **Supplementary Fig. S16-b.** The GT spectrum does not fulfill the criterion ($I_{valley}/I_{peak} = 0.85 > 0.8$). For noisy spectra, the valley between two peaks can be either smaller and gives $I_{valley}/I_{peak} = 0.5 < 0.8$, thus fulfilling the Rayleigh criterion (middle panel) or larger than that of GT (right panel). In other words, a noisy spectrum could give a higher or lower spectral resolution than the GT from this single spectrum analysis.

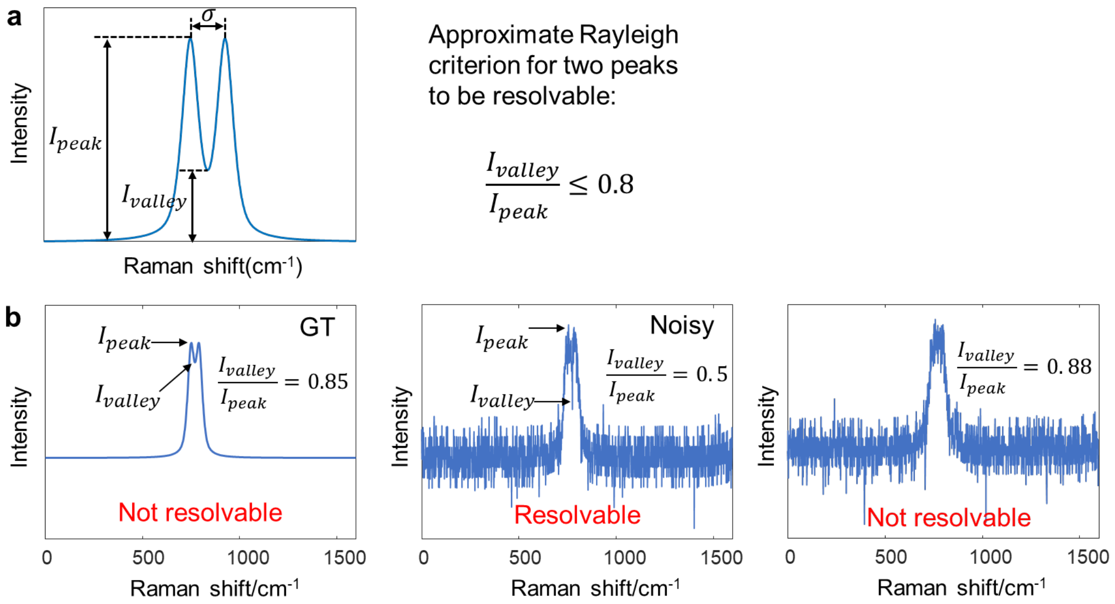

Fig. S16 | Classical Rayleigh criterion for resolving two peaks in a Raman spectrum. (a) The minima sigma (σ) value is the Rayleigh limitation of spectral resolution. The criterion is that the intensity of the valley and peak positions should satisfy $\boldsymbol{I_{valley}/I_{peak} \leq 0.8}$. (b) A comparison shows that the noisy spectrum can give either larger or smaller $\boldsymbol{I_{valley}/I_{peak}}$ value compared to that of the GT spectrum.

To get a statistically meaningful result, we simulated a series of two-peak Raman datasets with varying FWHMs and peak distances, each dataset contains 1,000 spectra. To mitigate the problem for a single spectrum as shown in **Supplementary Fig. S16-b**, we defined the minimum distance of the two peaks when all the 1,000 Raman spectra can be resolved using the approximated Rayleigh criterion. This statistical metric was employed to quantify the required minima peak distance for resolving the two-peak Raman spectra.

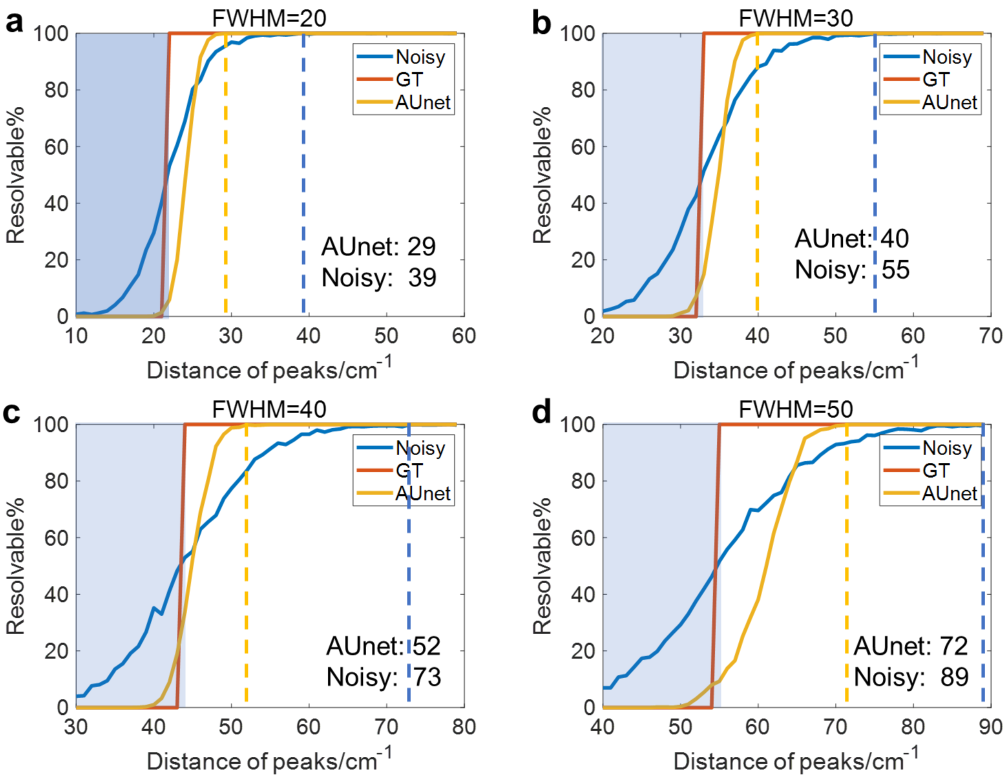

Fig. S17 | Comparison of the probability to resolve two close peaks (i.e., to satisfy the approximated Rayleigh criterion) for GT, noisy, and AUnet spectra. The number of spectra is 1,000. Peak widths are (a) FWHM=20 cm^{-1}, (b) FWHM=30 cm^{-1}, (c) FWHM=40 cm^{-1}, (d) FWHM=50 cm^{-1}. The inset numbers of each panel indicate the peak distance when the probability reaches 100%. For the result in (d), it shows that when the peak distance is 54 cm^{-1}, all the 1,000 spectra satisfy the approximated Rayleigh criterion, i.e., $\boldsymbol{I_{valley}/I_{peak} \leq 0.8}$. For AUnet spectra, this distance increases to 72 cm^{-1}, and for noisy spectra, this distance further increases to 89 cm^{-1}. However, one note that even for a peak distance much smaller than 54 cm^{-1} (blue shaded area), the possibility to resolve two peaks (i.e., satisfying the approximated Rayleigh criterion) is much higher in the noisy spectra.

The results for Raman peaks with different FWHM are depicted in **Supplementary Fig. S17**. Specifically, the data in **Supplementary Fig. S17-d** shows that for the GT spectra without any noise, when the two-peak distance is 54 cm^{-1}, all the 1,000 spectra satisfy the approximated Rayleigh criterion (the brown line), i.e., $I_{valley}/I_{peak} \leq 0.8$. For AUnet spectra (the yellow curve), this distance increases to 72 cm^{-1} as marked by the vertical yellow line, and for noisy spectra, this distance further increases to 89 cm^{-1} (the blue line). The results in other three panels show similar trends. This statistic result suggests that denoised spectra can give better spectral resolution than the noisy counterpart, if we take the peak distance when all (or enough number of) spectra can be resolved as a criterion for spectral resolution. However, one note that even for a peak distance much smaller than 54 cm^{-1} (blue shaded area in **Supplementary Fig. S17-d**), the possibility to resolve two peaks (i.e., satisfying the approximated Rayleigh criterion $I_{valley}/I_{peak} \leq 0.8$) in the noisy spectra (blue curve) is much higher than the GT (brown curve). This comes from the random noisy feature of the spectra as already shown in **Supplementary Fig. S16-b** and the result is, to some extent, unreasonable. The new simulation result shows that it is not straightforward to directly compare the spectral resolution of noisy and AUnet-processed spectra, especially when the number of spectra is limited. Nevertheless, for the results in **Supplementary Fig. S17** with different FWHM, we find that the denoising method reduces the

minima peak distance by a factor of 1.2-1.4 statistically. This fact indicates that the AUnet denoising can improve the accuracy of identifying two neighboring peaks from a statistical point of view.

Supplementary references

[1] He, H. et al. Collaborative Low-Rank Matrix Approximation-Assisted Fast Hyperspectral Raman Imaging and Tip-Enhanced Raman Spectroscopic Imaging. *Anal Chem* **93**, 14609-14617 (2021).

[2] He, H. et al. Automated weak signal extraction of hyperspectral Raman imaging data by adaptive low-rank matrix approximation. *J of Raman Spectrosc* **51**, 2552-2561 (2020).

[3] Konnik, M. & Welsh, J. High-level numerical simulations of noise in CCD and CMOS photosensors: review and tutorial. Preprint at arXiv:1412.4031v1 (2014).

[4] Wei, K., Fu, Y., Yang, J. & Huang, H. A Physics-based Noise Formation Model for Extreme Low-light Raw Denoising. *CVPR*, 2758-2767 (2020).
