# Supplementary material for: Noise learning of instruments for high-contrast, high-resolution and fast hyperspectral microscopy and nanoscopy
Source: Nat Commun. 2024 Jan 25;15:754. doi: 10.1038/s41467-024-44864-5 (PMC10810791; doi:10.1038/s41467-024-44864-5)
Supplement: Supplementary file 3 — Description of Additional Supplementary Files [file 41467_2024_44864_MOESM3_ESM.pdf]

Description of Additional Supplementary Files:

Supplementary Movie 1: Continuous imaging of a HeLa cell labelled by MitoTracker using a laser power of 8.8 µW on the sample.

Supplementary Movie 2: AUnet enhanced continuous imaging of the same HeLa cell in Movie 1.

Supplementary Movie 3: Continuous imaging of a HeLa cell labelled by MitoTracker using a laser power 381 µW on the sample.

Supplementary Movie 4: Photoluminescence imaging of a WS2 sample at a broad wavelength of 600-630 nm.
