# Supplementary material for: Noise learning of instruments for high-contrast, high-resolution and fast hyperspectral microscopy and nanoscopy
Source: Nat Commun. 2024 Jan 25;15:754. doi: 10.1038/s41467-024-44864-5 (PMC10810791; doi:10.1038/s41467-024-44864-5)
Supplement: Supplementary file 2 — Peer Review File [file 41467_2024_44864_MOESM2_ESM.pdf]

nature portfolio

Peer Review File

Noise Learning of Instruments for High-contrast, High-resolution and Fast Hyperspectral Microscopy and Nanoscopy

Open Access This file is licensed under a Creative Commons Attribution 4.0 International License, which permits use, sharing, adaptation, distribution and reproduction in any medium or format, as long as you give appropriate credit to the original author(s) and the source, provide a link to the Creative Commons license, and indicate if changes were made. In the cases where the authors are anonymous, such as is the case for the reports of anonymous peer reviewers, author attribution should be to 'Anonymous Referee' followed by a clear attribution to the source work. The images or other third party material in this file are included in the article's Creative Commons license, unless indicated otherwise in a credit line to the material. If material is not included in the article's Creative Commons license and your intended use is not permitted by statutory regulation or exceeds the permitted use, you will need to obtain permission directly from the copyright holder. To view a copy of this license, visit http://creativecommons.org/licenses/by/4.0/.

Reviewer #1 (Remarks to the Author):

This paper proposes and demonstrates noise reduction in hyperspectral Raman imaging using machine learning of noise characteristics in Raman imaging systems. The main claim of this paper is the versatility of the noise reduction by learning the noise induced in the measurement system. Since this learning scheme is signal independent, the learned network may work for any sample. The idea is interesting and unique, and the demonstration appears attractive for applications requiring high temporal resolution and low irradiation power. However, the noise model used in the learning process would not be able to correctly represent the noise characteristics in Raman imaging.

The authors assumed that the instrumental noise is added to the signal from the sample. However, in Raman imaging, the main reason for the signal fluctuation is the photon shot nose, which is determined by the number of photons of Raman scattering and other backgrounds. Since the high-performance Raman system, which the authors used in the paper, has a shot-noise-limited sensitivity, it is difficult to ignore the contribution of signal to the fluctuation (noise), which would cause situations much different from the authors' assumption and disclaim the versatility.

As the authors mentioned, noise sources also exist in the system, such as scattering or fluorescence from the optics and devices in the system, and it is important to estimate their contributions to measurement results. To do this, the authors placed a gold film and irradiated laser light to measure the instrumental noise. However, gold can also luminesce under irradiation of visible light. In addition, the laser light reflected by the gold film increases the laser intensity in the microscope optics, which induces a background signal more than in the measurement of transparent samples such as cells. From this point, the instrumental noise also reflects the sample condition.

Since it is essential to discuss the signal-to-noise ratio by considering the signal in Raman measurements, as can be seen in another paper (https://doi.org/10.3390/s21155067), it is difficult to be convinced that signal independent noise learning is possible under any circumstances, and this paper is difficult to be accepted in its present form.

Reviewer #2 (Remarks to the Author):

In their manuscript "Noise Learning of Instruments for High-contrast, High-resolution and Fast Hyperspectral Microscopy and Nanoscopy" the authors present a deep learning-based approach to postprocess spectral data in an attempt to reduce/remove unwanted contributions to the recorded data and to improve the signal-to-'noise' ratio which, in turn, would allow for shorter integration times and/or lower laser powers. As opposed to more conventional deep learning approaches, the authors' method characterizes unwanted, instrument-specific contributions to the signal which are subsequently removed from the measured data, and is, thus, agnostic towards the specific sample in question and even to the specific type of spectral information.

The manuscript is generally of high quality and easy to follow. The presented methodology might benefit the broader community. In its current form, the manuscript does, however, require some clarifications with regard to the methodology, as well as some elaboration concerning resolution improvement claims. The manuscript should be considered for publication after major revision.

Preface regarding the notion of 'noise'

The authors claim that their approach allows "accurate removal of the instrumental noise", and improves the "sensitivity, spatial and temporal resolution of hyperspectral Raman microscopy and nanoscopy" (l. 71ff). I was somewhat puzzled as to how such improvements could be achieved based on a noise reduction procedure and found only later that the somewhat unconventional notion of 'noise' was the source of this confusion. Noise in a signal, to my understanding, is generally considered a disturbance with unspecific frequency spectrum, i.e., it refers to random contributions to the overall signal with no correlation between datapoints except potentially for a

very broad distribution in the frequency domain. The authors note that they find “that each Raman instrument exhibits a unique, instrument-specific, statistically stable noise pattern in the frequency domain” (l. 124f). Fig. S1g-h shows that this instrument response consists of very sharp peaks in the frequency domain (on top of a white noise background). The authors should highlight this deviation from a more conventional notion of ‘noise’ more clearly or, perhaps, attempt to avoid this specific wording. Generally, the information content of a recorded signal of any kind is fixed the moment it is recorded. Without considering external information, it cannot be changed retrospectively by any amount of processing like true noise reduction (that is of course not to say that such postprocessing doesn’t help to extract this intrinsic information content). In the context of a microscopic image for example, external information in this sense might refer to knowledge about the point spread functon(either explicitly provided by measuring it / modelling it based on knowledge about the imaging system, or implicitly learned by a machine learning system). Here, loosely speaking, the external information learned by the ‘NL’ algorithm seems to be the instrument response in the frequency domain.

Major points:

(1) Spectral resolution (vs. spatial resolution)
The authors focus heavily on an increased spatial resolution and do not make any claims or comments regarding the spectral resolution, when – to my understanding – it is the spectral resolution that is actively increased with knock-on effects for the spatial resolution in their hyperspectral datasets.
Conceptionally, the method presented by the authors is closer to a background removal than to noise reduction, albeit in the frequency domain (and therefore ‘background’ in this context does not necessarily refer to positionally stable patterns). In the absence of noise, two distinct continuous signals can always be “resolved” with infinite accuracy. In reality, however, the presence of noise and the sampling density limit the ability to resolve different signals and it is reasonable to assume that the unwanted instrument-specific contributions described by the authors will have a similar effect on resolution as true noise if not removed from the dataset. More advanced notions of resolution take noise into account and are likely applicable here as well. The authors should be more specific regarding their notion of resolution and should clearly state how and by how much the removal of the unwanted frequency domain pattern-stable signal contributions increases the spectral resolution. Also, the sampling density should be noted in the manuscript.
While methods that reduce (actual) noise, like smoothing operations, cannot increase true resolution, the additional knowledge about their measurement devices enables the authors to do exactly that in an impressive and apparently practical way. Their method has great potential in my opinion but lacks a solid theoretical frame with clear language. The manuscript would, in my opinion, benefit from a quantification of the spectral resolution enhancement (even though this would clearly be somewhat specific to the authors’ devices).
Some experimental suggestions: (a) Create compound data sets with computational 2-peak spectra and independently measured background + noise (use gold sample as for training data sets). Vary the peak-to-peak separation and quantify the resolution enhancement after processing the compound datasets with the method proposed by the authors. (b) Measure a low-quality Raman spectrum and a very high-quality (i.e. smooth) spectrum of a pure compound. Add true noise to the high-quality spectrum and apply the proposed method. Compare the resulting spectrum with the low-quality and high-quality real spectrum. This will enable the characterization of potential smoothing performed by the proposed method vs. true improvements to the signal.

(2) Spatial resolution
The authors heavily emphasize an increase in spatial resolution in TERS imaging as a result of their data processing. While I agree that, generally, an increase in spectral resolution in both, conventional Raman microscopy as well as TERS, will have positive knock-on effects for the spatial resolution under certain circumstances (i.e. if spectral features are used to differentiate spatially separated features), the interplay of these two factors is not well presented. Such a discussion would be particularly important, as the proposed method does not act on image data for a given wavenumber but processes the spectra pixel-wise. Additionally, the data shown in Fig 3, which provide the only experimental evidence in the manuscript, are insufficient to support the claims of a resolution increase, let alone one by a factor of almost 2. To my understanding, the ‘noisy’ data

in Fig. 3e (left panel), if they were recorded repeatedly, would yield a broad distribution of values for the FWHM shown in Fig. 3g, sometimes suggesting a sharper edge characteristic and sometimes a less sharp edge characteristic (which is used as a proxy for resolution here) than the given 2.9 nm. I suggest using neighboring pixel lines (parallel to the white arrow in Fig. 3dii) to investigate this expectation. Also, in this context, a clearer distinction between positioning accuracy of such an isolated feature and resolution should be made.
Given the known depth of the holes in the palladium layer, is the tip geometry expected to be a limiting factor for spatial resolution when evaluating such a discrete and sharp edge?
It would be interesting to see how the spatial resolution in conventional Raman microscopic images is affected by the authors' processing routine. I suggest measuring a suitable resolution target like a Siemens star (evaluating edge characteristics (as the Cr/Si grating would allow) is insufficient in this regard).

(3) Motivation of deep learning approach
The method the authors suggest appears to be a more complicated version of Fourier filtering where knowledge about the instrument specific response is used to selectively modify the amplitude of certain frequencies in the Fourier transformed spectrum of a given sample. Couldn't such a correction be performed without the need for any deep learning? I suggest the authors try to selectively dampen the relevant frequencies in a measured spectrum according to the instrument specific response (as shown in FigS1g-h) and compare the result to the deep learning-based approach. Which other factors motivated the use of deep learning as opposed to such a conceptionally more simplistic approach?

(4) Clarifications regarding the neural networks
- Networks were trained for differing epoch numbers (200, 100, 2000). What was the reason for that and what was the termination criterion?
- Why was the GT generated differently for the CSL approach than the 'NL' approach? Wouldn't it be better for comparability to use the same method for CSL as for 'NL'? It appears that the CSL approach is limited towards unseen samples because of the selective GT data.
- The 'NL' network was trained in the Fourier domain. It is not entirely clear from the manuscript if the CSL network was trained on the spectra, or their Fourier transform as well. If it wasn't trained in the Fourier domain: why and what would happen if it was done that way?

Minor points:
(1) The authors provide comparisons of their technique and two rather simplistic smoothing routines (Savitzky-Golay, Wavelet) that don't take external information (for example about the peak shape of Raman signals) into consideration. It would be interesting to see comparisons with more sophisticated state-of-the-art post-processing procedures.

(2) Abbreviation 'PIC' not explained upon first mentioning (only in line l. 512 in the last paragraph).

(3) The Supporting Information movies should include a color bar and the intensity scaling should be kept the same between frames. As it is, the constant rescaling makes it difficult to judge bleaching, especially in the first few frames (see 405631_0_video_7196240_rn7x4x.avi) where bleaching should be strongest but individual frames appear virtually unchanged. The same is true for Fig. 4.

(4) Throughout the manuscript the authors claim to have measured live cells (e.g.: l. 82) but the 'methods' section clearly mentions that the cells were fixed (and therefore killed) by 75% ethanol, 10 min. Please clarify.

(5) Figures 1e (GT vs. AUnet) and 2b, 2d (‚local mean' vs. 'NL') seem to suggest that the method proposed by the authors flattens the baseline towards the edges while such behavior is not visible in Figure 2f. Where does this behavior originate from? The CSL approach seems to respect the 'local mean' baseline characteristic.

(6) The figure description for FigS1c is insufficient. It is unclear what the individual panels depict. The authors point towards the method section for more details, but clarification isn't given there

either.

(7) Fig S1a-d and the corresponding part in the method section describe the removal of a global baseline drift from the ‘instrumental noise’ spectra recorded on gold. This step is not motivated in the text. My interpretation is that this is a gold-specific (i.e. not instrument-related), non-Raman contribution which should therefore not be taken into account in further steps. Is this correct? If so, this should be stated in the manuscript.

(8) In the method section, a value of $\rho=0.6785$ is specified for the pseudo-Voigt function. Where does this value originate from? The high numerical precision given hints at some thought being put into this value. It only says, ‘These parameters are randomly extracted in a given range, which should be optimized on a specific Raman instrument’ (l. 421ff). What range and what does ‘randomly extracted’ mean? How sensitive can the method proposed by the authors expected to be with regard to ρ? It would appear that this rather fundamental assumption about the line shape of Raman measurements in the specific device used by the authors might have a significant influence. The peak shape should – to a degree – depend on the device. Why was the same value used for both devices?

(9) In l. 156ff it says, ‘In contrast, the AUnet leads to spectra with significantly improved SNR, which well restores the GT spectra, even if the SNR of the raw spectra was very low or negative’. How could there be a negative SNR? Is this just a numerical problem?

(10) Fig S3b lacks a color bar. Are all data displayed on the same scale? If so, why is there a drop in mean intensities for example in the 518.7 cm-1, 0.02 mW vs. 0.05 mW measurements?

(11) The authors state that there is potential for the ‘application of NL approach for the downstream biological analysis, even possibly for real time analysis’ (l. 354). How long does the processing actually take on a modern computer? Milliseconds? Seconds? Minutes?

(12) Very minor point: Assuming linear axis scaling, figure 1a (left) seems to suggest a linear relationship in Raman microscopy between any two of the parameters speed, resolution, and SNR when the third is set to a target value. Though it is clear that this is supposed to be simplified schematic, it should be noted that this is not true. Accordingly, the proposed benefits of deep learning (right) seem overly optimistic and seem to imply a known and general qualitative relation between the parameters in question upon the application of the method proposed by the authors (‘In this article, we present a noise learning (NL) method that can largely expand the generalizability of deep learning, allowing accurate removal of the instrumental noise, and improve the sensitivity, spatial and temporal resolution of hyperspectral Raman microscopy and nanoscopy (Fig. 1a).’). This strikes me as far too general, and I suggest removing the graphic.

(13) For true noise, different integration times change the composition of said noise (e.g. shot noise vs. read-out noise vs. dark noise) and those contributions generally follow different distributions. Assuming that the frequency-stable unwanted signal contributions are not also phase-stable (in which case the authors’ method would practically amount to a true background subtraction), it is unclear why the model had to be trained on datasets with different integration times. Expectedly for the type of instrument-specific signal under discussion, the normalized amplitudes and frequencies (Fig. S1g-h) do not change significantly with different integration times. Why did the authors deem it necessary to include this additional variable?

Reviewer #3 (Remarks to the Author):

The authors have proposed a deep learning based method to learn noise of the instruments such as Raman microscope(LabRAM) and Raman line scan instrument(Raman-11). The concept is training the 1D U-Net with attention module with instrument noise and high SNR spectra using the spectral generator. The spectrum is processed into DCT coefficient and given as input to 1-D U-NET+attention module and predicted output is obtained by inverse DCT. Now this predicted noise is subtracted from the raw data to obtain the denoised data.

The claims made in the paper is that 10 folds enhancement over SNR, 150 folds improvement over MSE and 2 fold improvement on spatial resolution of 2.9nm in air.
Did author meant here spectral resolution and not the spatial resolution of 2.9nm? In fact, the authors are losing spatial resolution by a significant amount due to 11x11 moving window average. In this case, why not use a low NA imaging setup that inherently covers a larger volume and therefore presents a better averaging anyway.
I worry about the claim of authors regarding drift. I do understand that thermal drift refers to spectral drift in Raman spectrum, but since the authors target microscopy and nanoscopy data, they may want to consider the spatial drift of the sample as well. The spatial drift will result into mixing of two spectra at the boundaries between two materials (for examples Si and Cr in Fig. S4) and therefore simulating just the instrument's other forms of noise may not be sufficient.
At the same time, I wonder about the title containing the words microscopy and nanoscopy. Is the scope of paper nanoscale volumes or are the authors really meaning to use the spatial distribution measured in microscopy and nanoscopy modes. In some way and even if unintended by the authors, it seems that microscopy and nanoscopy terms are added either to bank upon the popularity of the terms or to avoid readership (and review) from spectroscopy community. It does seem that the principles of denoising used in the paper would apply to spectroscopic measurements and microscopy/nanoscopy only present an example situation where SNR may be high. On the same note, when targeting the microscopy and nanoscopy community through the selected title, it is misleading to use high-resolution and claim 2.9 nm spatial resolution enhancement where it seems that the authors mean high spectral-resolution and spectral resolution enhancement of 2.9 nm. Also, is it 2.9 nm resolution or precision? It is likely that I have misunderstood the terminology especially coming from microscopy rather than Raman imaging/spectroscopy background. Nonetheless, the targeted readers are likely to be presented with the same challenges as me. Therefore, the authors need to carefully delineate and if necessary explain the terminology and title in such a way that a general microscopy reader is not confused.
I also find that the novelty of the paper is weak – the use of U-Net and Unet+attention for denoising is plenty both in computer vision and signal processing.
a- Fan, C. M., Liu, T. J., & Liu, K. H. (2022, May). SUNet: swin transformer UNet for image denoising. In 2022 IEEE International Symposium on Circuits and Systems (ISCAS) (pp. 2333-2337). IEEE.
b- Zhang, B., Li, M., Zhou, C., Yang, Q., Wang, L., & Yuan, B. (2022, December). Endoscopic Image Denoising Algorithm Based on Spatial Attention UNet. In Journal of Physics: Conference Series (Vol. 2400, No. 1, p. 012026). IOP Publishing.
c- Qiao, Z., & Du, C. (2022). RAD-UNet: a Residual, Attention-Based, Dense UNet for CT Sparse Reconstruction. Journal of Digital Imaging, 35(6), 1748-1758.
Even Attention+UnEt+DCT has been explored by - Zhang, J., Niu, Y., Shangguan, Z., Gong, W., & Cheng, Y. (2023). A novel denoising method for CT images based on U-net and multi-attention. Computers in Biology and Medicine, 152, 106387. Simulation-supervised learning (which the authors refer to as physics-based learning) is also not unseen.
Based on these observations, it appears to me that the paper is not suitable for publication in Nature Communications.

Blue: Reviewers' comments.
Black: Our responses.
Red: Revisions we made.

Dear Reviewers,

Thank you all for your valuable feedback and comments. We are glad that you recognize the novelty and impact of our work. We appreciate your constructive suggestions, which we have thoroughly addressed with point-to-point responses to your questions attached below.

Thank you for reconsidering our revised manuscript.

Sincerely,
Bin Ren (on behalf of all authors)

Reviewer #1 (Remarks to the Author):

This paper proposes and demonstrates noise reduction in hyperspectral Raman imaging using machine learning of noise characteristics in Raman imaging systems. The main claim of this paper is the versatility of the noise reduction by learning the noise induced in the measurement system. Since this learning scheme is signal independent, the learned network may work for any sample. The idea is interesting and unique, and the demonstration appears attractive for applications requiring high temporal resolution and low irradiation power.

Re: We thank the reviewer for the positive comments of our work and recognizing the significance of our model to be unique and attractive for applications.

However, the noise model used in the learning process would not be able to correctly represent the noise characteristics in Raman imaging. The authors assumed that the instrumental noise is added to the signal from the sample. However, in Raman imaging, the main reason for the signal fluctuation is the photon shot noise, which is determined by the number of photons of Raman scattering and other backgrounds. Since the high-performance Raman system, which the authors used in the paper, has a shot-noise-limited sensitivity, it is difficult to ignore the contribution of signal to the fluctuation (noise), which would cause situations much different from the authors' assumption and disclaim the versatility.

Re: We fully agree with the reviewer that for a high-performance Raman system and when the signal is strong enough (for instance, excited with moderate laser power and for samples with large Raman cross section, long integration time) the noise of the spectra would be photon-shot-noise-limited. The photon shot noise N_S is proportional to the square root of the number of photons, $N_S = \sqrt{n_{\mathrm{photon}}}$. This means when the signal is sufficiently strong N_S would be larger than the readout noise and dark noise of the detector and the noise induced by other fluctuations such as laser power fluctuations and thermal induced movement of the optics/mechanics in path.

<u>We should point out, however, that our proposed model aims to deal with the spectra with significantly reduced signal to noise ratio compared to the photon-shot-noise-limited condition.</u> It should be noted that our model is dedicated to the situation of "*high temporal resolution and low irradiation power*". This includes nano-spectroscopy where the interested signal comes from an area on the order of the square of a few nanometers or even less, and the situation where low excitation power is needed, for instance biological imaging when photobleaching and phototoxicity are problematic. Also crucial for nano-spectroscopy is the requirement of fast imaging to reduce the thermal drift of the sample relative to the tip. These constraints inevitably result in spectra with low signal to noise ratio, for example the TERS spectra in **Fig. 3e** in which the Raman peaks are barely seen on top of the noise. Furthermore, our method is also promising for applications in low-cost, portable (Raman) spectrometers. Such spectrometers usually are not equipped high-standard, cooled CCD detectors, and thus, the noise would be higher than research instruments. Particularly, the signal to noise ratio would become extremely small for trace detection of species with low concentration in practical applications. Therefore, our method is particularly useful for applications with low signal to noise ratio.

In our model, all instrumental noise, including dark noise and readout noise of the detector, are considered because they additively contribute to the measured spectra. The learning algorithm, therefore, learns the noise feature of the spectra that reflects the overall noise contribution of a specific instrument.

As the authors mentioned, noise sources also exist in the system, such as scattering or fluorescence from the optics and devices in the system, and it is important to estimate their contributions to measurement results. To do this, the authors placed a gold film and irradiated laser light to measure the instrumental noise. However, gold can also luminesce under irradiation of visible light. In addition, the laser light reflected by the gold film increases the laser intensity in the microscope optics, which induces a background signal more than in the measurement of transparent samples such as cells. From this point, the instrumental noise also reflects the sample condition.

Re: We thank the reviewer for the professional comment regarding the noise measurement. We apologized for the unclear description that led to the misunderstanding of the noise model we used. Indeed, we pointed out that the gold film also emits and reflects light, and these parts of signal was called **background (BG)** in the original manuscript. Therefore, as demonstrated in the **Supplementary Fig. S1(b-d)** (attached below as **Fig. R1** for the convenience of the reviewer), we propose a noise extraction method to remove the laser and sample induced contribution to the gold film spectra (**Fig. R1-a**). The BG components (**Fig. R1-b**) of the Au film can be easily identified and removed after singular value decomposition (SVD). The remaining components are then summed together to obtain the instrumental noise (**Fig. R1-c**). Note, to perform noise extraction with this method, it is necessary to assemble a 2-D matrix $S_{m\times n}$ from a batch of Au film spectra measured under the same experimental conditions. Subsequently, the proposed method is utilized to process this matrix and extract the noise from the spectral data. To make it clear, we also use **equation (5-6)** in the **Methods** part of the main text (now equation R1-R2 below) to explain this:

$$S_{m\times n} = U_{m\times n}\Sigma_{m\times n}V_{m\times n}^T = [u_1 \quad \cdots \quad u_m] \cdot \begin{bmatrix} \Sigma_k & 0 \\ 0 & 0 \end{bmatrix} \cdot \begin{bmatrix} v_1^T \\ \vdots \\ v_n^T \end{bmatrix}, \ \Sigma_k = \begin{bmatrix} \sigma_1 & \cdots & 0 \\ \vdots & \ddots & \vdots \\ 0 & \cdots & \sigma_k \end{bmatrix} \quad \text{(R1)}$$

where m denotes number of data point in each Raman spectrum determined by the effective readout pixels of the CCD detector, n denotes the total number of the spectra in the matrix. After that, the instrumental noise can be obtained by removing the background components in the above equation. This can be done by manual inspection of each column vector of the left singular matrix $(u_1 \quad \cdots \quad u_m)$, or simply remove the first component $(\sigma_1 \cdot u_1 \cdot v_1^T)$ because it contributes the most majority of the background. By denoting J as the set of background components index, the instrumental noise can be calculated as follows:

$$Noise_{m\times n} = S_{m\times n} - \sum_J \sigma_j \cdot u_j \cdot v_j^T \quad \text{(R2)}$$

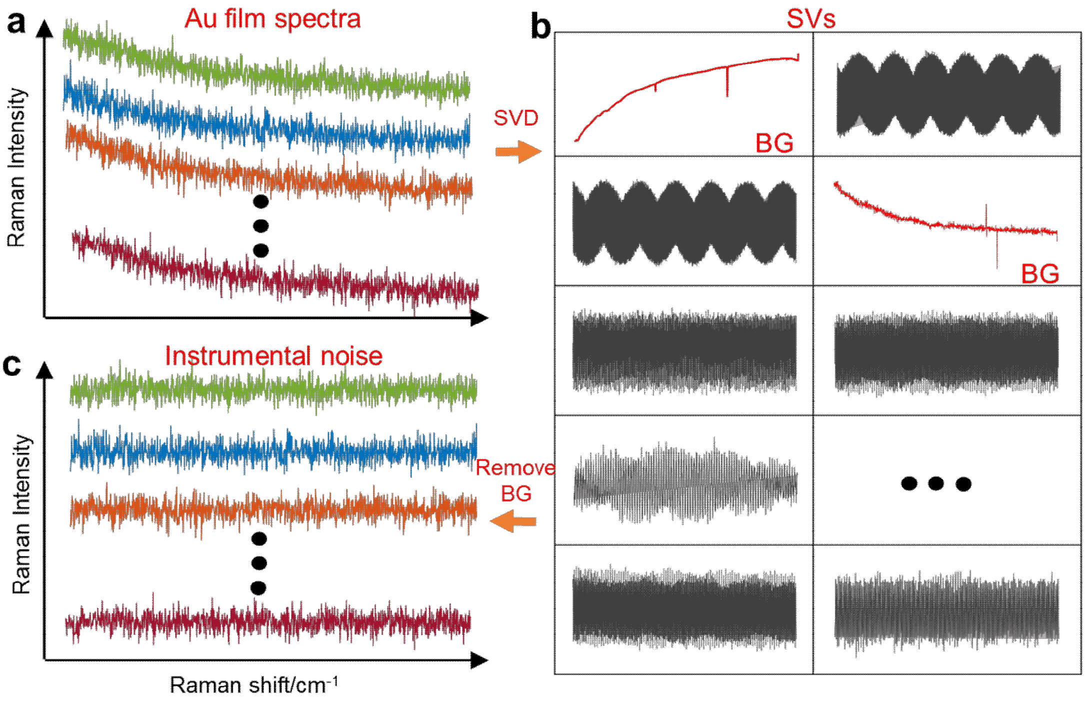

Fig. R1 Instrumental noise extraction method. (a) Raman spectra measured with Au film substrate. (b)The Au film spectra are decomposed using singular value decomposition (SVD). The background (BG) components come from the emission/reflection of Au film are removed, and the remaining SVs components are summed together to obtain the instrumental noise as indicated in (c).

To address the question of the reviewer that different kinds of samples may lead to different noise patterns, we also measured and compared the instrumental noise extracted from a total of 10,000 Raman spectra measured with Au film (highly reflective and luminescent), Si wafer (reflective) and HeLa cell (transparent) samples, and calculate the corresponding mean frequency spectrum. The results (**Fig. R2**) demonstrate that the mean noise patterns of the three samples are quite similar in the frequency domain. The spectra can also be considered as signals distributed in different horizontal pixels of the detector and the Fourier transform (or more specifically the discrete cosine transform we used) of the spectra gives power spectra in the spatial frequency domain with a unit of pixel^{-1}. The results in **Fig. R2** suggests that the instrumental noise has been correctly learned regardless of the type of the sample. The difference of mean instrumental noise spectra of Au film, Si wafer and HeLa cell are caused by the residual Raman features of the specific sample. That is why we suggest to use flat Au film without any Raman features as the reference sample to measure the instrumental noise. Since the noise learning is performed in the spatial frequency domain, it can thus be used to reduce the noise of different kinds of spectra acquired on the same detector, including Raman and fluorescence, because it learns the pixel-wise noise distribution.

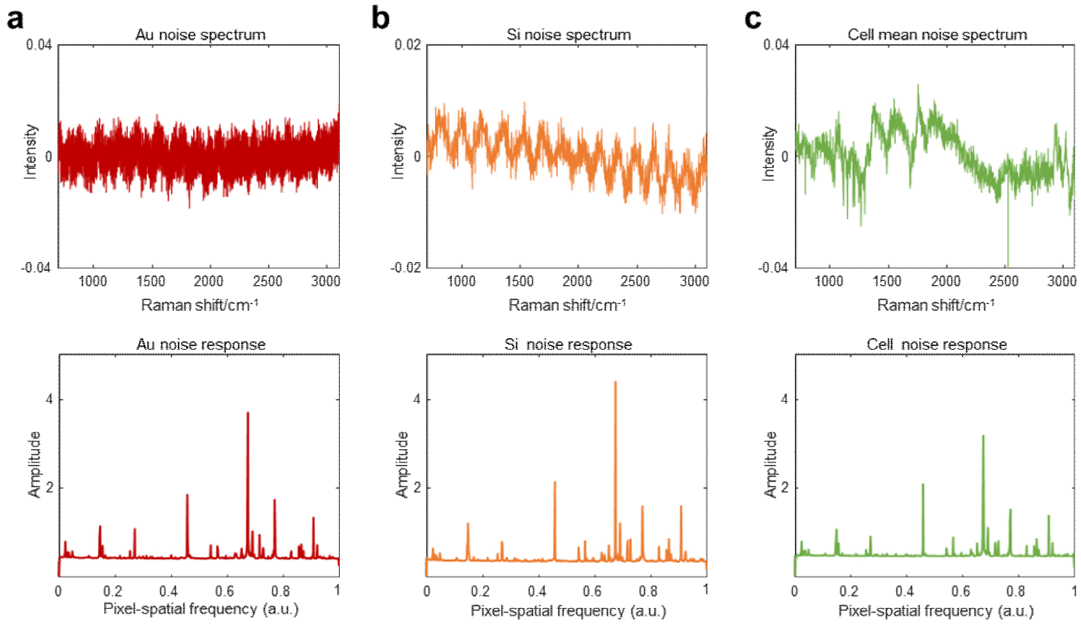

Fig. R2 Instrumental noise analyzing with reflective and transparent samples (Instrumental setup: Raman-11, Nanophoton). Top row: mean noise spectrum obtained with the proposed method in Fig. R1; Bottom row: mean pixel-spatial frequency spectrum after discrete cosine transform of the top row data. (a) Au film sample. (b) Si sample. (c) HeLa cell. These different kinds of samples show similar pattern in the pixel-spatial frequency domain, indicating that the noise has been correctly measured.

Changes: To avoid the misunderstanding about the instrumental noise source, we added the following description in the 'Methods-Instrumental noise measurement' section of the main text.

The instrumental noise in Raman microscopy is indirectly extracted from the spectra obtained from a smooth Au film, which does not exhibit observable Raman peaks (Fig. S1). It is important to note that the Au film can still emit photoluminescence (PL) signals and reflect light, which need to be eliminated from the Au spectra. To this end, we propose the utilization of singular value decomposition (SVD) to separate this sample-based contribution from the instrumental noise.

Since it is essential to discuss the signal-to-noise ratio by considering the signal in Raman measurements, as can be seen in another paper (https://doi.org/10.3390/s21155067), it is difficult to be convinced that signal independent noise learning is possible under any circumstances, and this paper is difficult to be accepted in its present form.

Re: We thank the reviewer for the comments. We have carefully read the paper suggested by the reviewer (Jahn, I.J. et al. Noise Sources and Requirements for Confocal Raman Spectrometers in Biosensor Applications. *Sensors* 21 (**2021**)). It is a very nice work describing the noise source of Raman microscopy. We cite it in our revised manuscript as Ref. 40.

We fully agree that the signal-dependent noise source (multiplicative noise) also degrade the Raman imaging. To our knowledge, most noise sources are additive, such as the relative intensity noise of

the laser [1], dark current noise, and the readout noise of the CCD detector [2-3]. Also, as we mentioned above, we aim at pushing the limits of the Raman microcopy to the challenging experimental conditions, such as low laser power and short integration time. Under such weak SNR conditions, the dominant noise sources come from the instrument setup (optic devices, laser source, detector). Therefore, we think that the additive noise model can approximately represent the real noise in the Raman imaging measurement.

Changes: To make it clear, we have added the following sentence to the “Noise learning with physics-based dataset” part of the main text.

By doing so, the instrumental noise is approximately considered as additive. This is reasonable since the additive noise sources are the major contributors to the instrumental noise especially under the low SNR condition.

References

[1] Irie K, et al. A model for measurement of noise in CCD digital-video cameras. *Meas. Sci. Technol*, 2008, 19(4).

[2] Konnik M, Welsh J. High-level numerical simulations of noise in CCD and CMOS photosensors: review and tutorial. *Preprint at arXiv*:*1412.4031v1*, 2014.

[3] Collin R, et al. Numerical investigation of relative intensity noise in frequency-doubled multimode fiber lasers. *Opt. Commun*, 2021, 485.

Reviewer #2 (Remarks to the Author):

In their manuscript "Noise Learning of Instruments for High-contrast, High-resolution and Fast Hyperspectral Microscopy and Nanoscopy" the authors present a deep learning-based approach to postprocess spectral data in an attempt to reduce/remove unwanted contributions to the recorded data and to improve the signal-to-'noise' ratio which, in turn, would allow for shorter integration times and/or lower laser powers. As opposed to more conventional deep learning approaches, the authors' method characterizes unwanted, instrument-specific contributions to the signal which are subsequently removed from the measured data, and is, thus, agnostic towards the specific sample in question and even to the specific type of spectral information.
The manuscript is generally of high quality and easy to follow. The presented methodology might benefit the broader community. In its current form, the manuscript does, however, require some clarifications with regard to the methodology, as well as some elaboration concerning resolution improvement claims. The manuscript should be considered for publication after major revision.

Re: We appreciate the reviewer for the summary and positive comments to our work and finding our method valuable for the community.

Preface regarding the notion of 'noise'
The authors claim that their approach allows "accurate removal of the instrumental noise", and improves the "sensitivity, spatial and temporal resolution of hyperspectral Raman microscopy and nanoscopy" (l. 71ff). I was somewhat puzzled as to how such improvements could be achieved based on a noise reduction procedure and found only later that the somewhat unconventional notion of 'noise' was the source of this confusion. Noise in a signal, to my understanding, is generally considered a disturbance with unspecific frequency spectrum, i.e., it refers to random contributions to the overall signal with no correlation between datapoints except potentially for a very broad distribution in the frequency domain. The authors note that they find "that each Raman instrument exhibits a unique, instrument-specific, statistically stable noise pattern in the frequency domain" (l. 124f). Fig. S1g-h shows that this instrument response consists of very sharp peaks in the frequency domain (on top of a white noise background). The authors should highlight this deviation from a more conventional notion of 'noise' more clearly or, perhaps, attempt to avoid this specific wording. Generally, the information content of a recorded signal of any kind is fixed the moment it is recorded. Without considering external information, it cannot be changed retrospectively by any amount of processing like true noise reduction (that is of course not to say that such postprocessing doesn't help to extract this intrinsic information content). In the context of a microscopic image for example, external information in this sense might refer to knowledge about the point spread function(either explicitly provided by measuring it / modelling it based on knowledge about the imaging system, or implicitly learned by a machine learning system). Here, loosely speaking, the external information learned by the 'NL' algorithm seems to be the instrument response in the frequency domain.

Re: We thank the referee for pointing out this issue. We shall emphasize, however, that our terminology and meaning of "noise" is the same as the understanding of the referee. That is, it includes readout noise and dark noise of the detector and the noise induced by other fluctuations

such as laser power fluctuations and thermal induced movement of the optics in the optical path. Our results show that, while the noise is assumed to be random, it does lead to some modulation of the intensity in different pixels on the detector. We shall elaborate this point more clearly. While the measured Raman/fluorescence spectra are in the frequency domain, they can also be considered as signals distributed in different horizontal pixels of the detector. Since these pixels are periodically aligned in the horizontal direction, the Fourier transform (or more specifically the discrete cosine transform we used) of the signal (i.e., the spectra) gives power spectra in the pixel-spatial frequency domain with a unit of $pixel^{-1}$. To make it clear, we added a scheme figure to demonstrate this process (**Fig. R3**). Our statistic results show that the noise spectra acquired from each instrument do have a specific pattern in this pixel-spatial frequency domain. This can be clearly seen from the similar pattern in the power spectra from the gold film as well as that from Si wafer and a transparent cell sample (**Fig. R2**). Since the noise learning is performed in the pixel-spatial frequency domain, it can thus be used to reduce the noise of different kinds of spectra acquired on the same detector including Raman and fluorescence because it learns the pixel-wise noise distribution. As also pointed out by the referee, this is the key development of our method and main contribution of our present study. Therefore, our original discussion "*we find that each Raman instrument exhibits a unique, instrument-specific, statistically stable noise pattern in the frequency domain*" is a bit unclear.

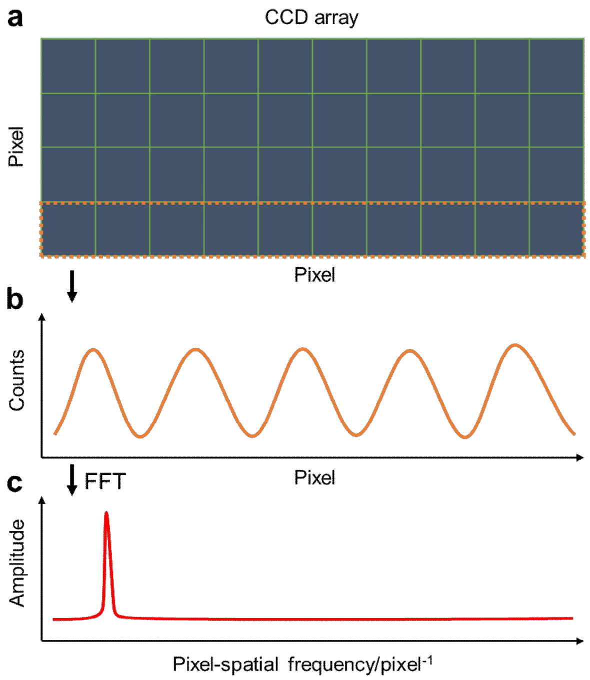

Fig. R3. The practical meaning of Fourier transform (FT) of the spectrum. (a) The scheme of a CCD array that is used as the detector to record the signals in the Raman instruments. (b) The spectrum recorded by the pixel row of the CCD array marked by the dotted rectangle in (a). (c) The fast FT (FFT) transform of the spectrum. Here the horizontal axis corresponds to the pixel-spatial frequency.

Changes: We have now changed the labeling in the figure from "frequency" to "pixel-spatial frequency". In the first paragraph of the Results section, we added the following text.

"statistically stable noise pattern in the pixel-spatial frequency domain (Supplementary Fig. S1g, h, and Fig. S2)"

Fig. R3 has been added as Supplementary Fig. S2 in the revised version.

Major points:

(1) Spectral resolution (vs. spatial resolution)

The authors focus heavily on an increased spatial resolution and do not make any claims or comments regarding the spectral resolution, when – to my understanding – it is the spectral resolution that is actively increased with knock-on effects for the spatial resolution in their hyperspectral datasets.

Re: We thank the referee for the constructive comments. Indeed, we did not make claims about the spectral resolution, because the training dataset we used contains only noisy and GT Raman spectra with the same spectral resolution. Therefore, at the beginning we thought that the well-trained denoising network can only transform the noisy Raman spectra into high SNR ones with identical spectral resolution.

To further illustrate this point, we performed additional simulations to compare the spectral resolution of noisy and AUnet-denoised spectra. We created a two-peak Raman spectra dataset (125,000 paired noisy and GT Raman spectra) and trained a new AUnet model as suggested by the referee. In this dataset, the distance of two peaks ranges from 1 to 100 cm^{-1}, the peak position ranges from 200 to 1400 cm^{-1}, the FWHM of the peaks range from 5 to 200 cm^{-1}, and the intensity of the two peaks range from 5 to 20 counts. Representative testing results are presented in **Fig. R4**.

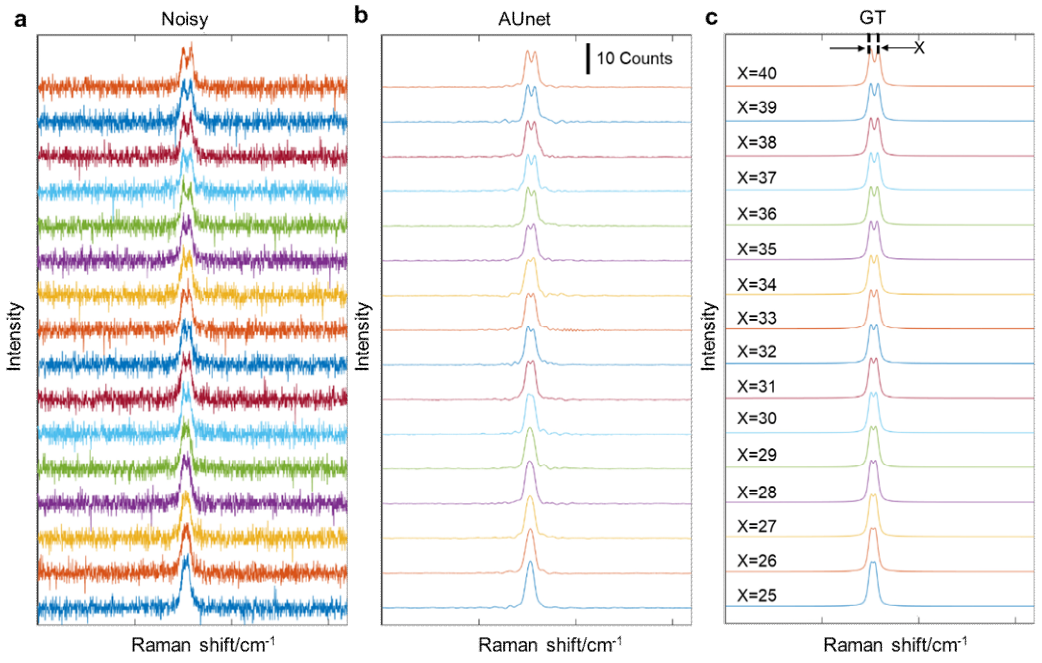

Fig. R4 Testing results with AUnet denoising. (a) Noisy Raman spectra. (b) Denoised by AUnet. (c) GT. X denotes the peak distance between two neighboring peaks.

With these datasets, we can now compare the spectral resolution of the noisy and AUnet-processed spectra. We use approximated Rayleigh criterion to quantify the spectral resolution (**Fig. R5-a**), i.e., for a two-peak spectrum, the relative intensity of the valley and peak should satisfy $I_{valley}/I_{peak} \leq 0.8$ (*D. W. Ball, Field Guide to Spectroscopy, SPIE Press, Bellingham, WA (2006).*). A typical pair of noisy and GT spectra are shown in **Fig. R5-b.** The GT spectrum does not fulfill the criterion ($I_{valley}/I_{peak} = 0.85 > 0.8$). For noisy spectra, the valley between two peaks can be either smaller and gives $I_{valley}/I_{peak} = 0.5 < 0.8$, thus fulfilling the Rayleigh criterion (middle panel) or larger than that of GT. <u>In other words, a noisy spectrum could give a higher or lower spectral resolution than the GT from this single spectrum analysis.</u>

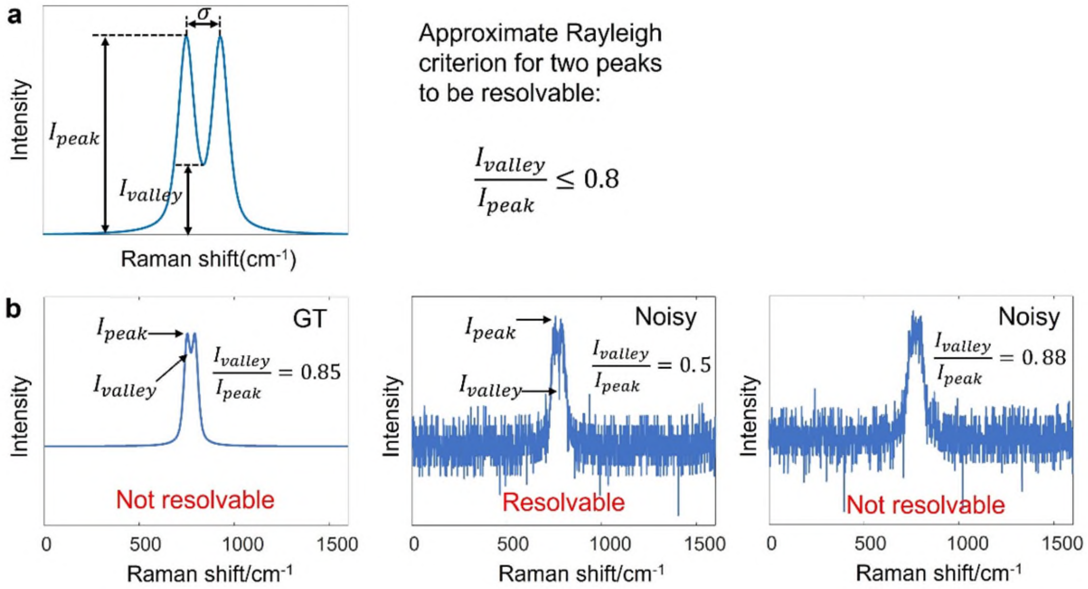

Fig. R5 (a) Classical Rayleigh criterion for resolving two peaks in a Raman spectrum. The minima sigma (σ) value is the Rayleigh limitation of spectral resolution. The criterion is that the intensity of the valley and peak positions should satisfy $I_{valley}/I_{peak} \leq 0.8$. (b) A comparison shows that the noisy spectrum can give either larger or smaller I_{valley}/I_{peak} value compared to that of the GT spectrum.

To get a statistically meaningful result, we simulated a series of two-peak Raman datasets with varying FWHMs and peak distances, each dataset contains 1,000 spectra. To mitigate the problem for a single spectrum as shown in **Fig. R5-b**, we defined the minimum distance of the two peaks when all the 1,000 Raman spectra can be resolved using the approximated Rayleigh criterion. This statistical metric was employed to quantify the required minima peak distance for resolving the two-peak Raman spectra.

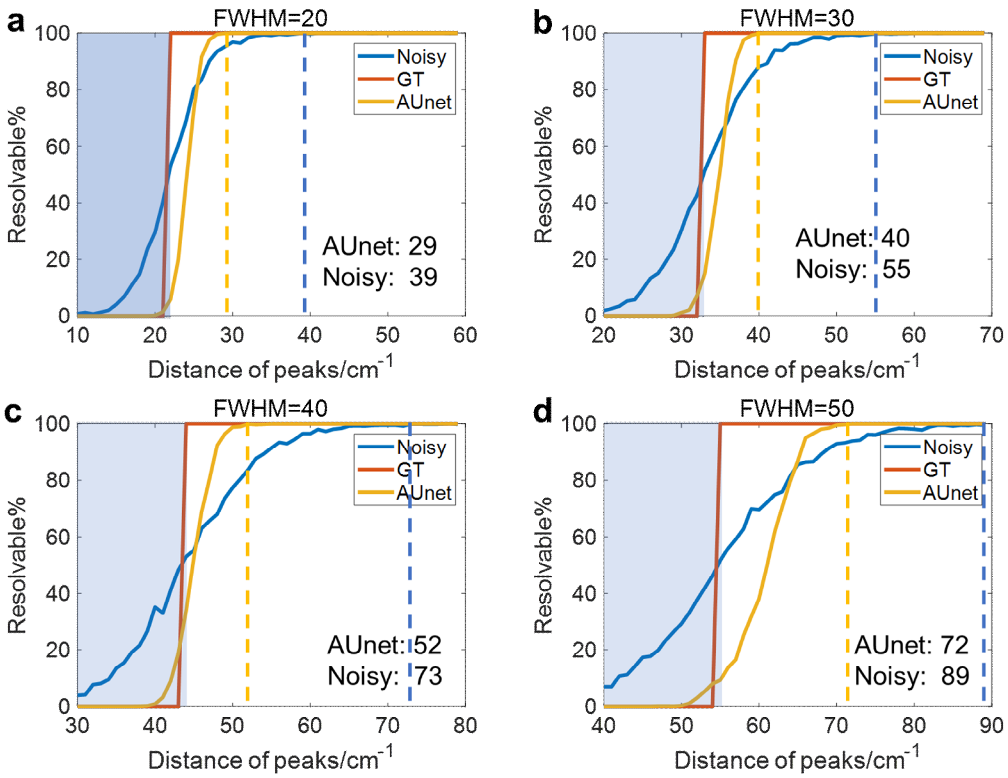

Fig. R6 Comparison of the probability to resolve two close peaks (i.e., to satisfy the approximated Rayleigh criterion) for GT, noisy, and AUnet spectra. The number of spectra is 1,000. Peak widths are (a) FWHM=20 cm^{-1}, (b) FWHM=30 cm^{-1}, (c) FWHM=40 cm^{-1}, (d) FWHM=50 cm^{-1}. The inset numbers of each panel indicate the peak distance when the probability reaches 100%. For the result in (d), it shows that when the peak distance is 54 cm^{-1}, all the 1,000 spectra satisfy the approximated Rayleigh criterion, i.e., $I_{valley}/I_{peak} \leq 0.8$. For AUnet spectra, this distance increases to 72 cm^{-1}, and for noisy spectra, this distance further increases to 89 cm^{-1}. However, one note that even for a peak distance much smaller than 54 cm^{-1} (blue shaded area), the possibility to resolve two peaks (i.e., satisfying the approximated Rayleigh criterion) is much higher in the noisy spectra. This comes from the random noisy feature of the spectra as already shown in **Fig. R5-b** and the result is unreasonable.

The results for Raman peaks with different FWHM are depicted in **Fig. R6**. Specifically, the data in **Fig. R6-d** shows that for the GT spectra without any noise, when the two-peak distance is 54 cm^{-1}, all the 1,000 spectra satisfy the approximated Rayleigh criterion (the brown line), i.e., $I_{valley}/I_{peak} \leq 0.8$. For AUnet spectra (the yellow curve), this distance increases to 72 cm^{-1} as marked by the vertical yellow line, and for noisy spectra, this distance further increases to 89 cm^{-1} (the blue line). The results in other three panels show similar trends. **This statistic result suggests that denoised spectra can give better spectral resolution than the noisy counterpart, if we take the peak distance when all (or enough number of) spectra can be resolved as a criterion for spectral resolution.** However, one note that even for a peak distance much smaller than 54 cm^{-1} (blue shaded area in **Fig. R6-d**), the possibility to resolve two peaks (i.e., satisfying the approximated Rayleigh criterion $I_{valley}/I_{peak} \leq 0.8$) in the noisy spectra (blue curve) is much higher than the GT (brown curve). This comes from the random noisy feature of the spectra as already shown in **Fig. R5-b** and the result is, to some extent, unreasonable.

The new simulation result shows that it is not straightforward to directly compare the spectral resolution of noisy and AUnet-processed spectra, especially when the number of spectra is limited. Nevertheless, for the results in **Fig. R6** with different FWHM, we find that the denoising method reduces the minima peak distance by a factor of 1.2-1.4 statistically. **This fact indicates that the AUnet denoising can improve the accuracy of identifying two neighboring peaks from a statistical point of view.**

Conceptionally, the method presented by the authors is closer to a background removal than to noise reduction, albeit in the frequency domain (and therefore 'background' in this context does not necessarily refer to positionally stable patterns). In the absence of noise, two distinct continuous signals can always be "resolved" with infinite accuracy. In reality, however, the presence of noise and the sampling density limit the ability to resolve different signals and it is reasonable to assume that the unwanted instrument-specific contributions described by the authors will have a similar effect on resolution as true noise if not removed from the dataset. More advanced notions of resolution take noise into account and are likely applicable here as well. The authors should be more specific regarding their notion of resolution and should clearly state how and by how much the removal of the unwanted frequency domain pattern-stable signal contributions increases the spectral resolution. Also, the sampling density should be noted in the manuscript.

Re: We thank the referee for the comments. Our method is a true noise reduction approach by noise learning in the pixel-spatial frequency domain. As discussed above, the ability to resolve two peaks should satisfy certain condition and can be quantified by the Rayleigh criterion. The spectral resolution also depends on the instrument hardware parameters (groove density of grating, slit width, pixel size, etc.). In our experiment of Raman measurement, the sampling density is 1.2 cm^{-1}/pixel for LabRAM HR-Evolution (Horiba) and 4 cm^{-1}/pixel for Raman-11 (Nanophoton) instruments.

Changes: We have added the sampling density to the Methods - Optical measurements section.

While methods that reduce (actual) noise, like smoothing operations, cannot increase true resolution, the additional knowledge about their measurement devices enables the authors to do exactly that in an impressive and apparently practical way. Their method has great potential in my opinion but lacks a solid theoretical frame with clear language. The manuscript would, in my opinion, benefit from a quantification of the spectral resolution enhancement (even though this would clearly be somewhat specific to the authors' devices).

Re: We appreciate the reviewer for the professional and constructive comments regarding on the spectral resolution. In the past three months, we have been intensively working on comparing the spectral resolution before and after denoising of Raman spectra. Our new simulation shown above indicates that a quantification of the spectral resolution enhancement is not straightforward (**Fig. R5-R6**). A more detailed study should be carried out to address this topic. However, we feel that an open discussion based on our new simulation can be provided, particularly when analyzing the spatial resolution discussed below, following the referee's suggestion.

Changes: We have added the results in **Fig. R5-R6** and related discussion to supplementary information.

Some experimental suggestions: (a) Create compound data sets with computational 2-peak spectra and independently measured background + noise (use gold sample as for training data sets). Vary the peak-to-peak separation and quantify the resolution enhancement after processing the compound datasets with the method proposed by the authors. (b) Measure a low-quality Raman spectrum and a very high-quality (i.e. smooth) spectrum of a pure compound. Add true noise to the high-quality spectrum and apply the proposed method. Compare the resulting spectrum with the low-quality and high-quality real spectrum. This will enable the characterization of potential smoothing performed by the proposed method vs. true improvements to the signal.

Re: We thank the referee for the suggestions. We have followed these suggestions and performed new simulations (**Fig. R5-R6**), which show that our denoising network can improve the SNR and give a spectral resolution that is much closer to the GT data compared to the noisy spectra. We think that a more reasonable way to improve the spectral resolution is to train a super-resolution network to transform the low-resolution Raman spectra into high-resolution ones, with a dataset containing paired low- and high- resolution data.

(2) Spatial resolution

The authors heavily emphasize an increase in spatial resolution in TERS imaging as a result of their data processing. While I agree that, generally, an increase in spectral resolution in both, conventional Raman microscopy as well as TERS, will have positive knock-on effects for the spatial resolution under certain circumstances (i.e. if spectral features are used to differentiate spatially separated features), the interplay of these two factors is not well presented. Such a discussion would be particularly important, as the proposed method does not act on image data for a given wavenumber but processes the spectra pixel-wise.

Re: We fully agree that an increase in spectral resolution may help to improve the spatial resolution of a Raman imaging. Our simulation and reply above show that a quantification of the spectral resolution enhancement is difficult. The statistic result, however, do suggest that denoised spectra can potentially give better spectral resolution than the noisy counterpart when a large number of spectra are analyzed. Therefore, this could be helpful in improving the spatial resolution as noted by the referee. We add some discussion on this point.

Changes: In the paragraph discussing about the spatial resolution enhancement of the TERS images, we added the following discussion:

The spatial resolution enhancement may benefit from an increase of spectral resolution since our denoising method reduces the noise for the spectra in each pixel of the TERS image. We have analyzed the spectral resolution before and after denoising by simulation, and the statistic result suggests that denoised spectra can give better spectral resolution than the noisy counterpart, if a large number of spectra are analyzed (Supplementary Fig. S-15). This may potentially increase the spatial resolution. A more detailed study is, however, needed to address the interplay between spectral and spatial resolution enhancement.

Additionally, the data shown in Fig 3, which provide the only experimental evidence in the manuscript, are insufficient to support the claims of a resolution increase, let alone one by a factor of almost 2. To my understanding, the 'noisy' data in Fig. 3e (left panel), if they were recorded repeatedly, would yield a broad distribution of values for the FWHM shown in Fig. 3g, sometimes suggesting a sharper edge characteristic and sometimes a less sharp edge characteristic (which is used as a proxy for resolution here) than the given 2.9 nm. I suggest using neighboring pixel lines (parallel to the white arrow in Fig. 3dii) to investigate this expectation. Also, in this context, a clearer distinction between positioning accuracy of such an isolated feature and resolution should be made.

Re: We really appreciate the reviewer for the constructive suggestion. Following this, we analyzed more carefully our experimental results and carried out additional simulation to investigate the issue of spatial resolution.

Experimentally, it is a common practice to take a line intensity profile along a certain structure feature. This feature (like the edge used in our manuscript) would give a clear spectral contrast that allows us to estimate the spatial resolution [7] (*Nature*, 2013, 498, 82–86.). Since spatial resolution necessarily reflects the extreme resolving ability of the setup, it is reasonable to take the smallest values one can get from a measurement, provided that the SNR is enough. We do, however, agree with the reviewer that taking a single line may not be enough to compare the spatial resolution of the noisy and denoised images. Therefore, we would like to use a statistic metric to demonstrate the benefits of AUnet denoising in terms of the spatial resolution.

As the reviewer suggested, we picked up 6 neighboring lines parallel to the white arrow in **Fig. 3dii** of the main text, and calculate the mean FWHM and its standard deviation. The zoom-in TERS images of the edge region around the white arrow are shown **Fig. R7a,** in which the left region is Au and the right region is Pd covered Au. The AUnet-processed image shows much clearer distinction between the left and right regions, whereas the intensity distribution in the noisy (raw) image is much more spread, making it very difficult to identify the edge structure. The fitted FWHM are shown in **Fig. R7b** for the 6 horizontal lines in **Fig. R7a** that is parallel to the white arrow in **Fig. 3dii** of the original main text. The spatial resolution has been improved from 7.4 to 6 nm (1.23 times) and the standard deviation (SD) reduces from 3.7 to 2.5 nm.

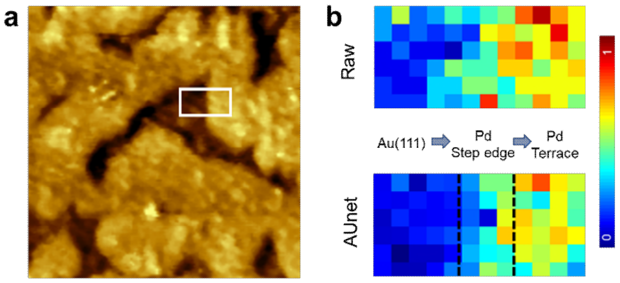

c

	Raw		AUnet	
Row number	R-squared	FWHM (nm)	R-squared	FWHM (nm)
1	0.90	6.8	0.93	6.9
2	0.99	2.1	0.96	3.8
3	0.96	3.9	0.94	8.5
4	0.95	10.1	0.99	2.5
5	0.96	10.3	0.94	8.8
6	0.95	11.1	0.97	5.4
Mean ± SD		7.4±3.7		6±2.5

Fig R7. Spatial resolution analysis of the noisy and AUnet-processed TERS image of the original region shown in Fig. 3 in the manuscript. (a) STM image of the sample. (b) Zoom-in TERS images of the white rectangular region in (a). The AUnet-processed image shows much clearer distinction between the left (Au) and right (Pd covered Au) regions, whereas the intensity distribution in the noisy (raw) image is much more spread. (c) Fitted results using the derivative of Eq. (4) for the 6 horizontal lines in the image shown in (b). The results indicate that the AUnet denoising can reduce the standard deviation (2.5 nm vs 3.7 nm) of the estimated FWHMs, and the spatial resolution has been slighted improved from 7.4 to 6 nm (1.23 times).

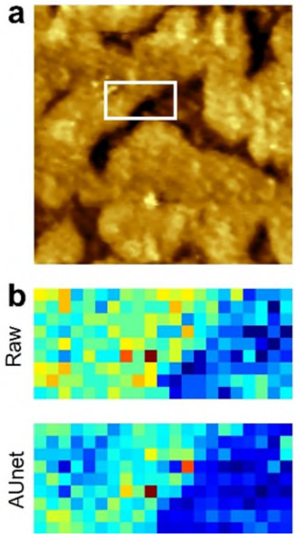

c

	Raw		AUnet	
Row number	R-squared	FWHM (nm)	R-squared	FWHM (nm)
1	0.61	3.7	0.88	3.3
2	0.65	5.4	0.71	4.6
3	0.58	N/A	0.72	4.3
4	0.67	7.6	0.88	4.8
5	0.92	9.3	0.89	2.7
6	0.02	N/A	0.84	3.4
7	0.87	4.8	0.94	4.0
8	0.68	2.4	0.87	4.3
9	0.05	N/A	0.90	2.9
Mean± SD		5.5 ± 2.5		3.8 ± 0.76

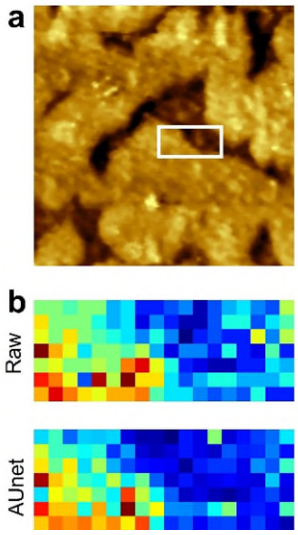

c

	Raw		AUnet	
Row number	R-squared	FWHM (nm)	R-squared	FWHM (nm)
1	0.96	7.4	0.98	4.6
2	0.82	2.2	0.81	N/A
3	0.66	12.7	0.93	3.5
4	0.96	N/A	0.93	N/A
5	0.02	N/A	0.89	5.5
6	0	N/A	0.96	4.8
7	0.70	14.7	N/A	N/A
Mean± SD		9.3±5.6		4.6±0.8

Fig. R8. Another two step edge regions for the analysis of the spatial resolution.

To get more reliable analysis of the spatial resolution enhancement, we further examined another two regions shown in **Fig. R8**. In these regions, the spatial resolution has been enhanced by 1.45 times (from 5.5 to 3.8 nm, top) and 2 times (from 9.3 to 4.6 nm, bottom). More importantly, the standard deviation of the fitting curve reduces significantly (from 2.5 to 0.76 nm in the top image and from 5.6 to 0.8 nm in the bottom image) for the AUnet processed images. This suggests that the spatial resolution from different parallel lines is much closer in the AUnet processed image and thus the result is much more reliable. This agrees with the observation that AUnet processed image matches much better to the STM topography compared to the noisy image, showing a better positioning accuracy of the AUnet images (as supported by new simulation below) that offers a reliable analysis of the local structure-spectra (properties) correlation.

To numerically compare the spatial resolution of a noisy and AUnet processed image and investigate the spatial resolution enhancement, we simulated a series of Raman imaging dataset with a horizontal edge structure (marked by the dashed rectangle in **Fig. R9-a**). The image size is 30×30 pixels. The edge step is generated using the Sigmoid function as follows:

$$y = a + \frac{b}{e^{-x}} \qquad \text{(R3)}$$

where *a* and *b* indicate the bottom and the top intensity of the edge, respectively. Clearly, the AUnet-processed image is much closer to the GT one, whereas the noisy image is very burry in which the edge structure is almost lost. The AUnet and noisy images reproduce well the features of the experimental results shown in **Fig. 3** in the manuscript. **Fig. R9-b** shows spectra along the vertical lines in **Fig. R9-a** crossing the edge. Intensity change at the edge is clear in the GT data but almost disappear in the noisy image. Importantly, each pixel in the image contains a Raman spectrum with identical peak position and FWHM (**Fig. R9-c**), and only the intensity is changed across the edge structure from top to bottom.

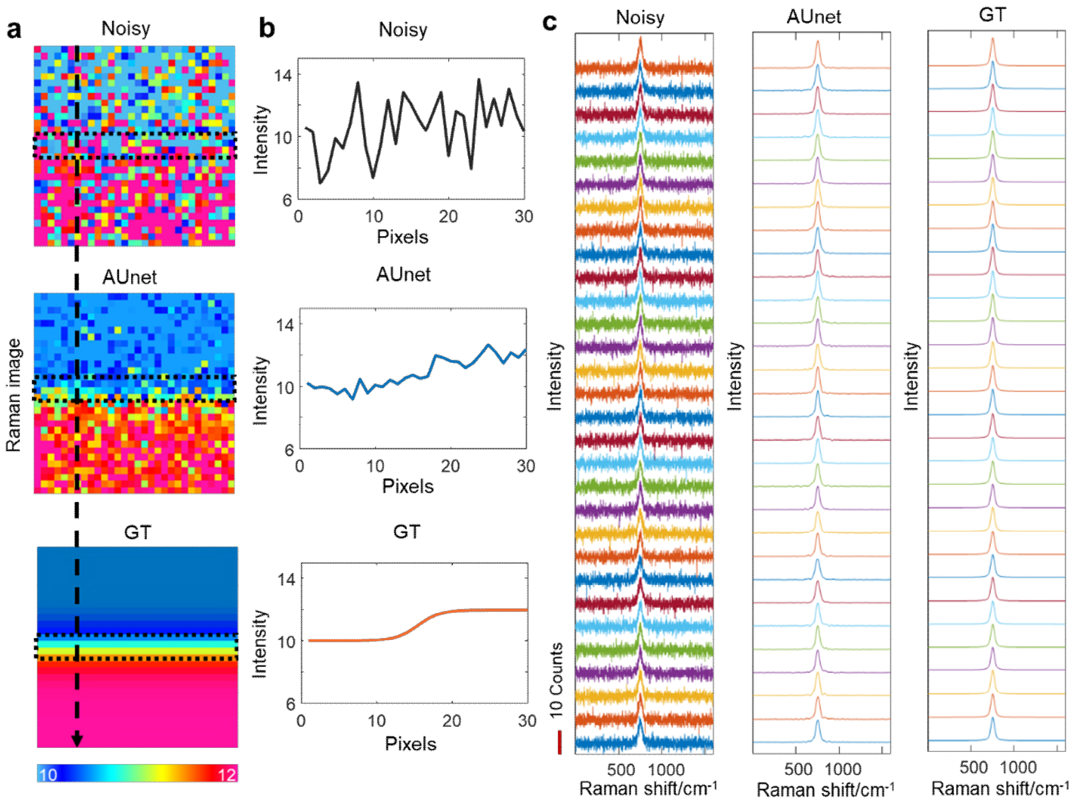

Fig. R9 Simulated Raman imaging data with a horizontal edge structure. (a) Simulated Raman image (using the peak intensity at 750 cm^{-1}) with a horizontal edge structure marked by the dashed rectangles. From top to bottom panel are Noisy, AUnet-processed and GT Raman image with 30×30 pixels. In this typical example, the intensity at the bottom of the edge is 10, and at the top is 12. (b) The Raman peak intensity line profile along the black dashed vertical line marked on the Raman images in (a). From top to bottom panel are Noisy, AUnet-processed and GT line profiles with 30 pixels. (c) The Raman spectra along the black dashed line marked on the Raman images in (a). From left to right are Noisy, AUnet-processed and GT Raman spectra.

Next, we use the following method (the same that we used in our original manuscript as well as in other references [4-6]) to calculate the spatial resolution using the intensity of the edge profile:

1. Fit the line profile (**Fig. R9-b**) using the following function:

$$y = I_2 + \frac{I_1 - I_2}{1+e^{-a(x-b)}} \tag{R4}$$

2. Calculate the first derivative of the fitting result of y, denoting it as y'.
3. Calculate the FWHM of y', and the FWHM is taken as the spatial resolution of the measurement.

As a typical example, **Fig. R10** presents the results by using the above method to calculate the spatial resolution of the edge-like line profiles that are generated by Eq. (R4) with a = 10 and b = 20. **Fig. R10-a** shows the fitted line profiles (red curves), and the corresponding first derivative curves are presented in **Fig. R10-b**. In this typical case, the calculated FWHM of the noisy data is smaller even than that of the GT data, meaning the spatial resolution determined from noisy image can be higher than the GT data. This is consistent with the reviewer's comment. Therefore, we think

that care should be taken when using this FWHM to indicate the spatial resolution of TERS images in the cases of noisy image constructed from spectra with very weak SNR, because such artefacts cannot be avoided due to the existed noise of the data.

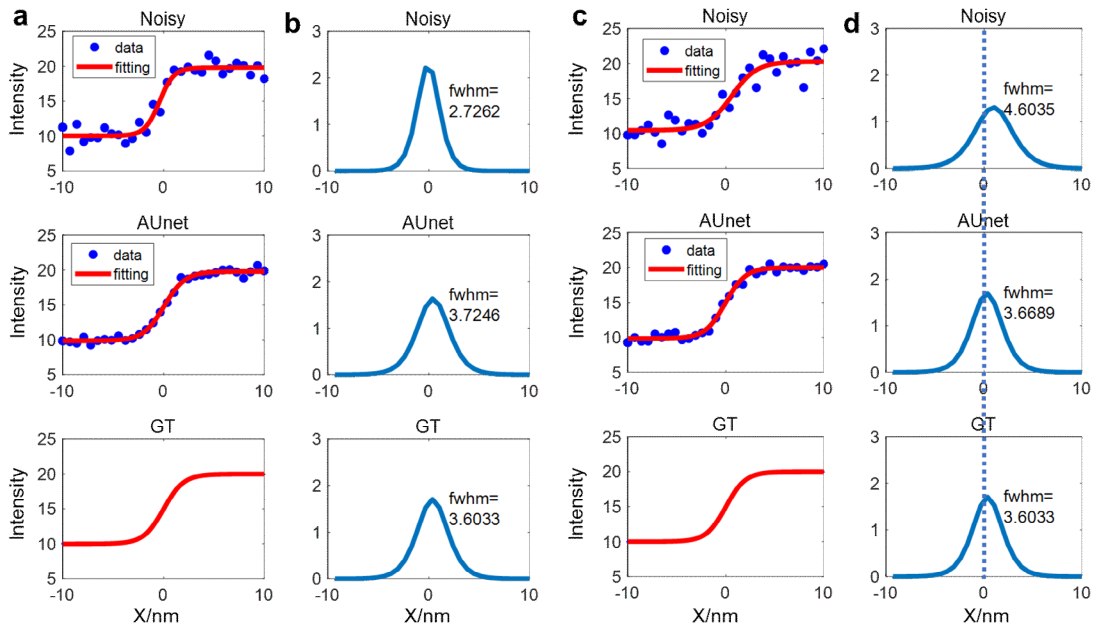

Fig. R10 Calculation of spatial resolution of the simulated line profiles. (a) Line profiles of peak intensity of the simulated Raman spectra, and fitting results using Eq. (4). From the top to bottom panel are Noisy, AUnet-processed and GT results, respectively. In this typical example, the bottom intensity of the edge is 10, and the top is 20. (b) The first derivative curves corresponding to the red curves of (a), and the calculated FWHMs. (c-d) Another case similar to (a-b).

Although the gained SNR from the denoising process do not significantly enhance the spatial resolution, it can improve the positioning accuracy. This can be observed in another case as shown in **Fig. R10-c, d**. As indicated by the vertical dotted line (**Fig. R10-d**), the peak position of the noisy spectrum deviates from the GT data, whereas the AUnet-processed data aligns perfectly with the GT. More importantly, from the two cases shown in **Fig. R10**, we see that the FWHM of the AUnet-processed data is much closer to that of the GT data, whereas the noisy spectra can either show increased or decreased FWHM (i.e., spatial resolution) compared to that of GT.

To statistically quantify this observation, we generated a series of edge structure with different parameters (I_1, I_2, a, b). Here for simplicity, we set $a = 1$, $b = 0$, and $I_2 = 10$, which indicates the intensity at the bottom of the edge. Here the I_1 value directly determines the intensity at the top side of the edge and thus reflects the SNR of spectra. We then use least square fitting to obtain the fitting coefficients of Eq. (R4), and present the results in **Table R1**, which are averaged from 30 independent simulations. The fitting coefficients error is calculated by Eq. (R5):

$$E = \sqrt{(a - a')^2 + (b - b')^2 + (I_1 - I_1')^2 + (I_2 - I_2')^2} \quad \text{(R5)}$$

where a', b', I_1', I_2' are the fitting coefficients of the Eq. (R4).

Therefore, a smaller coefficients error E means a higher accuracy of the fitting of the spatial

resolution. Remarkably, the coefficients error of the noisy data exhibits an exponential increase as the SNR decreases. For example, when SNR = 19.12 dB ($I_1 = 16$), the corresponding coefficients error is only 2.62, while when the SNR decreases to 17.36 dB ($I_1 = 12$), the coefficients error dramatically increases to 437.23. In contrast, the results obtained from AUnet demonstrate significantly smaller coefficients and FWHM errors compared to those obtained from the noisy data for all conditions. Note that the value of FWHM is presented relative to the value of GT (i.e., 3.6 nm as seen in **Fig. R10**). **These findings suggest that AUnet-based denoising can greatly enhance the accuracy of the spatial resolution analysis, yielding results that are much closer to the GT data and more reliable than the noisy data.** Importantly, compared to the noisy image, the positioning accuracy of the AUnet-processed image would allow us to gain a correct structure-spectrum (and thus property) correlation, especially for very tiny atomic structures, for instance in catalysis study.

Table R1. The coefficients error of the fitting results of the edge profiles with different intensities.

Data	Metrics	Edge parameters: $I_2 = 10, a = 1, b = 0.$				
		I_1=12	I_1=14	I_1=16	I_1=18	I_1=20
Noisy	SNR (dB)	17.36	18.16	19.12	20.12	21.06
	Coefficients error	437.23	42.41	2.62	1.14	0.90
	FWHM error (nm)	N/A	N/A	-0.04±2.0	-0.16±1.3	0.4±1.3
AUnet	SNR (dB)	28.60	29.47	29.88	30.79	31.32
	Coefficients error	1.27	0.98	0.34	0.29	0.31
	FWHM error (nm)	N/A	-0.09±0.7	0.06±0.5	0.02±0.4	0.07±0.3

To summarize for the spatial resolution issue, we find that the spatial resolution has been increased by 1.2-2 times when analyzing different surface regions and taking the averaged value of multiple parallel lines, as suggested by the referee. Our new simulation shows that the spatial resolution enhancement is not very obvious. The simulation, however, demonstrates that the positioning accuracy of the AUnet-processed image is much better than that in the raw noisy image, which is also confirmed in our experimental data. This will help to establish a clear correlation between structure and spectra. We have changed the manuscript accordingly.

Changes: We have used the averaged value of multiple parallel lines for the analysis of the spatial resolution. The revised Fig. 3 in the main text is shown in **Fig. R11** below. The two new regions shown in Fig. R8 have been added to the supplementary information as Fig. S13. We revised the discussion of the 2-fold spatial resolution improvement of TERS imaging after AUnet denoising throughout our revised manuscript by incorporating the simulations and discussions above.

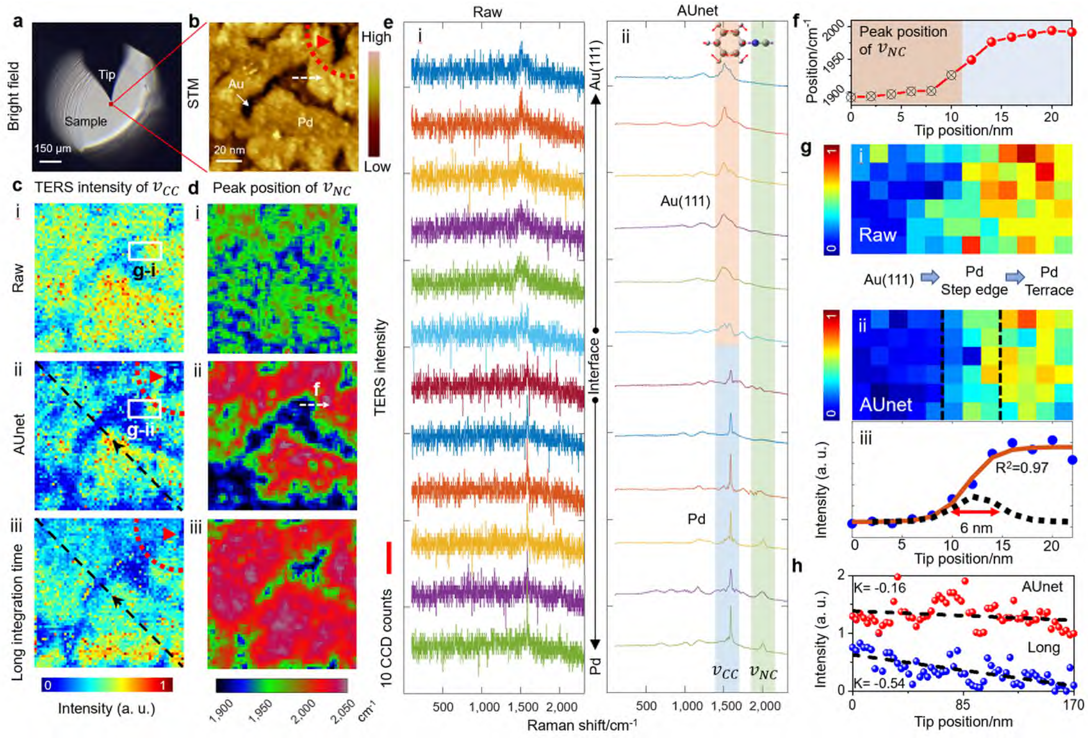

Fig. R11. Revised version of Fig. 3.

The revised text is as follows.

The spatial resolution and positioning accuracy of a TERS image is crucial for obtaining a clear local structure-property correlation. The zoom-in TERS intensity images of the ν_{CC} *band (marked by the white rectangles in Fig. 3c) are shown in Fig. 3g. In this zoom-in region, the left part is the Au(111) surface and right part is Au(111) covered by monoatomic layer Pd with an edge structure in-between as seen in the STM image (Fig. 3b). The TERS intensity in the raw data (Fig. 3g-i) spreads rather randomly, which does not match well with the STM image. In contrast, the transition from Au(111) to Pd regions (left to right) can be clearly resolved in the AUnet-denoised image (Fig. 3g-ii), implying that the positioning accuracy has been improved by denoising. We have simulated noisy and AUnet-processed Raman images containing an edge structure (supplementary Fig. S10), which reproduces the experimental result well. Further analysis reveals that AUnet denoising substantially improves the positioning accuracy of the TERS imaging result (supplementary Fig. S11 and supplementary discussion). The spatial resolution has been improved from 7.4 to 6 nm (Fig. 3g-iii) for the data presented in Fig. 3g (see "Evaluation of spatial resolution of TERS" of the Methods part and supplementary Fig. S12 for details). Analysis of more regions indicates that the spatial resolution is improved by 1.2-2 times after denoising (Supplementary Fig. 13).*

Given the known depth of the holes in the palladium layer, is the tip geometry expected to be a limiting factor for spatial resolution when evaluating such a discrete and sharp edge?

Re: Yes, it is true that the radius of apex of the metal tip is a dominant factor that determines the spatial resolution of TERS. More precisely, it is the spatial profile of the localized electromagnetic (EM) field T(*x*) between the tip and the substrate (which depends on radius of tip apex) that

determines the spatial resolution. The measured TERS signal S(x) can be considered as the convolution of T(x) and the sample feature R(x), i.e., $S(x) = T(x) \otimes R(x)$ [5]. Since such sharp edge is expected to have a R(x) on single atomic level, and the apex radius usually is on the order of 10 nm, we have $S(x) \approx T(x)$. This is why such an edge structure is often used to estimate the spatial resolution in TERS community.

However, one should note that sometimes very tiny structures at the apex of the tip can further localize the EM field to a scale much smaller than the radius. That is why spatial resolution below one nanometer has been achieved in TERS under ultrahigh vacuum and ultralow temperature.

It would be interesting to see how the spatial resolution in conventional Raman microscopic images is affected by the authors' processing routine. I suggest measuring a suitable resolution target like a Siemens star (evaluating edge characteristics (as the Cr/Si grating would allow) is insufficient in this regard).

Re: We thank the reviewer for the comment. As we discussed above, our analysis shows that the denoising method does not significantly improve the spatial resolution, but can increase the positioning accuracy.

Nonetheless, we manufactured a triangle structure (**Fig. R12**) on an Si/Cr-Au sample similar to the Siemens star which can be used to calibrate the spatial resolution of Raman images. In this structure, the triangle region is Si and the outer area is coated with 50 nm Cr and 50 nm Au. The Raman intensity of the Si peak is then used for imaging and analysis of spatial resolution. Notably, at the top of this triangle region, the width is below 30 nm (given by the resolution of electron beam lithography, EBL), much smaller than the expected spatial resolution of $r = 0.6\lambda/NA = 0.6 \times 532\,\text{nm}/0.9 = 355\,\text{nm}$ for the used microscope. Therefore, the cross section of the top region can be used to estimate the spatial resolution. At the tiny tip cross section (marked by the orange dotted line in **Fig. R13-a**), the AUnet data yields a spatial resolution of 400 nm which is very close to the calculated theory spatial resolution of the instrument system, while the Raw data cannot differentiate the tiny structure in this case (**Fig. R13-b**). In another case marked by the white dotted line (below the orange one), the spatial resolution of the AUnet image is 450 nm, whereas the value is 410 nm for the noisy image (**Fig. R13-c**). The smaller/better spatial resolution of the noisy image is consistent with the simulation above.

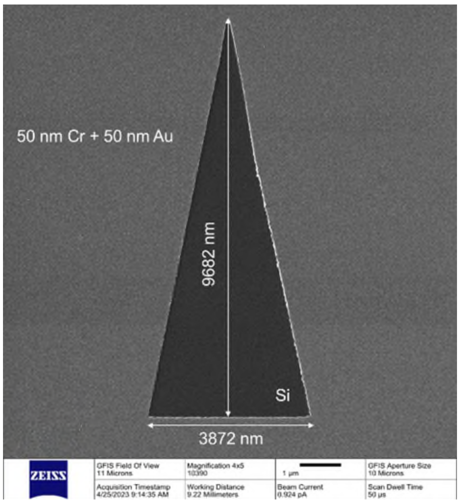

Fig. R12 SEM image of a triangle Si/Cr-Au sample fabricated by typical EBL process. In brief, a thin layer of PMMA was used as the photoresist. The photoresist in the outer area of the triangle was removed by electron beam exposure and development. Then, 50 nm Cr and 50 nm Au were deposited by electron beam evaporation. The PMMA inside the triangle region was finally removed to form a triangle structure of exposed silicon.

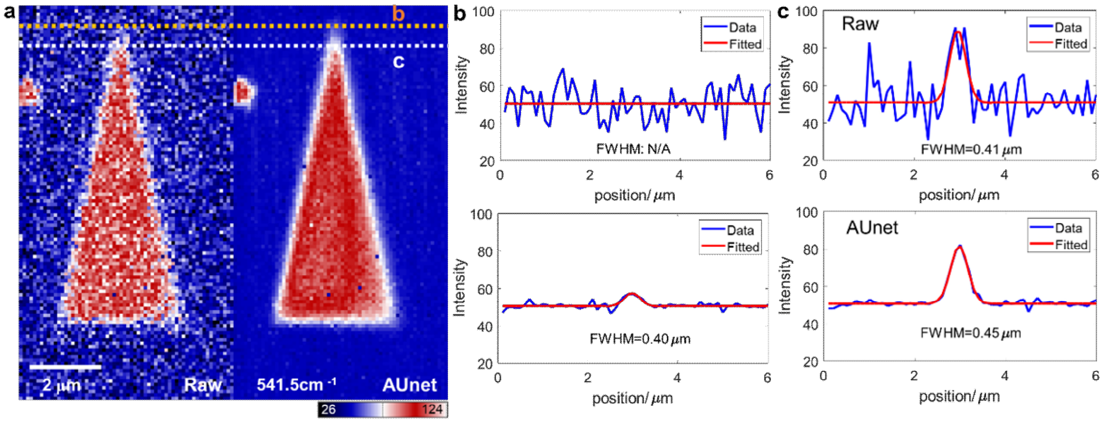

Fig. R13 Spatial resolution analysis of Raman images with and without denoising. (a) Raman images of the Si/Cr-Au triangle sample at 541.5 cm^{-1}, chosen for a better comparison of spatial resolution between low and high SNR Raman images. (b-c) Intensity profiles along the orange and white dotted lines in (a) and the corresponding Gaussian fitting results of Raw and AUnet data. In case (b) (at the tip of the triangle), the FWHM analysis is unavailable for the Raw data and the AUnet data yields a 400 nm FWHM which is quite close to the calculated theory spatial resolution. In case (c), the raw data exhibits a smaller FWHM compared to AUnet due to the presence of noise as shown in the simulation result in **Fig. R10**. Instrument: Raman-11. Objective: 100x, NA = 0.9. Laser: 532 nm.

(3) Motivation of deep learning approach

The method the authors suggest appears to be a more complicated version of Fourier filtering where knowledge about the instrument specific response is used to selectively modify the amplitude of certain frequencies in the Fourier transformed spectrum of a given sample. Couldn't such a correction be performed without the need for any deep learning? I suggest the authors try to selectively dampen the relevant frequencies in a measured spectrum according to the instrument

specific response (as shown in FigS1g-h) and compare the result to the deep learning-based approach. Which other factors motivated the use of deep learning as opposed to such a conceptionally more simplistic approach?

Re: We thank the reviewer for this comment. We should point out that the instrument responses in the pixel-spatial frequency domain as we show in **Fig. S1-g, h** are averaged over 10,000 instrument noise spectra. For a single Raman spectrum, the noise response fluctuates around the average value. Indeed, a notch filer can be used to remove specific pixel-spatial frequency components in the signals. However, the amplitudes of noise components at specific frequencies vary in different spectra. As a result, it is difficult to estimate the ratio of the amplitudes of a specific pixel-spatial frequency that should be deducted, as this may also remove the signal components. Complicated parameters tuning is required when using such methods to do noise reduction, which will introduce sample and user bias. Such limitations can be efficiently mitigated using deep learning, due to its end-to-end nature. Therefore, deep learning is a better alternate than the traditional frequency filters.

(4) Clarifications regarding the neural networks
- Networks were trained for differing epoch numbers (200, 100, 2000). What was the reason for that and what was the termination criterion?

Re: We thank the reviewer for this comment. There are several common stop criteria used in neural network training:
1.Maximum number of epochs. The training process can be stopped after a predetermined number of epochs.
2.Validation loss criterion. Training can be stopped if the validation loss function between consecutive epochs falls below the threshold.
3.Early stopping. The training process is stopped if the validation loss does not improve over a certain number of consecutive epochs, indicating that the model has reached a point of diminishing returns.
The choice of stop criterion depends on the specific problem, dataset, and network architecture, etc. In our approach, we experimentally evaluated the maxima epochs for each task combining the training dataset size, training and validation loss change. Part of the iteration steps-loss curves of the networks are shown in **Fig. R14**. The train and valid loss curves show that the networks converge very well.

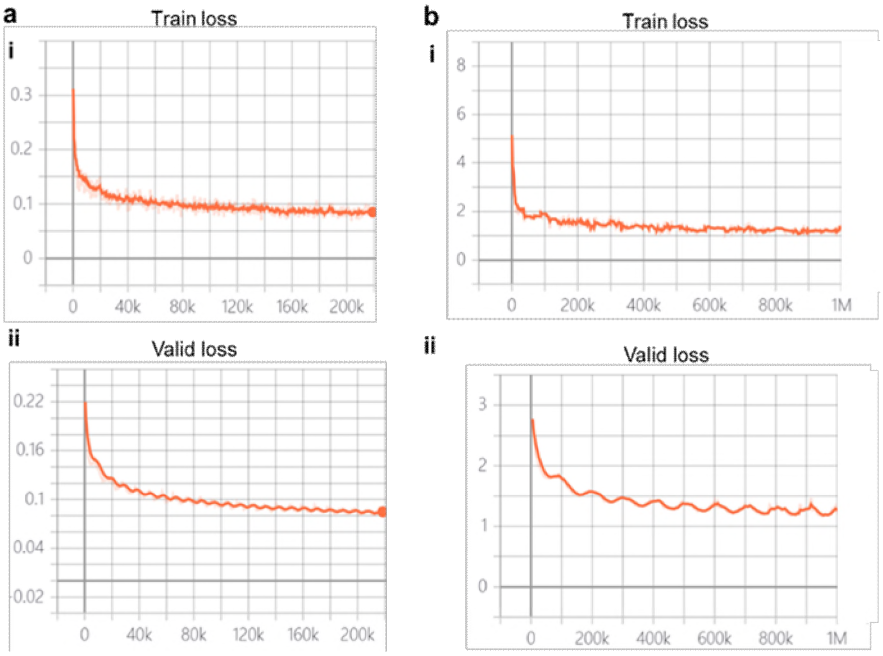

Fig. R14 Iteration steps-loss curves of AUnet training. (a) LabRAM HR-Evolution instrument. (b) Raman-11 instrument. The sinusoidal shape of the valid loss is due to the cosine annealing learning rate tuning method we adopted in the training. This learning rate tuning method can help the network escape from the local optimal point.

- Why was the GT generated differently for the CSL approach than the 'NL' approach? Wouldn't it be better for comparability to use the same method for CSL as for 'NL'? It appears that the CSL approach is limited towards unseen samples because of the selective GT data.

Re: We sincerely appreciate the reviewer's comment. The dataset is the primary component of the machine learning approaches due to their data-driven nature. The performance of the machine learning model is largely up to the quality of the training dataset. As we mentioned in the introduction part of the main text, one of the main differences between the "NL (noise learning)" and "CSL (conventional supervised learning)" method is the generation of the training dataset. The ground truth (GT) dataset generation in the CSL approaches typically involves direct measurements of Raman spectra of specific samples with long integration time [8,9], or through the averaging of multiple measurements taken from the same region of interest (ROI) [10]. The data generation method we used for the CSL model in our manuscript is similar to those references. We think the comparison method is fair in our current version. Therefore, we respectfully hold a different perspective from the reviewer regarding the suggestion of comparing the CSL approaches and NL approach using the same data generation method. Similar comparisons can be found in ref (*Nat Mach Intell* 3, 1071-1080 (2021).) [11].

- The 'NL' network was trained in the Fourier domain. It is not entirely clear from the manuscript if the CSL network was trained on the spectra, or their Fourier transform as well. If it wasn't trained in the Fourier domain: why and what would happen if it was done that way?

Re: We sincerely appreciate the reviewer's comment. The CSL network was trained on the spectra

domain. While it is possible to train the CSL network in the frequency domain as well, as stated previously, the limited training dataset would not contribute to any significant improvement to the network's generalizability, which is the key development of our NL network.

Changes: We have explained more clearly in the Methods section "Noise learning, conventional supervised learning and network training." that the CSL network was trained on the spectra domain

Minor points:

(1) The authors provide comparisons of their technique and two rather simplistic smoothing routines (Savitzky-Golay, Wavelet) that don't take external information (for example about the peak shape of Raman signals) into consideration. It would be interesting to see comparisons with more sophisticated state-of-the-art post-processing procedures.

Re: Thank you for bringing up this point. We genuinely value the reviewer's comment. To our knowledge, the SG and Wavelet methods are classical algorithms commonly used in noise reduction of Raman spectra. These methods are widely referenced and serve as benchmark approaches in many studies [8,9,12]. In light of this, we believe it would be valuable to include a comparison with the SG and Wavelet methods in our study. This will provide a comprehensive evaluation and allow for a fair assessment of the performance of our proposed approach in relation to other established methods. Comparison with more sophisticated state-of-the-art post-processing procedures could be interesting yet would not give us a fair judgement of the performance of our method. Therefore, we wish to limit the comparison to SG and Wavelet methods in the manuscript.

(2) Abbreviation 'PIC' not explained upon first mentioning (only in line l. 512 in the last paragraph).

Re: Thank you. It refers to phenyl isocyanide, the molecule that we used as the Raman probe. We have given its full name before abbreviation in the revised version.

(3) The Supporting Information movies should include a color bar and the intensity scaling should be kept the same between frames. As it is, the constant rescaling makes it difficult to judge bleaching, especially in the first few frames (see 405631_0_video_7196240_rn7x4x.avi) where bleaching should be strongest but individual frames appear virtually unchanged. The same is true for Fig. 4.

Re: We thank the reviewer for pointing out this question. We follow the suggestion of the reviewer and present the first 6 images of the fluorescence imaging of HeLa cell with high laser power in the same region (**Fig. R15-b**). It can be found that the mean intensity of the image dramatically reduces with increasing exposure time (frames, **Fig. R15-a**), which indicates photobleaching. However, the images would become completely dark in the next few frames, which makes it difficult to visualize. Therefore, we would like to present the normalized version of them, which can provide more image details for comparison. We have added normalized color bars to the Supporting Movies and Fig. 4 in the revised version (now **Fig. R16**).

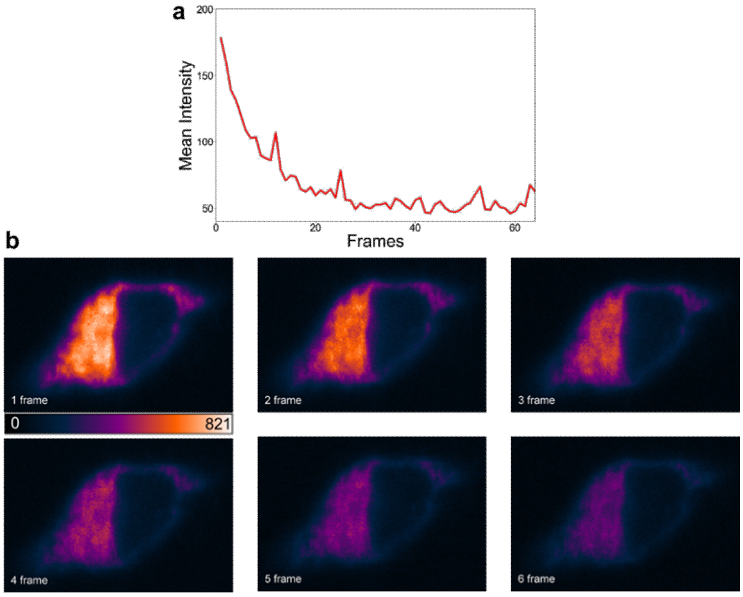

Fig. R15 (a) The mean fluorescence intensity curve of the HeLa cell. (b) The first 6 frames of fluorescence imaging of HeLa cell with high laser power. The intensity scale for each frame is the same to allow a better comparison of the photobleaching behavior.

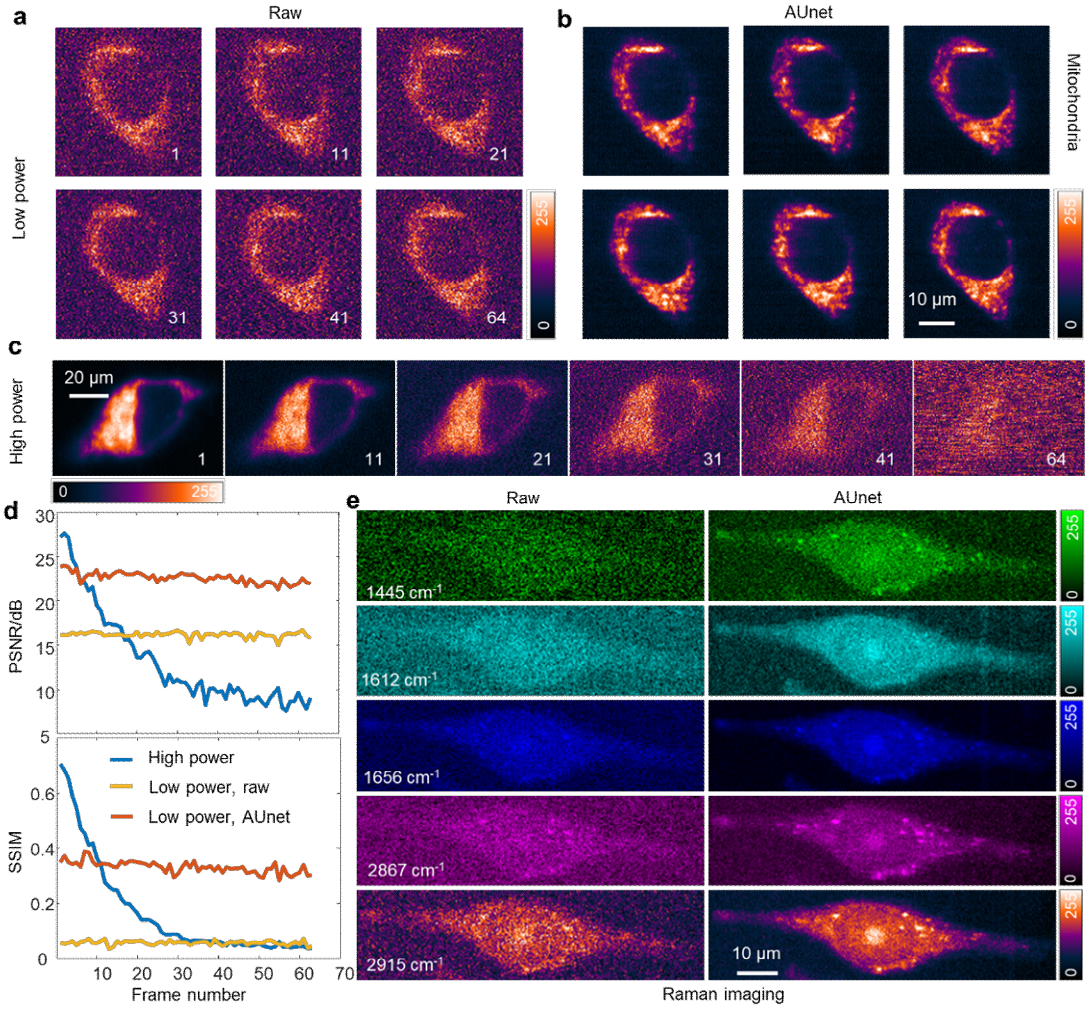

Fig. R16 Revised version of Fig.4 in the main text with normalized scale bar for all the images

(4) Throughout the manuscript the authors claim to have measured live cells (e.g.: l. 82) but the 'methods' section clearly mentions that the cells were fixed (and therefore killed) by 75% ethanol, 10 min. Please clarify.

Re: We sincerely appreciate the reviewer for pointing out this unclear expression. We indeed measured the live cells in the fluorescence imaging experiments in Fig. 4(a-c). While the cell is fixed by 75% ethanol in the Raman imaging experiment in Fig. 4(e).

Changes: We have revised this in the 'Method-Sample preparation' section accordingly.

The HeLa cells were seeded onto 35 mm dishes containing coverslips and cultured in DMEM (Yuanpei, Shanghai, China) supplemented with 10% fetal bovine serum, 1% non-essential amino acids, penicillin, and streptomycin. The cells were incubated in a humidified 5% CO2 incubator at 37°C for 24 hours. For labeling, the adherent cells on the coverslip were stained with LysoTracker Green DND-26, MitoTracker Green FM, and BODIPY FL PI(5)P. The staining procedure followed the protocols provided by the manufacturer (Thermo Fisher Scientific, USA) and lasted for 30 minutes. After staining, the cells were washed twice with DMEM (without phenol red). For fluorescence imaging experiments, the stained cells were cultured in an in-situ pool (Tokai Hit) to maintain a humidified atmosphere (5% CO_2, 95% air, 37 °C). For Raman imaging experiments, the cells were fixed in 75% ethanol for 10 minutes and then washed three times with sterile PBS. Subsequently, the cells were sealed on slides for Raman imaging.

(5) Figures 1e (GT vs. AUnet) and 2b, 2d (‚local mean' vs. 'NL') seem to suggest that the method proposed by the authors flattens the baseline towards the edges while such behavior is not visible in Figure 2f. Where does this behavior originate from? The CSL approach seems to respect the 'local mean' baseline characteristic.

Re: We appreciate the reviewer's comment. We have verified that the observed "local mean" baseline characteristic is a fixed response inherent to the instrument, resulting from the variation in the CCD efficiency of the edge pixels. This can be clearly observed in the mean Raman spectra of Au, Si, graphene, and WSe_2 samples measured using the same instrument (**Fig. R17**). When utilizing the training data generation method mentioned earlier to train the 'CSL' model, the model can learn this fixed response and, therefore, it exists in all CSL spectra. Fortunately, as the reviewer noted, this CCD response can be efficiently eliminated using the proposed 'NL' method. This is because such a 'drop' baseline has recognized as "background" and been removed from the instrumental noise (See **Fig. R1** for more details) before it is used to generate the training dataset.

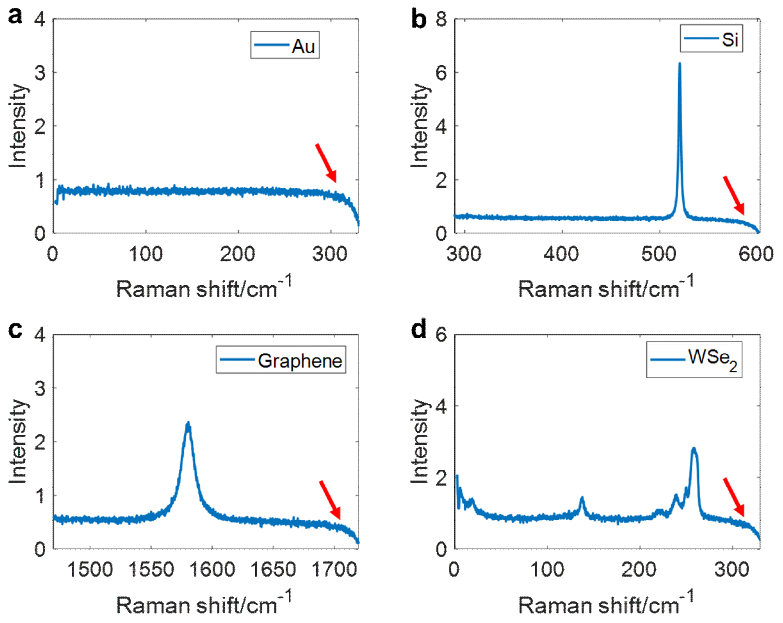

Fig. R17 Mean Raman spectra of (a) Au film, (b) Si wafer, (c) Graphene, and (d) WSe_2 measured using the Horiba LabRAM HR-Evolution instrument. The red arrows indicate the observed intensity drop in the high wavenumber end, attributed to the variation of the efficiency of the CCD pixels.

(6) The figure description for FigS1c is insufficient. It is unclear what the individual panels depict. The authors point towards the method section for more details, but clarification isn't given there either.

Re: Thank you for your comment. The description of the data processing pipeline, including Eq. (R6) and Eq. (R7), can be found in the 'Instrumental noise measurement' section of the Method.

Briefly, the multiple spectra of Au film are arranged as a two-dimensional matrix, which are then decomposed by the singular value decomposition (SVD) by:

$$S_{m\times n} = U_{m\times n}\Sigma_{m\times n}V_{m\times n}^T = [u_1 \quad \cdots \quad u_m] \cdot \begin{bmatrix} \Sigma_k & 0 \\ 0 & 0 \end{bmatrix} \cdot \begin{bmatrix} v_1^T \\ \vdots \\ v_n^T \end{bmatrix}, \ \Sigma_k = \begin{bmatrix} \sigma_1 & \cdots & 0 \\ \vdots & \ddots & \vdots \\ 0 & \cdots & \sigma_k \end{bmatrix} \quad \text{(R6)}$$

where m denotes number of data point in each Raman spectrum determined by the effective readout pixels of the CCD detector, n denotes the total number of the spectra in the matrix. After that, the instrumental noise can be obtained by removing the background components in the above equation. This can be done by manual inspection of each column vector of the left singular matrix $(u_1 \quad \cdots \quad u_m)$, or simply remove the first component $(\sigma_1 \cdot u_1 \cdot v_1^T)$ because it contributes the most majority of the background. By denoting J as the set of background components index, the instrumental noise can be calculated as follows:

$$Noise_{m\times n} = S_{m\times n} - \sum_J \sigma_j \cdot u_j \cdot v_j^T \quad \text{(R7)}$$

We think that this explanation is straightforward and can be easily understood.

Changes: To improve the readability and make the text clearer, we have changed "*see Methods in the main text for more details*" to "*see Methods - Instrumental noise measurement section in the main text for more details*" in the figure caption of Fig. S1c.

(7) Fig S1a-d and the corresponding part in the method section describe the removal of a global baseline drift from the 'instrumental noise' spectra recorded on gold. This step is not motivated in the text. My interpretation is that this is a gold-specific (i.e. not instrument-related), non-Raman contribution which should therefore not be taken into account in further steps. Is this correct? If so, this should be stated in the manuscript.

Re: We appreciate the reviewer for raising this question. Your understanding is correct. The motivation behind the removal of the 'baseline drift' is driven by two primary reasons.

First, as highlighted by the first reviewer, gold can exhibit luminescence when excited by visible light, resulting in the presence of photoluminescence (PL) contribution in the measured gold spectra. By eliminating the 'baseline drift', we can effectively separate the instrumental noise from the PL component.

Second, as demonstrated in **Fig. R17**, the instrument-specific offset caused by the variation of the grating scattering efficiency can contribute to the baseline. Removing the 'baseline drift' enables us to effectively eliminate this relatively stable non-Raman background.

Changes: We added more details about this in the 'Methods- Instrumental noise measurement' section of the main text.

The instrumental noise in Raman microscopy is indirectly extracted from the spectra obtained from a smooth Au film, which does not exhibit observable Raman peaks (Fig. S1). It is important to note that the Au film can still emit photoluminescence (PL) signals and reflect light, which need to be eliminated from the Au spectra. To this end, we propose the utilization of singular value decomposition (SVD) to separate this contribution from the instrumental noise. The spectra are organized as a two-dimensional matrix and subsequently decomposed using SVD according to the following equation:……

(8) In the method section, a value of ρ=0.6785 is specified for the pseudo-Voigt function. Where does this value originate from? The high numerical precision given hints at some thought being put into this value. It only says, 'These parameters are randomly extracted in a given range, which should be optimized on a specific Raman instrument' (l. 421ff). What range and what does 'randomly extracted' mean? How sensitive can the method proposed by the authors expected to be with regard to ρ? It would appear that this rather fundamental assumption about the line shape of Raman measurements in the specific device used by the authors might have a significant influence. The peak shape should – to a degree – depend on the device. Why was the same value used for both devices?

Re: The Voigt function is the convolution of Lorentzian and Gaussian functions. Here we use pseudo-Voigt function which is a linear combination of Lorentzian and Gaussian parts. The deduction of the ρ is as follows [13]:

$$\rho = 1.36603 * {}^{f_l}/_{f_v} - 0.47719 * \left({}^{f_l}/_{f_v}\right)^2 + 0.11116 * \left({}^{f_l}/_{f_v}\right)^3 \quad \text{(R8)}$$

Where f_l and f_v are the FWHMs of the Lorentz and pVoigt profiles, respectively. The f_v can be approximated as follows [14]:

$$f_v \approx 0.5346 * f_l + \sqrt{0.2166 * f_l^2 + f_g^2} \quad \text{(R9)}$$

Where f_g is the FWHM of the Gaussian profile. Here, for simplicity, we use the same FWHM value for f_g and f_l, and obtained the coefficient value of 0.6785. Therefore, the value of ρ is purely determined using the above equations without any other inputs.

As for the parameters for generating a Raman spectrum, the number of Raman peaks, FWHM and position for each peak are randomly extracted in a given range. For example, the number of Raman peaks was set in 1-10 range, the FWHMs was set from 5 to 200 cm^{-1}, and the peak position was set in the range of 1-1600 cm^{-1} when training the AUnet model for the Horiba instrument.

Based on our understanding, the peak shape of the GT Raman signal is determined by the vibrational mode of the molecule and is independent of the specific instrument used for measurement. Therefore, we think it is reasonable to utilize this method for generating the GT datasets for both instruments.

Changes: To make it clear, we have revised this in the Methods part of the main text. In the Methods section, we add "*The number of Raman peaks, FWHM and position for each peak are randomly extracted in a given range. For example, the number of Raman peaks was set in 1-10 range, the FWHMs was set from 5 to 200 cm^{-1}, and the peak position was set in the range of 1-1600 cm^{-1} when training the AUnet model for the Horiba instrument.*"

(9) In l. 156ff it says, 'In contrast, the AUnet leads to spectra with significantly improved SNR, which well restores the GT spectra, even if the SNR of the raw spectra was very low or negative'. How could there be a negative SNR? Is this just a numerical problem?

Re: This is because we use a different definition of signal-to-noise (SNR) metric in the unit of decibel (dB) including a log function, which is commonly used in the signal processing field. It can be calculated as follows:

$$SNR = 10 * \lg {}^{P_{signal}}/_{P_{noise}} \quad \text{(R10)}$$

The negative SNR indicates that the signal power is smaller than that of the noise.

Changes: We added an elaboration "*i.e., the signal power is smaller than that of the noise (Eq. 10)*" after the negative SNR expression, which would make the text clearer.

(10) Fig S4b lacks a color bar. Are all data displayed on the same scale? If so, why is there a drop in mean intensities for example in the 518.7 cm-1, 0.02 mW vs. 0.05 mW measurements?

Re: We thank the reviewer for this comment. All the images in **Fig. S5b** are normalized to (0-1) scale. The intensity of the 0.02 mW measurement is almost at the noise level, as can be seen in panel c. When comparing the Raw and AUnet images at the same power, it is the reduction of noise that makes the image look different. However, all images are in the color range of blue (0) to red (1).

Changes: We have added a color bar for each image panel. The revised version of **Fig. S5** is shown below.

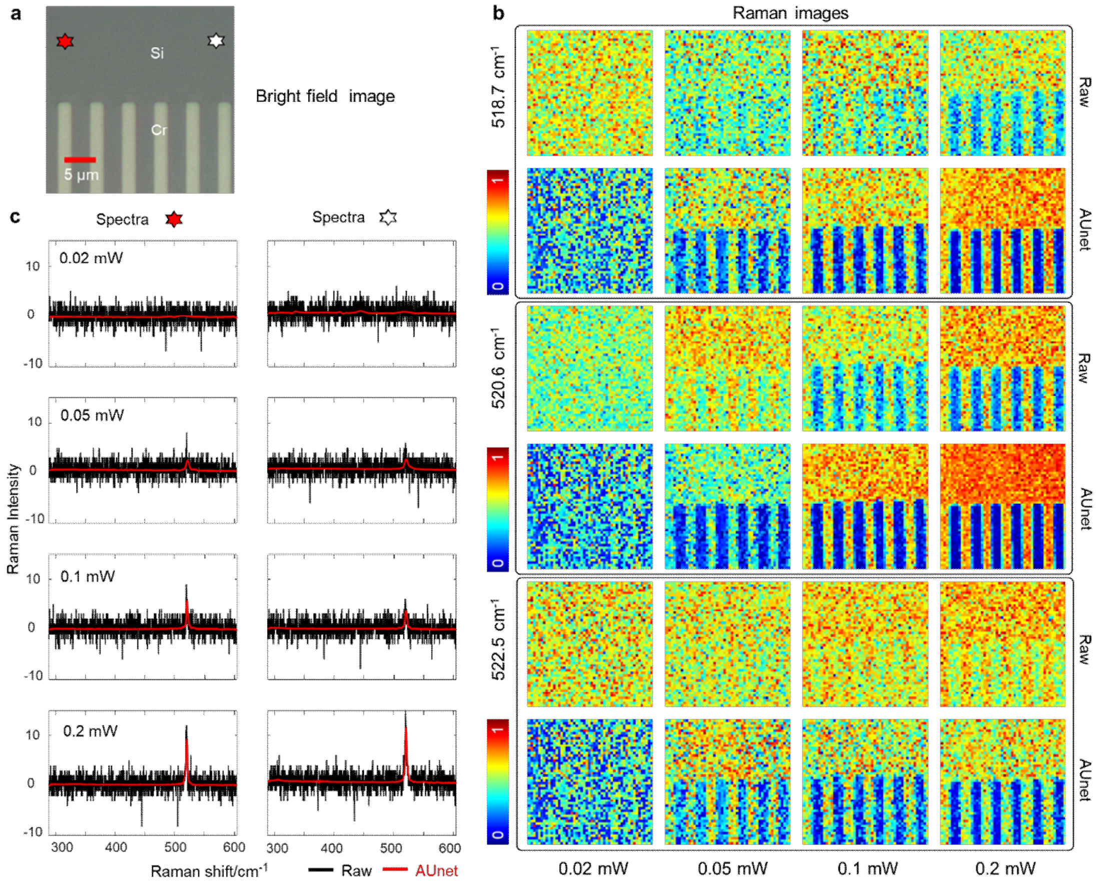

Fig. R18 Revised version of Fig. S5 in the supplementary information.

(11) The authors state that there is potential for the 'application of NL approach for the downstream biological analysis, even possibly for real time analysis' (l. 354). How long does the processing actually take on a modern computer? Milliseconds? Seconds? Minutes?

Re: The running time is about 6.4 ms/per spectrum, which is sufficiently fast for many bio-imaging applications. Computer settings: GeForce GTX1050 GPU, Intel(R) Core (TM)i7-7700HQ CPU, Software: Python (Version: 3.8.3), PyTorch (Version:1.5.0), IDE: PyCharm (Version: 2022.1.3). Operation system: Windows10.

(12) Very minor point: Assuming linear axis scaling, figure 1a (left) seems to suggest a linear

relationship in Raman microscopy between any two of the parameters speed, resolution, and SNR when the third is set to a target value. Though it is clear that this is supposed to be simplified schematic, it should be noted that this is not true. Accordingly, the proposed benefits of deep learning (right) seem overly optimistic and seem to imply a known and general qualitative relation between the parameters in question upon the application of the method proposed by the authors ('In this article, we present a noise learning (NL) method that can largely expand the generalizability of deep learning, allowing accurate removal of the instrumental noise, and improve the sensitivity, spatial and temporal resolution of hyperspectral Raman microscopy and nanoscopy (Fig. 1a).'). This strikes me as far too general, and I suggest removing the graphic.

Re: We appreciate the reviewer's comment on the figure. The purpose of this schematic was to provide a simplified representation of the parameters and their potential interplay. We did not intend to imply a known and general qualitative relation between these parameters upon the application of our proposed NL method. We apologize if this was not clear.

Changes: We followed the suggestion and removed the scheme and rearranged Figure 1 as follows.

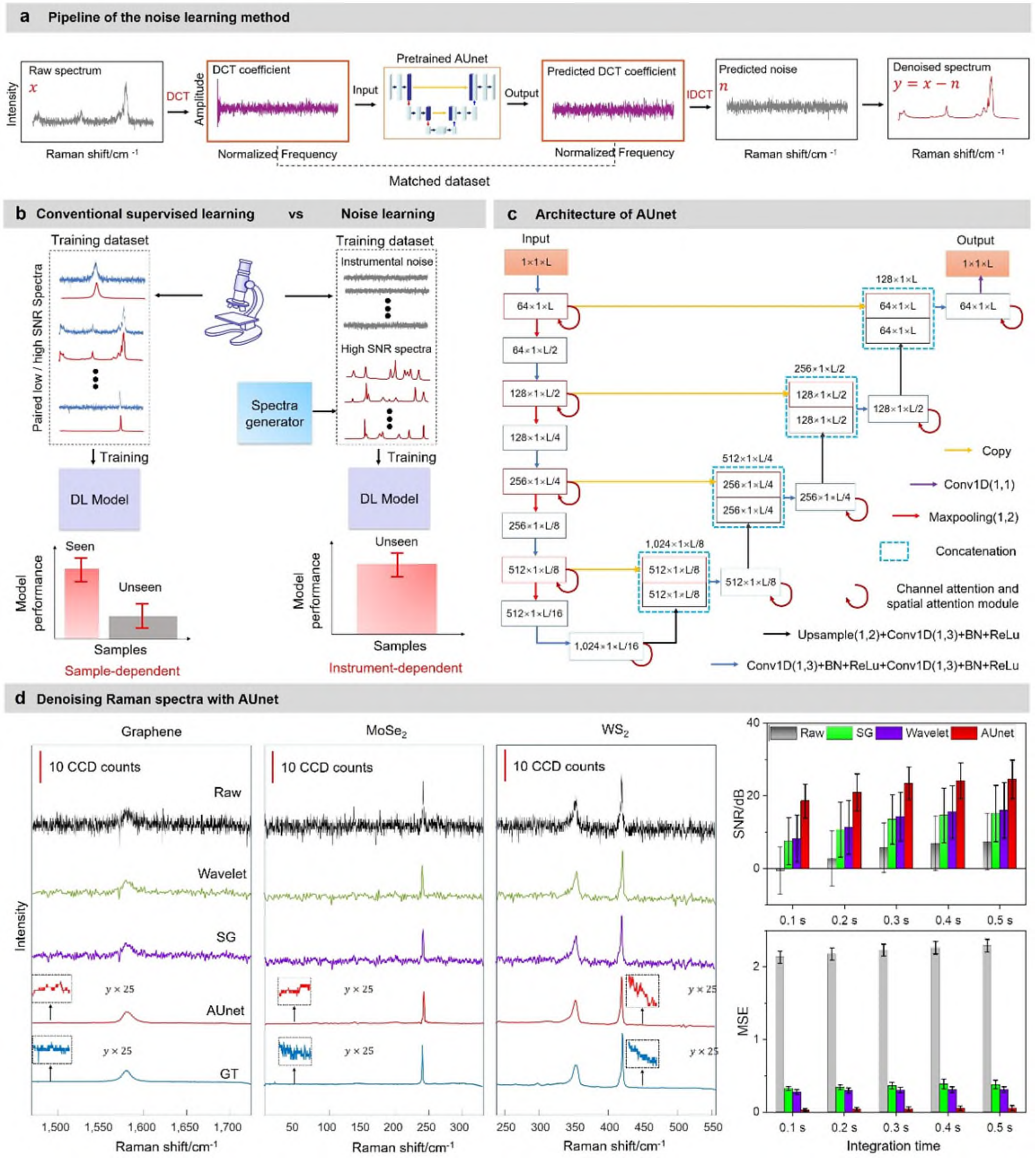

Fig. R19 Revised version of Fig.1 in the main text.

(13) For true noise, different integration times change the composition of said noise (e.g. shot noise vs. read-out noise vs. dark noise) and those contributions generally follow different distributions. Assuming that the frequency-stable unwanted signal contributions are not also phase-stable (in which case the authors' method would practically amount to a true background subtraction), it is unclear why the model had to be trained on datasets with different integration times. Expectedly for the type of instrument-specific signal under discussion, the normalized amplitudes and frequencies (Fig. S1g-h) do not change significantly with different integration times. Why did the authors deem it necessary to include this additional variable?

Re: We thank the reviewer for the comment. The motivation of including the instruments noise with different integration time into the training datasets stems from the observation of the noise power spectra changes with the integration time (**Fig. R20-a**). As can be seen, the amplitude of the noise

power spectrum increases with the increasing integration time. The amplitudes of certain peaks also vary with different integration time (**Fig. R20-b**). When a model is trained using datasets that encompass different integration times, it can learn to account for the changing characteristics of the noise with respect to integration time. This can make the model more robust and accurate when making predictions or analyzing data that may have been acquired with different integration times.

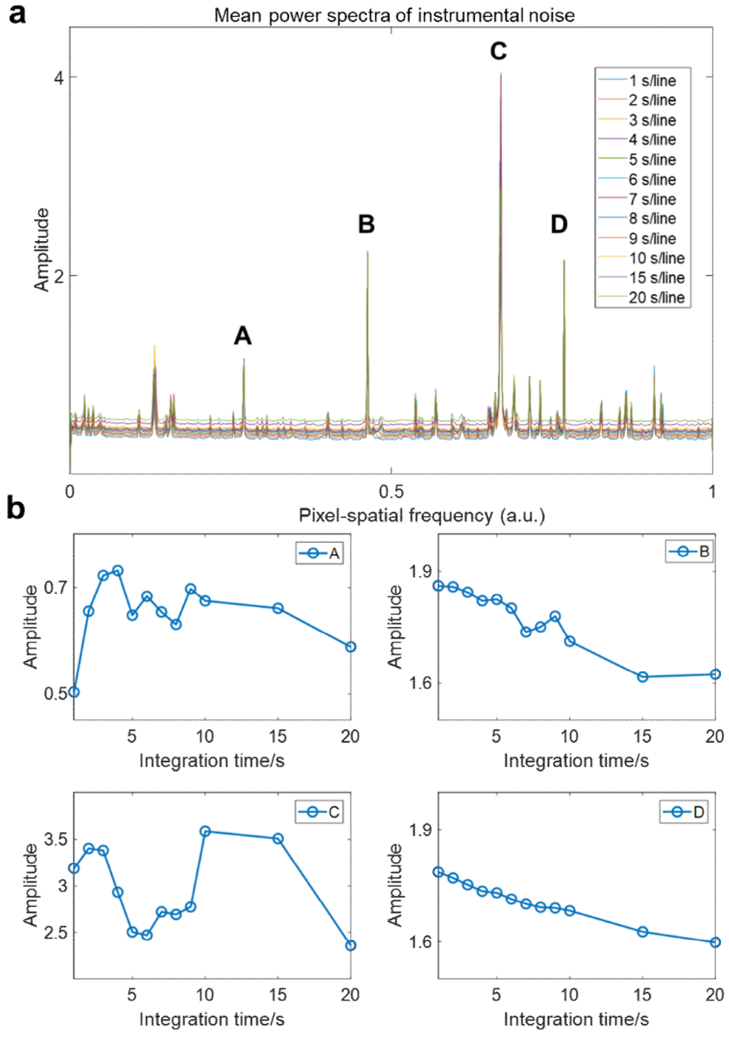

Fig. R20 (a) Mean power spectra of the instrumental noise extracted from 10,000 Au film Raman spectra with different integration time (line scan: 1-20 s/line). Instrument: Raman-11. Objective: 50×, NA=0.45. Laser: 532 nm. (b) The amplitude-integration time curves of the 4 frequency components marked by A, B, C and D in (a).

References:

[4] Yin, H. et al. Nanometre-scale spectroscopic visualization of catalytic sites during a hydrogenation reaction on a Pd/Au bimetallic catalyst. *Nat. Catal,* 3, 834-842 (2020).

[5] Wang, X., Huang, S.-C., Hu, S., Yan, S. & Ren, B. Fundamental understanding and applications of plasmon-enhanced Raman spectroscopy. *Nat. Rev. Phys,* 2, 253-271 (2020).

[6] Zhong, J.H. et al. Probing the electronic and catalytic properties of a bimetallic surface with 3 nm resolution. *Nat Nanotechnol* 12, 132-136 (2017).

[7] Zhang, R. et al. Chemical mapping of a single molecule by plasmon-enhanced Raman scattering.

Nature 498, 82-86 (2013).
[8] Machado, L.R.P. et al. Deep-learning-based denoising approach to enhance Raman spectroscopy in mass-produced graphene. *J Raman Spectrosc* 53, 863-871 (2022).
[9] Horgan, C.C. et al. High-Throughput Molecular Imaging via Deep-Learning-Enabled Raman Spectroscopy. *Anal Chem* 93, 15850–15860 (2021).
[10] Lin, H. et al. Microsecond fingerprint stimulated Raman spectroscopic imaging by ultrafast tuning and spatial-spectral learning. *Nat Commun* 12, 3052 (2021).
[11] Sekh, A.A. et al. Physics-based machine learning for subcellular segmentation in living cells. *Nat Mach Intell* 3, 1071-1080 (2021).
[12] Barton, S., Alakkari, S., O'Dwyer, K., Ward, T. & Hennelly, B. Convolution Network with Custom Loss Function for the Denoising of Low SNR Raman Spectra. *Sensors* 21 (2021).
[13] da T, Ando M, Toraya H. Extended pseudo-Voigt function for approximating the Voigt profile. *J Appl Cryst*. (2000). 33, 1311-1316.
[14] Olivero, J. J.; R. L. Longbothum. Empirical fits to the Voigt line width: A brief review. *Journal of Quantitative Spectroscopy and Radiative Transfer*. 17 (2): 233–236.

Reviewer #3 (Remarks to the Author):

The authors have proposed a deep learning based method to learn noise of the instruments such as Raman microscope (LabRAM) and Raman line scan instrument (Raman-11). The concept is training the 1D U-Net with attention module with instrument noise and high SNR spectra using the spectral generator. The spectrum is processed into DCT coefficient and given as input to 1-D U-NET+attention module and predicted output is obtained by inverse DCT. Now this predicted noise is subtracted from the raw data to obtain the denoised data.

Re: We thank the reviewer for the summary and all the comments below.

The claims made in the paper is that 10 folds enhancement over SNR, 150 folds improvement over MSE and 2 fold improvement on spatial resolution of 2.9 nm in air. Did author mean here spectral resolution and not the spatial resolution of 2.9 nm?

Re: Thank you for your comment. Yes, the 2.9 nm is the spatial resolution of tip-enhanced Raman spectroscopy (TERS), which represents the ability to distinguish the electronic and catalytic properties of the adjacent Au and Pd surface regions/sites in our sample. TERS is nanospectroscopy enabling the acquisition of molecular fingerprint information with a spatial resolution of 2-3 nm under ambient conditions [6] and even down to 1.5 Å under ultra-high vacuum and ultra-low temperature conditions [15].

In fact, the authors are losing spatial resolution by a significant amount due to 11x11 moving window average. In this case, why not use a low NA imaging setup that inherently covers a larger volume and therefore presents a better averaging anyway.

Re: Respectfully, we feel that the reviewer might have misunderstood the denoising network and **Fig. S3** in the supporting information. The reviewer might mean that the network receives 2- or 3-dimensional images as the input, which is not the case. Both for the NL (noise learning) and CSL (conventional supervised learning) network, the input and output are 1-dimensional data without any spatial information (**Fig. 1-a**). The hyperspectral microscopic and nanoscopic images presented in our manuscripts are denoised in the pixel-wise manner, each pixel (or each surface spatial site) contains a full spectrum.

The purpose of using 11x11 moving window average over the spatial dimension is to reduce the noise in the spectral domain, and the processed hyperspectral data cube is not directly used to train the network. Therefore, the moving window does not affect the spatial resolution.

I worry about the claim of authors regarding drift. I do understand that thermal drift refers to spectral drift in Raman spectrum, but since the authors target microscopy and nanoscopy data, they may want to consider the spatial drift of the sample as well. The spatial drift will result into mixing of two spectra at the boundaries between two materials (for examples Si and Cr in Fig. S4) and therefore simulating just the instrument's other forms of noise may not be sufficient.

Re: We did mean the spatial drift in TERS image due to the relative movement of tip with the sample

rather than the spectral drift. Such spatial drift will lead to the unwanted mixing of the signals from different surface sites, hampering us to obtain a reliable structure-spectra correlation. The spatial drift is a result of the thermal movement of all mechanical components including the mechanical strain of tip and sample holders, scanner of the scanning probe microscopy. It will thus distort the images particularly when the imaging time is long and the experiments are performed under ambient conditions. For microscopic imaging, the drift problem is less pronounced since the spatial resolution is on the order of a few hundreds of nanometers, whereas for nanoscopic imaging (like TERS), the spatial resolution is on the order of a few nanometers or less. It is the purpose of our study to develop the noise learning method so as to reduce the spectra acquisition time that is needed to acquire a TERS image, thus lifting the drawbacks of the spatial drift.

Changes: To make the text clearer, we have emphasized the "spatial drift" in the revised manuscript now.

At the same time, I wonder about the title containing the words microscopy and nanoscopy. Is the scope of paper nanoscale volumes or are the authors really meaning to use the spatial distribution measured in microscopy and nanoscopy modes. In some way and even if unintended by the authors, it seems that microscopy and nanoscopy terms are added either to bank upon the popularity of the terms or to avoid readership (and review) from spectroscopy community.

Re: We appreciate the reviewer's comments and concerns regarding the title of our paper. We apologize if it led to any confusion or unintended implications.

The use of the terms "microscopy" and "nanoscopy" in the title was intended to highlight the capability of our proposed method to enhance the hyperspectral Raman imaging at the microscale and nanoscale levels, as demonstrated in our experiments from microscopic imaging of various materials with conventional Raman microscope and nanoscopic imaging of a catalyst sample with high-resolution TERS experiment. It was not our intention to solely rely on the popularity of these terms or to exclude readership from the spectroscopy community and we rather addressed the increasing and expanding readership interested in hyperspectral images (with Raman microscopy and nanoscopy) from the spectra type datacube. As discussed above, in all these microscopic/nanoscopic images, each pixel (or each surface spatial site/point) contains a spectrum. In fact, our denoising method works directly and explicitly on those Raman and fluorescence spectra, and therefore, it is intended to be beneficial for the spectroscopy community. Our goal is to contribute to the field of spectroscopy and imaging techniques by addressing challenges related to noise reduction in hyperspectral imaging. We, therefore, hope our work can be seen by the spectroscopy community.

It does seem that the principles of denoising used in the paper would apply to spectroscopic measurements and microscopy/nanoscopy only present an example situation where SNR may be high. On the same note, when targeting the microscopy and nanoscopy community through the selected title, it is misleading to use high-resolution and claim 2.9 nm spatial resolution enhancement where it seems that the authors mean high spectral-resolution and spectral resolution enhancement of 2.9 nm. Also, is it 2.9 nm resolution or precision? It is likely that I have misunderstood the terminology especially coming from microscopy rather than Raman

imaging/spectroscopy background. Nonetheless, the targeted readers are likely to be presented with the same challenges as me. Therefore, the authors need to carefully delineate and if necessary explain the terminology and title in such a way that a general microscopy reader is not confused.

Re: Probably it is due to our unclear language that led to the misunderstanding of the review. As has been replied above, we really meant the 2.9 nm spatial resolution in Raman nanoscopy rather than the spectral resolution. The virtue of current method is that it can directly work on the spectra (Raman, fluorescence, *etc.*) measured from a Raman or IR or fluorescence microscopy and nanoscopy to generate hyperspectral images, as far as the instruments are equipped with a scanning stage. Therefore, it is necessary to include "hyperspectral" in the title to emphasize the rich spectral information contained in the microscopic/nanoscopic images.

Instead of high SNR spectra, our method is targeted for the sample that can only generate low SNR spectra, such as biological samples where high laser power should be avoided, or the minute signals from a very small surface area like in TERS or nano-IR. These low SNR signal can be improved with our noise learning method.

I also find that the novelty of the paper is weak – the use of U-Net and Unet+attention for denoising is plenty both in computer vision and signal processing.

a- Fan, C. M., Liu, T. J., & Liu, K. H. (2022, May). SUNet: swin transformer UNet for image denoising. In 2022 IEEE International Symposium on Circuits and Systems (ISCAS) (pp. 2333-2337). IEEE.

b- Zhang, B., Li, M., Zhou, C., Yang, Q., Wang, L., & Yuan, B. (2022, December). Endoscopic Image Denoising Algorithm Based on Spatial Attention UNet. In Journal of Physics: Conference Series (Vol. 2400, No. 1, p. 012026). IOP Publishing.

c- Qiao, Z., & Du, C. (2022). RAD-UNet: a Residual, Attention-Based, Dense UNet for CT Sparse Reconstruction. Journal of Digital Imaging, 35(6), 1748-1758.

Even Attention+UnEt+DCT has been explored by - Zhang, J., Niu, Y., Shangguan, Z., Gong, W., & Cheng, Y. (2023). A novel denoising method for CT images based on U-net and multi-attention. Computers in Biology and Medicine, 152, 106387. Simulation-supervised learning (which the authors refer to as physics-based learning) is also not unseen.

Based on these observations, it appears to me that the paper is not suitable for publication in Nature Communications.

Re: We agree that U-Net and variants have been widely used in computer vision and signal processing for denoising tasks, as evidenced by the references provided. However, it is a common practice to adopt the most advanced techniques developed in computer science to other fields. In this work, we for the first time are able to extract characteristic noise patterns of Raman instruments by using AUnet. This is conceptually different from any previous work. By leveraging deep learning techniques, we have successfully learned the noise distribution and developed a highly versatile and high-performance denoising method. Importantly, our approach is not limited to Raman spectroscopy but can be generally extended to broader spectroscopy instruments, such as fluorescence, infrared spectroscopy (IR), and nano-IR. By understanding and addressing the noise patterns specific to different spectroscopic techniques, we aim to contribute to the advancement of denoising methods that can enhance the performance and reliability of a wide range of spectroscopy

instruments.

We believe that the insights gained from our study and the development of this generalizable deep learning approach for noise reduction have significant implications for the spectroscopy community and warrant consideration for publication in a respected journal such as *Nature Communications*.

References:

[6] Zhong, J.H. et al. Probing the electronic and catalytic properties of a bimetallic surface with 3 nm resolution. *Nat Nanotechnol* 12, 132-136 (2017).

[15] Zhang, Y. et al. Visually constructing the chemical structure of a single molecule by scanning Raman picoscopy. *National Science Review* 6, 1169-1175 (2019).

Reviewer #1 (Remarks to the Author):

Firstly, the reviewer would like to express gratitude for the authors' efforts in addressing the comments previously mentioned. Nevertheless, after carefully reviewing the revisions, a few areas may require further clarification and improvement.

1. The authors have highlighted that the reviewer is dealing with a scenario where the fluctuation from shot noise by the signal exceeds that by dark counts and instrumentation noise, which is incorrect. It is crucial to note that the shot noise of the signal can notably deteriorate the SNR under low signal conditions. Thus, it becomes essential to consider the influence of the shot noise emerging from the background signal. Typically, when the shot noise of a signal surpasses other noises, the signal becomes more prominent, making the SNR sufficiently high to disregard other noise sources often.

2. The method the authors have presented in Fig.R1 for negating the effects of the gold raises some concerns. While the SVD does provide orthogonal vectors, it does not necessarily guarantee an accurate representation of the material information and may inadvertently encompass instrumental background. Simply omitting the vector one perceives as background might not be scientifically justifiable.

3. There is a need for more precise articulation regarding the specific types of noise the authors are addressing. In this context, instrumental noise can be synonymous with shot noise stemming from the background signal. However, even after eliminating this instrumental noise, the sample-dependent noise, attributed to shot noise by the signal, should remain unaffected. Consequently, the results appear unusually smooth, suggesting that the sample-dependent noise might also have been removed.

From the reviewer's understanding, in the proposed method, the machine likely learns two primary aspects:

a. The contour of the instrumental background. While this is not noise per se, the associated noise is statistically linked to the square root of the background. It is vital to differentiate between noise and background in the manuscript.

b. The machine's ability to remove the shot noise, regardless of its origin (signal or background), resulting in the smoothed data. Given the background learning process, this outcome seems feasible since the noise generation mechanism is not source-specific.

The essence of the method appears to be the learning of the instrumental background (distinct from noise) and simultaneously estimating a high-SNR spectrum shape. The reviewer kindly suggests revisiting and revising the manuscript, placing a particular emphasis on differentiating between the instrumental background and the two noise types: one from the signal and the other from the instrumental background.

Reviewer #2 (Remarks to the Author):

I would like to thank the authors very much for their very accurate and comprehensive responses to my comments and questions. The authors answered all of these in a convincing manner and resolved my criticisms to my complete satisfaction. I recommend acceptance of the revised manuscript in its present form.

Reviewer #3 (Remarks to the Author):

The authors have satisfactorily addressed my concerns, and clarified the previously unclear points. I will appreciate if the authors include such clarifying explanations in the manuscript as well (some

has been incorporated already), so that readers not from Raman background landing on the paper from microscopy/nanoscopy perspective have an easier time understanding and appreciating the paper.

Blue: Reviewers’ comments.
Black: Our responses.
Red: Revisions we made.

Dear Reviewers,

Thank you all for your valuable feedback and comments. We appreciate that you are satisfied with our revision. Below we address the remaining questions.

Thank you for reconsidering our revised manuscript.

Sincerely,
Bin Ren (on behalf of all authors).

Reviewer #1 (Remarks to the Author):

Firstly, the reviewer would like to express gratitude for the authors' efforts in addressing the comments previously mentioned. Nevertheless, after carefully reviewing the revisions, a few areas may require further clarification and improvement.

Re: We thank the reviewer for all the comments below.

1. The authors have highlighted that the reviewer is dealing with a scenario where the fluctuation from shot noise by the signal exceeds that by dark counts and instrumentation noise, which is incorrect. It is crucial to note that the shot noise of the signal can notably deteriorate the SNR under low signal conditions. Thus, it becomes essential to consider the influence of the shot noise emerging from the background signal. Typically, when the shot noise of a signal surpasses other noises, the signal becomes more prominent, making the SNR sufficiently high to disregard other noise sources often.

Re: We appreciate the reviewer's comment. We generally agree with the reviewer's point that the signal shot noise can also deteriorate the SNR of the Raman spectra. While the instrumental shot noise remains stable, the signal shot noise is proportional to the square root of the photon number. Therefore, with the reduced signal photon number, the weight of the signal shot noise will decrease and the weight of the instrumental shot noise will increase. Predictably, the instrumental shot noise is the dominant noise source when the signal photon number is sufficiently small. To validate this, we measured a batch of Si and Au spectra, and analyzed their signal shot noise. The average spectra of Au and Si are shown in **Fig. R1(a)**. The measured signal shot noises of Au and Si spectra are composed of several parts and can be written as Eq. R1 and Eq. R2, respectively.

$$\mathrm{SN(Au)} = \mathrm{N}_{pl} + \mathrm{N}_{bg} + \mathrm{N}_{dark} + \mathrm{N}_{readout} \quad \text{(R1)}$$
$$\mathrm{SN(Si)} = \mathrm{N}_{signal} + \mathrm{N}_{bg} + \mathrm{N}_{dark} + \mathrm{N}_{readout} \quad \text{(R2)}$$

Where N_{bg} denotes the shot noise arising from the background of the instrument, N_{dark} and $\mathrm{N}_{readout}$ are shot noise of the CCD detector, N_{signal} denotes the shot noise of the Raman signal, N_{pl} denotes the shot noise of the photoluminescence signal. Among them, N_{bg}, N_{pl} and N_{signal} also contain the contribution from laser fluctuation. The instrumental shot noise includes N_{bg}, N_{dark}, and $\mathrm{N}_{readout}$.

The detail steps for analyzing the shot noise are as follow:

1. We first measured the spectra of Au and Si substrate on the same position for 100 times in the line scan mode of Raman-11 instrument. Each laser line generates 400 spectra. The total number of spectra is thus 40,000.
2. The averaged spectrum of all these measured spectra is then taken as the low noise background.
3. The overall shot noise can be obtained by subtracting the background from the measured spectra.
4. The shot noise is taken as the standard deviation of the noise spectra using Eq. R3.

$$SD = \sqrt{\frac{1}{N-1}\sum_{i=1}^{N}(x_i-\mu)^2};\ \mu = \frac{1}{N}\sum_{i=1}^{N}x_i \quad \text{(R3)}$$

a Mean spectra of Au and Si, sample = 40,000

c

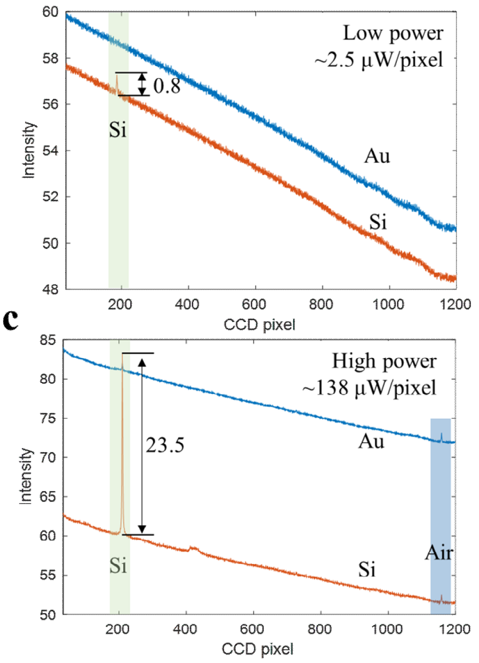

b Standard deviation, sample = 40,000

Low power, 2.5 μW/pixel

	Au	Si
Si band	8.88	8.84
Overall Exclude Si band	8.87	8.85

d

High power, 138 μW/pixel

	Au	Si
Si band	9.01	8.96
Overall Exclude Si band	9.02	8.97

Fig. R1 (a, c) Mean spectra of the Au and Si measured with different laser power by Raman-11 instrumnet using line-scan mode at the same positoin for 100 times repetive measurement, each laser line consists of 400 spectra. Laser = 532 nm, 50× air dry objective (NA=0.45), integration time: 0.5 s/line.These spectra are taken as the background and subtracted from the raw spectra to extract the noise. (b, d) The standard deviation calculated for the spectra region within the Si Raman band highlighted by light green in (a,c), and the overall spectral range excluding the Si band.

We first calculated the shot noise of Si and Au spectra within the Si Raman band region highlighted by the light green background as shown in **Fig. R1(a)**. The shot noise of Si and Au spectra are quite similar (~8.8 CCD counts) within the Si Raman signal band under moderate laser power (2.5 μW/pixel in line-scan mode) condition. Interestingly, the shot noise in the overall spectral range is also the same as that of the spectral region of the Si Raman band (**Fig. R1(b)**). We further increased the laser power (138 μW/pixel in line-scan mode) and used the same method to calculate the shot noise to see whether the increased Raman signal brings significant change or not. The results show that the standard deviation of both Au and Si increased slightly from ~8.8 to ~9.0 CCD counts. Although the maxima Raman signal intensity of the Si obviously increased from 0.8 to 23.5 (**Fig. R1(a, c)**), the standard deviation of the Si band remains stable compared with that of the full spectral range (8.96 vs 8.97, **Fig. R1(d)**). In view of these observations, we confirm that the shot noise originating from the Raman signal is low compared to the instrumental shot noise and can be reasonably disregarded in weak signal conditions of our applications.

2. The method the authors have presented in Fig.R1 for negating the effects of the gold raises some concerns. While the SVD does provide orthogonal vectors, it does not necessarily guarantee an accurate representation of the material information and may inadvertently encompass instrumental

background. Simply omitting the vector one perceives as background might not be scientifically justifiable.

Re: We appreciate the reviewer for the comment. Yes, it is true that the SVD cannot guarantee the isolation of signal from material (including Raman and PL) and background (or baseline, distinct from the shot noise) of the instrument's devices (such as CCD and laser). Probably, the reviewer might be confused by the unclear expression of our previous revision regarding this question. The goal of using SVD method is to separate the *instrumental noise* from the *background* and the *photoluminescent (PL) signal* originating from Au as shown in **Fig. R2** and **Fig. S1** of the supplementary information, and to obtain the pure instrumental noise with a flat baseline for further learning process.

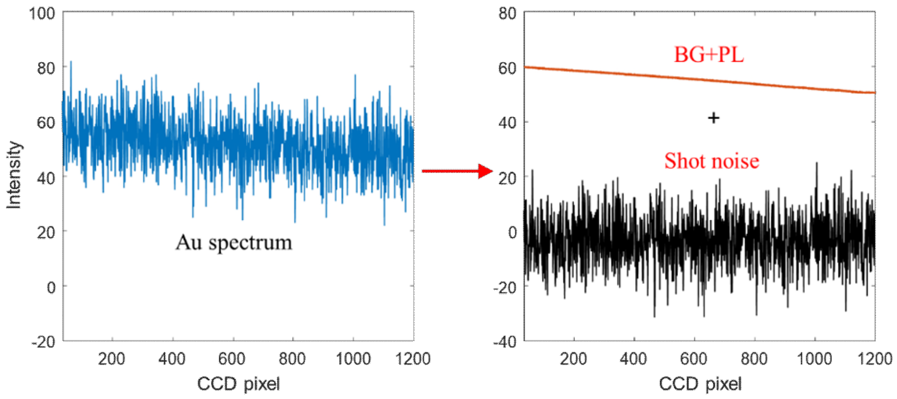

Fig. R2 The Au spectrum (left panel) can be decomposed to background (BG) plus photoluminenscent (PL) (right panel, red curve) and shot noise (black curve, Eq. R1). The shot noise is used for further analysis.

3. There is a need for more precise articulation regarding the specific types of noise the authors are addressing. In this context, instrumental noise can be synonymous with shot noise stemming from the background signal. However, even after eliminating this instrumental noise, the sample-dependent noise, attributed to shot noise by the signal, should remain unaffected. Consequently, the results appear unusually smooth, suggesting that the sample-dependent noise might also have been removed.

Re: We appreciate the reviewer for the comment. Yes, the reviewer is correct that our method is dedicated to removing the <u>shot noises arising from the background and instrumental devices</u> such as the CCD, laser and other optic elements, which are the dominant noise sources in our application scenario as we discussed in the response of the reviewer's first question. To answer the reviewer's concern, we also measured the Au spectra with different laser power (**Fig. R3**), and calculated the corresponding shot noise with the above method presented in **Fig. R1**. Interestingly, the standard deviation is 8.84 in the absence of laser irradiation (0 µW, $N_{pl} = 0$), meaning the CCD (dark current, readout noise) and background shot noise serve as the primary noise source in this scenario (**Fig. R3a**). It only slightly increased to 9.02 even with a laser power of 138 µW. Also, the noise fluctuation range seems stable with different laser powers as shown in **Fig. R3b**. We can try to estimate the relative contribution of different noise sources.

For the convenience of quantification, let $N_{ccd} = N_{dark} + N_{readout}$, then Eq. (R1) can be written as follows:

$$SN(Au) = N_{pl} + N_{bg} + N_{ccd} \tag{R4}$$

When the laser power is 0 ($N_{pl} = 0, \sigma = 8.84$, **Fig. R3a**), we have:

$$N_{bg} + N_{ccd} = 8.84 \tag{R5}$$

When the laser power is 138 μW (σ = 9.02, **Fig. R3a**) and suppose N_{ccd} remains stable, and the shot noise of background will increase proportional to its average intensity, we have:

$$N_{pl} + \sqrt{\frac{76.7}{52.3}} * N_{bg} + N_{ccd} = 9.02 \tag{R6}$$

where 76.7 denotes the average intensity of the mean Au spectrum measured with a laser power of 138 μW, and 52.3 denotes the average intensity at 0 μW. Combining Eq. (R5) and (R6) we estimate

$$N_{ccd} > 7.98,\ N_{bg} \leq 0.86,\quad N_{pl} < 0.18 \tag{R7}$$

Therefore, we can deduce that in the measured Au spectra with 138 μW laser power, the CCD contributes to the majority (>88.5%) of the shot noise, the background has a moderate contribution (<11.5%), and the shot noise coming from the PL signal makes a minor contribution (≪2%).

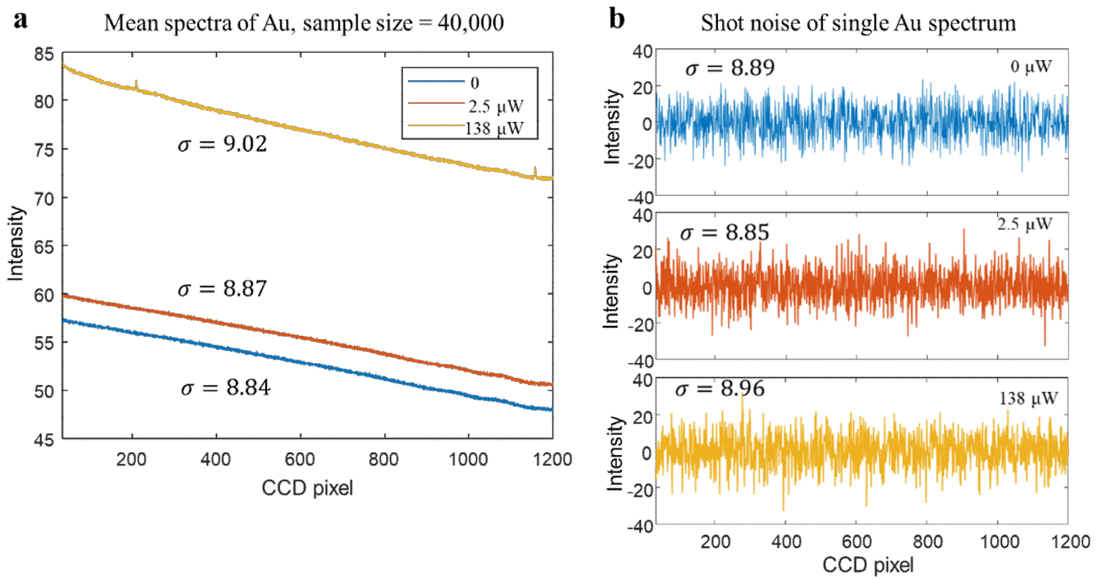

Fig. R3 (a) Mean Au spectra and standard deviation measured by Raman-11 using line-scan mode at the same positoin for 100 times repetive measurement with different laser powers. Each laser line consists of 400 spectra. Laser = 532 nm, 50× air dry objective (NA=0.45), integration time: 0.5 s/line. (b) Representative shot noise spectra of Au measured with different laser powers.

From the reviewer's understanding, in the proposed method, the machine likely learns two primary aspects:
a. The contour of the instrumental background. While this is not noise per se, the associated noise is statistically linked to the square root of the background. It is vital to differentiate between noise and background in the manuscript.
b. The machine's ability to remove the shot noise, regardless of its origin (signal or background), resulting in the smoothed data. Given the background learning process, this outcome seems feasible since the noise generation mechanism is not source-specific.
The essence of the method appears to be the learning of the instrumental background (distinct from noise) and simultaneously estimating a high-SNR spectrum shape. The reviewer kindly suggests revisiting and revising the manuscript, placing a particular emphasis on differentiating between the instrumental background and the two noise types: one from the signal and the other from the instrumental background.

Re: We appreciate the reviewer for the comment. We would like to emphasize that the proposed method aims at removing the shot noise arising from the instrumental devices. The reviewer is partially correct that the machine is able to remove the shot noise in spite of its origin, so that the sample-dependent shot noise can be also removed. However, we respectfully persist our point because the contribution of signal-dependent shot noise is too small to be considered in our application scenario (weak SNR). The CCD and instrument backgrounds contribute to most of the shot noise as we analyzed in **Fig. R3**.
In our processing pipeline, the background (include the PL signal) has been removed from the measured Au spectra, and the remaining instrumental shot noise is then used as the noise data to generate the training dataset. Hence, the AUnet model will not learn this specific background and pretend to output high SNR spectra similar to the training GT data. Part of the background may be preserved because the training GT data contains the generated baseline (to simulate the fluorescence background of the bio-sample) learned by the model.

Change: Following the reviewer’s suggestion, we add more description of the noise type we are addressing in the revised version.

In the ‘Results-Noise learning with physics-based dataset’ part we add:

Specifically, the shot noise arising from the detector (dark current and readout) and background of the instrument is the main source of the instrumental noise. The proposed method, thus, is dedicated to removing such shot noise from the measured spectra. More analysis of these noise sources can be found in the part of Supplementary discussion-noise type analysis.

In the ‘*Supplementary disscusion*’ part we add *Noise type analysis* to show more details of the noise type we are addressing.

Reviewer #2 (Remarks to the Author):

I would like to thank the authors very much for their very accurate and comprehensive responses to my comments and questions. The authors answered all of these in a convincing manner and resolved my criticisms to my complete satisfaction. I recommend acceptance of the revised manuscript in its present form.

Re: We thank the reviewer for the recommendation.

Reviewer #3 (Remarks to the Author):

The authors have satisfactorily addressed my concerns, and clarified the previously unclear points. I will appreciate if the authors include such clarifying explanations in the manuscript as well (some has been incorporated already), so that readers not from Raman background landing on the paper from microscopy/nanoscopy perspective have an easier time understanding and appreciating the paper.

Re: We thank the reviewer for the positive comments. We have tried our best to revise the manuscript to improve the readability for readers not from Raman background.

Reviewer #1 (Remarks to the Author):

I wish to express my gratitude to the authors for their comprehensive investigation regarding the noise property in their measurements. Nevertheless, I find myself still seeking clarity from the data presented.

1.
Given that both the signal and background distribution in the repetitive measurement ought to conform to a Poisson distribution, the expected fluctuation for either the signal or background should be the square root of them. It appears somewhat puzzling to this reviewer that the results do not align with this foundational probability distribution. Moreover, the observed ratio of the Si signal at high to low power stands at approximately 30, whereas the incident power ratio approaches 55.

Regarding Equation R1, R2, and subsequent noise equations, there appear to be discrepancies. For instance, R1 might be better represented as:

SN(Au) = (Npl + Nbg + Ndark + Nread)^(1/2)
Where:
Npl: Electron count from photoluminescence
Nbg: Electron count from the background
Ndark: Dark count
Nread: Readout noise (electron count introduced during readout)

Given the absence of clear definitions for x, i, and N in Eq.R3, my interpretations could potentially be incorrect. I have presumed x to signify the count at a pixel, i as the measurement number, and N as the total number of measurements.

2.
Concerning Fig.R2, the delineation between noise and the sum of background and photoluminescence is somewhat ambiguous. Regardless of noise, signals will invariably fluctuate, following the Poisson distribution. The SVD result appears to indicate an expected value, which is not derived from the separation of signal and noise. This might be a mere semantic discrepancy, yet I would suggest refining the phrasing to preclude any potential confusion. Furthermore, the dark count and readout noise are inherently additive, implying a non-zero average. The assertion that their effects were distinctly separated in the results of Fig.R2 seems somewhat perplexing.

3.
Fig.R3 seems to echo the issues found in Fig.R1. The signal distributions do not appear to adhere to the Poisson distribution.

4.
Per this reviewer's observations, while there is no disputing the efficacy of the authors' methodology in refining the spectrum, I would exercise caution in attributing this solely to the elimination of instrument noise. Given that the signal follows a Poisson distribution and that fluctuation persists even after negating instrumental effects, it might be more apt to suggest "estimating expected values" as opposed to merely "removing noise". This nuance might alleviate some of the ambiguity encountered by this reviewer. Upon resolving the those ambiguities, the manuscript would be well-poised for publication in Nature Communications.

Blue: Reviewers' comments.
Black: Our responses.
Red: Revisions we made.

REVIEWER COMMENTS

Reviewer #1 (Remarks to the Author):

I wish to express my gratitude to the authors for their comprehensive investigation regarding the noise property in their measurements. Nevertheless, I find myself still seeking clarity from the data presented.

Re: We thank the reviewer for all the comments below. We have provided more experimental results and analysis to explain why the noise does not conform to the Poisson distribution.

1.Given that both the signal and background distribution in the repetitive measurement ought to conform to a Poisson distribution, the expected fluctuation for either the signal or background should be the square root of them. It appears somewhat puzzling to this reviewer that the results do not align with this foundational probability distribution.

Re: We thank the reviewer for pointing out this issue. Indeed, the shot noise of the signal and background (stray light) should follow Poisson distribution. Yet, the instrumental noise cannot be solely described by Poisson distribution since the detector noise, which contributes mostly to the overall noise in our case, has different origins whose distribution is manifolds. Konnik et al. [1] thoroughly discussed the noise types of the CCD and CMOS, and broadly divided them into four parts, such as noise arising from the **photons**, **electrons**, **voltage** and **digital signal** as shown in **Fig. R1**. Among them, only *photon shot noise* and *dark current shot noise* conform to the Poisson distribution, other noise sources such as the dark current/signal cannot be described by the Poisson distribution. Wei et al. [2] extended this idea and proposed a noise model defined as:

$$N = K * N_p + N_{read} + N_r + N_q \tag{R1}$$

where K, N_p, N_{read}, N_r, and N_q denote the *overall system gain*, *photon shot noise*, *read noise*, *row noise* and *quantization noise*, respectively. Typically, the read noise contains three terms, i.e., thermal noise, dark noise and source follower noise, which can be described by a *Tukey-Lambda* distribution as Eq. R2:

$$N_{read} \sim TL(\lambda; 0, \sigma) \tag{R2}$$

where λ and σ indicate the shape and scale parameters, respectively, and 0 denotes the average noise intensity. The N_r and N_q are described using zero-mean Gaussian distribution and zero-mean uniform distribution, respectively.

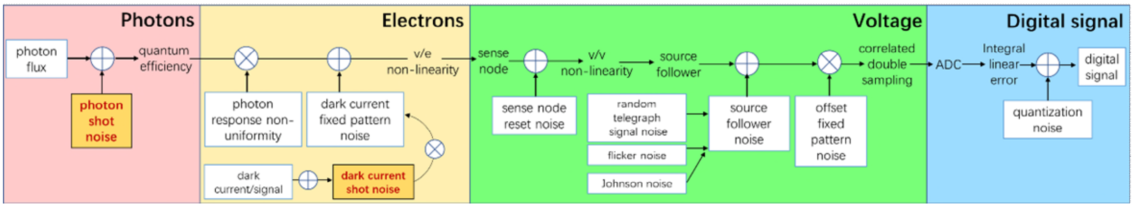

Fig. R1 | The overall pipeline from photons to digital signal via photosensor. Reformatted from Ref.1.

Therefore, since the noise is the joint distribution of Poisson, Gaussian, Tukey-Lambda and uniform distribution, it is reasonable that the calculated standard deviation (σ) is not proportional to the square root of the measured signal intensity in our previous noise measurement experiments, particularly when those noise conforming to Poisson is not the major noise source in our scenario.

References:

1. Konnik, M. & Welsh, J. High-level numerical simulations of noise in CCD and CMOS photosensors: review and tutorial. Preprint at arXiv:1412.4031v1 (2014).
2. Wei, K., Fu, Y., Yang, J. & Huang, H. A Physics-based Noise Formation Model for Extreme Low-light Raw Denoising. CVPR, 2758-2767 (2020).

Moreover, the observed ratio of the Si signal at high to low power stands at approximately 30, whereas the incident power ratio approaches 55.

Re: Thanks for pointing out the discrepancy. We have double checked our original data, and found some abnormal values in the Raman spectra of Si wafer acquired with high laser power. Therefore, we re-measured the Au film and Si wafer Raman spectra with different laser power (**Fig. R2(a)**) using the repetitive line-scan measurement approach as we used previously. We then re-analyzed the data and update our results accordingly.

The new results are as follows:
Low laser power: neural density filter (ND) =150, 1.2 mW (laser line), laser power on each pixel in line scan mode: 3 μW (dividing the laser line power by 400). Mean Si peak signal is ~0.8 counts.
Moderate laser power: ND=220, 35.0 mW, laser power on each pixel in line scan mode: 87.5 μW. Mean Si peak signal is ~22.2 counts.
High laser power: ND=255 (full power), 58.0 mW, laser power on each pixel in line scan mode: 145 μW. Mean Si peak signal is ~41 counts.

The laser power ratio is 1 : 29.2 : 48.3 and the Si peak intensity ratio is 1 : 27.8 : 51.3. This result indicates that the signal intensity increases linearly with the laser power. We also re-calculate the standard deviation of Au and Si Raman spectra and update the results accordingly (**Fig. R2(c)**). Importantly, the new results are also consistent with our previous conclusion, i.e., the signal shot noise will not bring obvious change to the overall noise in our weak signal-to-noise ratio (SNR) scenarios.

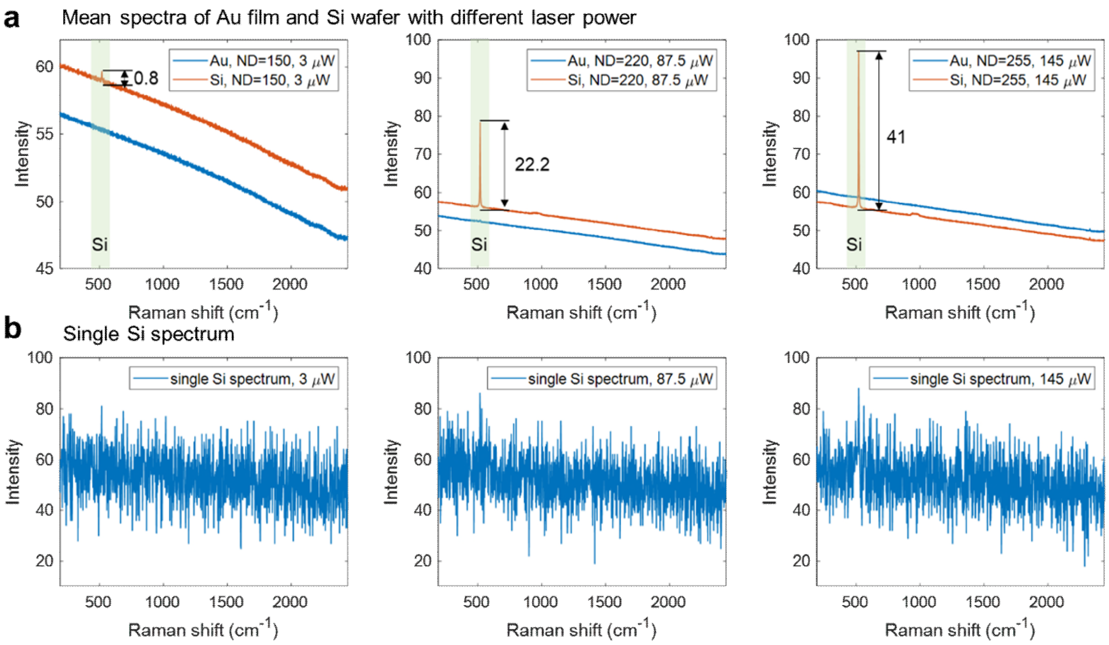

c Standard deviation analysis

	3 μW/pixel		87.5 μW/pixel		145 μW/pixel	
	Au	Si	Au	Si	Au	Si
Si band	8.81	8.93	8.87	9.05	8.91	9.13
Overall<br>Exclude Si band	8.82	8.92	8.86	8.90	8.87	8.90

Fig. R2 | (a) Mean Raman spectra of Au film and Si wafer with different laser power. Each mean spectrum is averaged over 40,000 spectra acquired by line-scan measurement of the same position for 100 times repetitive line-scans, and each line gives 400 spectra. (b) Single Si wafer spectrum acquired with different laser power in line-scan mode. (c) Corresponding standard deviation analysis of Au and Si Raman spectra. Instrument: Raman-11. Objective: 50x, air-dry, NA=0.45. Laser source: 523 nm.

We also check the laser power-signal strength ratio at relatively high laser power condition. For that we changed to the confocal single laser spot mode. Now, with the improved SNR, we can better observe the linear relationship of the laser power and the signal intensity of the Si Raman spectra **(Fig. R3)**.

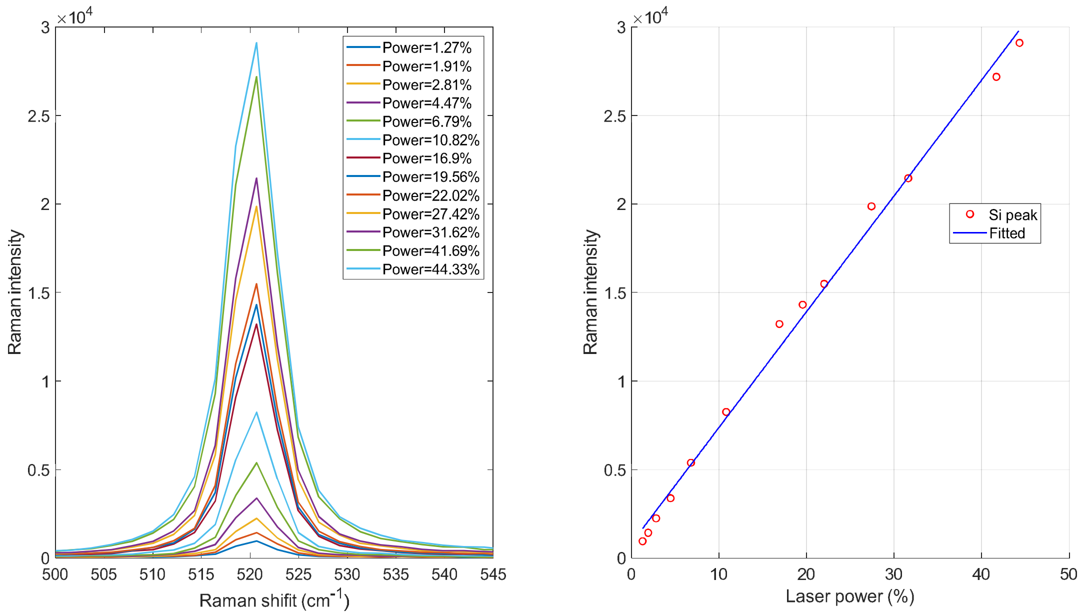

Fig. R3 | Left: Si Raman spectra acquired with different laser power. Right: the relationship of laser power and maxima Si peak intensity. Instrument: Raman-11, 532 nm, the full laser power is ~58 mW, objective: air dry, 50×. Note that these spectra are acquired in the confocal single laser spot mode, so the signal to noise ratio is much better that that obtained in line-scan mode.

Changes: We updated the new results of **Fig. R2** in the supplementary information accordingly.

Regarding Equation R1, R2, and subsequent noise equations, there appear to be discrepancies. For instance, R1 might be better represented as:

$SN(Au) = (N_{pl} + N_{bg} + N_{dark} + N_{read})^{\wedge(1/2)}$
Where: N_{pl}: Electron count from photoluminescence
N_{bg}: Electron count from the background
N_{dark}: Dark count
N_{read}: Readout noise (electron count introduced during readout)

Given the absence of clear definitions for x, i, and N in Eq. R3, my interpretations could potentially be incorrect. I have presumed x to signify the count at a pixel, i as the measurement number, and N as the total number of measurements.

Re: We thank the reviewer for pointing out this problem. Yes, the reviewer is correct regarding on the calculation of standard deviation (σ, Eq. R3 in our previous response letter). To avoid misunderstanding, we further define σ as follows:

$$\sigma = \sqrt{\frac{1}{N-1}\sum_{i=1}^{N}\sum_{j=1}^{M}(x_{i,j}-\mu_j)^2}\ ;\ \mu_j = \frac{1}{N}\sum_{i=1}^{N}x_{i,j} \quad \text{(R3)}$$

where i is the measurement number, N is total number of measurements, j is the wavenumber index, M is total number of wavenumbers, μ_j denotes the mean intensity at j-th wavenumber of

all the measurements.

We also agree with the reviewer's equation listed above when defining N as the *photon/electron counts*. However, the N in Eq. R1 and R2 in our previous response letter means "*noise*", instead of "*counts*", which we wish to clarify. Our equation means that the total noise is the sum of the individual noise considering they are additive. In view of the above discussion, and combining the reviewer's suggestion, here we would like to broadly divide the noise as signal-dependent noise (shot noise arising from background, PL, and Raman signal), and signal-independent noise (noise from the CCD). We then re-write the noise model of the Au as:

$$N^2(\mathrm{Au}) = SN^2{}_{pl} + SN^2{}_{bg} + N^2{}_{ccd} \quad \text{(R4)}$$

Here SN means shot noise (Poisson distribution) and N means other noise (Gaussian, Tukey-Lambda and uniform distribution). SN_{bg} denotes the shot noise arising from the stray light caused by the laser. When the laser power is 0, the slit of the spectrometer is closed, ($SN^2{}_{pl} = 0, SN^2{}_{bg} = 0, \sigma = 8.84$, **Fig. R4**), we have:

$$N^2{}_{ccd} = 8.84^2 = 78.15 \quad \text{(R5)}$$

When the laser power is 145 μW ($\sigma = 9.02$, **Fig. R4**) and suppose N_{ccd} remains stable, we have:

$$SN^2{}_{pl} + SN^2{}_{bg} + N^2{}_{ccd} = 9.02^2 = 81.36 \quad \text{(R6)}$$

Combining Eq. (R5) and (R6) we have:

$$SN^2{}_{pl} + SN^2{}_{bg} = 3.21 \quad \text{(R7)}$$

Since $SN_{pl} \geq 0$, $SN_{bg} \geq 0$, we can estimate that:

$$SN^2{}_{bg} < 3.21,\ SN^2{}_{pl} < 3.21 \quad \text{(R8)}$$

Therefore, we can deduce that in the measured Au spectra with 145 μW laser power, the CCD contributes to the majority (~96%) of the noise variance, and the noise variance coming from the background and PL signal make minor contribution (~4%).

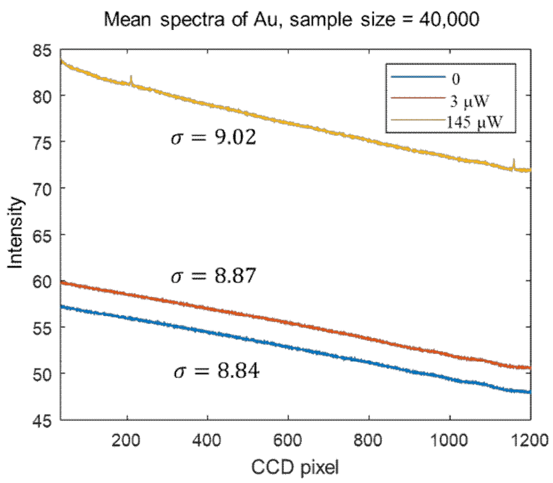

Fig. R4 | Mean Au spectra and standard deviation measured by Raman-11 using line-scan mode at the same positoin for 100 times repetive measurement with different laser power, each laser line consists of 400 spectra (in total 40,000 spectra). Laser = 532 nm, 50× air dry objective (NA=0.45), integration time: 0.5 s/line.

Changes: We revised the above equations and discussion in the "Noise type analysis" part of the Supplementary information accordingly.

2. Concerning Fig.R2, the delineation between noise and the sum of background and photoluminescence is somewhat ambiguous. Regardless of noise, signals will invariably fluctuate, following the Poisson distribution. The SVD result appears to indicate an expected value, which is not derived from the separation of signal and noise. This might be a mere semantic discrepancy, yet I would suggest refining the phrasing to preclude any potential confusion. Furthermore, the dark count and readout noise are inherently additive, implying a non-zero average. The assertion that their effects were distinctly separated in the results of Fig.R2 seems somewhat perplexing.

Re: We appreciate the reviewer for the comment. Indeed, the signal will fluctuate due to the quantum nature of the photons, and should follows the Poisson distribution. From this point of view, we generally agree with the reviewer that the SVD results indicate an expected value. The SVD is similar to principle component analysis (PCA), which can statistically output the engine vectors with descent order according to the explained variance of them. The engine vector with large variance contains more information of the data, whereas the engine vector with small variance thus can be attributed to the noise component. This is the main principle of using SVD to estimate the instrumental noise from the measured Au spectra. Also, the reviewer is correct that dark and readout should give a non-zero average. However, our purpose is to retrieve the amplitude of the noise (i.e., fluctuation) and its pattern in the pixel domain from the original spectra. Therefore, we simply use SVD to separate the "expected value" of the signal and the noise pattern. The latter is then transformed to the frequency domain for data training. Importantly, it is the pattern of the spectral fluctuation rather than the absolute value that is used for noise learning. In this respect, the result of SVD is sufficient for our analysis, even though the absolute value of the noise level has not been extracted.

We become aware that our original description of the "separation of noise and signal" is not fully clear. To avoid misunderstanding, we now revised our description of the SVD method regarding the instrumental noise estimation following the reviewer's suggestion.

Changes:
In the "Methods-Instrumental noise measurement" part of the main text, we changed the sentence
" *Note, the Au film can still emit photoluminescence (PL) signals and reflect light, which need to be eliminated from the Au spectra. To this end, we propose to utilize singular value decomposition (SVD) to separate these contributions from the instrumental noise.*"
to
" *Note, the Au film can still emit photoluminescence (PL) signals and reflect light, which need to be eliminated from the Au spectra. To this end, we propose to utilize singular value decomposition (SVD) to estimate these contributions from the measured Au spectra. This allows us to extract the amplitude of the noise (i.e., fluctuation) and its pattern in the pixel domain from the original spectra, which is used for noise learning.*"

3. Fig.R3 seems to echo the issues found in Fig.R1. The signal distributions do not appear to adhere to the Poisson distribution.

Re: We thank the reviewer for the comment. As discussed in our response to the reviewer's first question, only photon shot noise and readout shot noise conform to Poisson distribution. Most of the noise contribution in our application scenario (CCD readout and dark noise) have different kinds of distribution. Therefore, it is reasonable that the overall distribution of the estimated instrumental noise does not conform to Poisson distribution.

The reviewer might be confused by the calculated standard deviation (σ), which does not increase with the increasing baseline (or background) intensity of the mean Au film spectra as shown in **Fig. R4** (previously Fig. R3 in our last response letter), especially in the high laser power case (145 μW/pixel). This leads to the conclusion that the noise does not conform to Poisson distribution. This is because the increased baseline does not originate from the photoluminescence signal of Au film, as discussed below.

To confirm this, we have measured Raman spectra of Au film and Si wafer with different laser powers (three independent measurements for each case), and presented the mean spectra in **Fig. R5** (Au film) and **Fig. R6** (Si wafer). Each mean spectrum is averaged from 40,000 spectra (100 line measurements on the same position, each line consists 400 spectra). In all datasets, we see certain fluctuations of the baseline intensity in the range of 50-60 counts. However, we do observe abnormal baseline intensity up to 80 counts marked by the arrows. This can be seen for both the data from Au and Si. Therefore, we can conclude that the random fluctuations of the baseline are caused by the CCD itself, independent to the laser power and sample. After discussing with the technician of the company, we think that this may be due to the change of the CCD offset bias. This change of the CCD offset bias will not affect the amplitude of the noise. Therefore, the calculated standard deviation (σ) in **Fig. R4-6** will not significantly change with the baseline fluctuation. Importantly, this suggests that the increased baseline is not from the photoluminescence and thus will not increase

the noise level as expected for shot noise.

We hope this can answer the reviewer's concern.

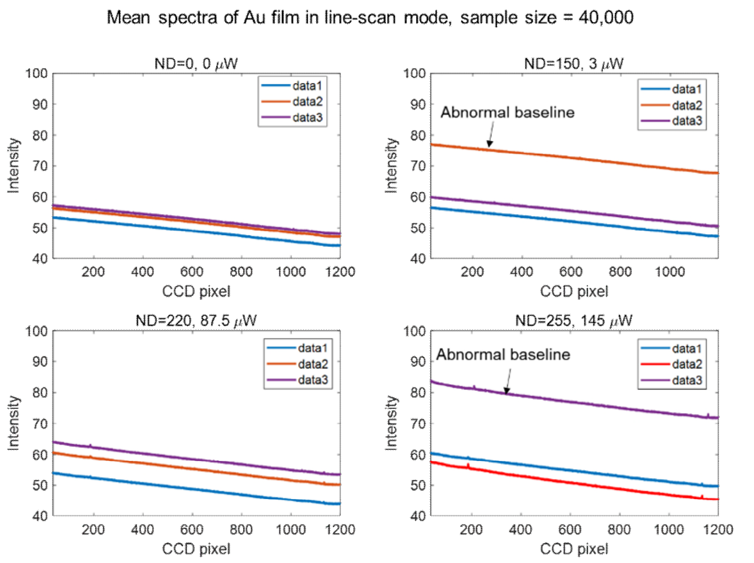

Fig. R5 | Mean Au film spectra measured by Raman-11 using line-scan mode at the same position for 100 times repetive measurements with different laser power. Laser = 532 nm, 50× air dry objective (NA=0.45), integration time: 0.5 s/line. The power in each pixel in indicated in the figure. One can see the change of the baseline intensity due to the change of the offset bias of CCD.

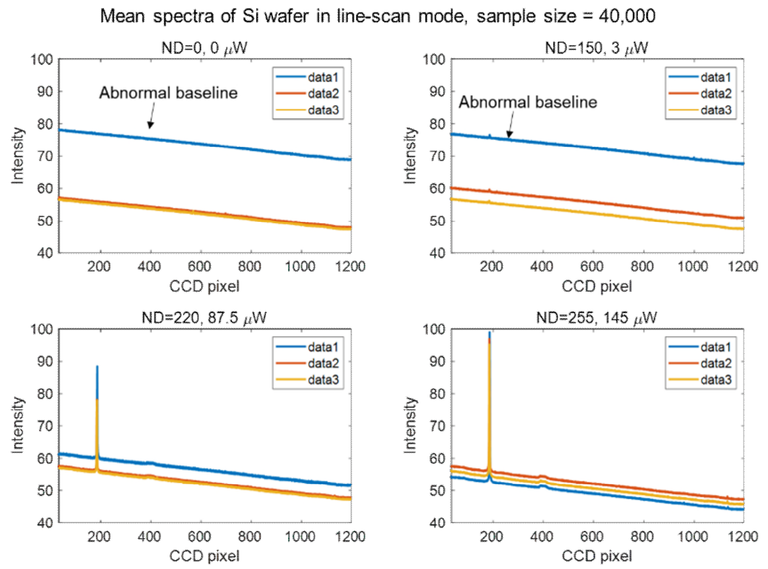

Fig. R6 | Mean Si wafer spectra measured by Raman-11 using line-scan mode at the same positoin for 100 times repetive measurements with different laser power. Laser = 532 nm, 50× air dry objective (NA=0.45), integration time: 0.5 s/line. Similar change of the baseline intensity can be seen.

4. Per this reviewer's observations, while there is no disputing the efficacy of the authors'

methodology in refining the spectrum, I would exercise caution in attributing this solely to the elimination of instrument noise. Given that the signal follows a Poisson distribution and that fluctuation persists even after negating instrumental effects, it might be more apt to suggest "estimating expected values" as opposed to merely "removing noise". This nuance might alleviate some of the ambiguity encountered by this reviewer. Upon resolving the those ambiguities, the manuscript would be well-poised for publication in *Nature Communications*.

Re: We thank the reviewer for the comment.

Changes: We rephrase “removing noise” to “estimating the expected value” as suggested by the reviewer. The revision can be found in the marked-up version of our manuscript.

Reviewer #1 (Remarks to the Author):

The concerns and ambiguities previously raised by the reviewer have been satisfactorily addressed in the revised manuscript. The manuscript can be accepted for publication in Nature Communications.
